# Anti-Inflammatory Activity of Thymol and Thymol-Rich Essential Oils: Mechanisms, Applications, and Recent Findings

**DOI:** 10.3390/molecules30112450

**Published:** 2025-06-03

**Authors:** Custódia Gago, Ana Serralheiro, Maria da Graça Miguel

**Affiliations:** 1 MED—Mediterranean Institute for Agriculture, Environment and Development & CHANGE-Global Change and Sustainability Institute, FCT, Universidade do Algarve, Edf. 8, Campus de Gambelas, 8005-139 Faro, Portugal; cgago@ualg.pt; 2 CCMAR—Centre for Marine Sciences, FCT, Universidade do Algarve, Edf. 2, Campus de Gambelas, 8005-139 Faro, Portugal; aiserralheiro@ualg.pt

**Keywords:** in vitro, in vivo, mechanism, synergism, encapsulation, chemotherapy, livestock

## Abstract

Thymol, a monoterpenoid phenol present in the essential oils of several aromatic plants, has attracted considerable attention for its anti-inflammatory effects, often in combination with other bioactive compounds. This work explores the mechanisms behind the anti-inflammatory activity of thymol and thymol-rich essential oils, summarizing recent experimental findings. Inflammation, a key factor in numerous chronic diseases, can be modulated by targeting essential molecular pathways, such as MAPK, NF-*κ*B, JAK/STAT, and arachidonic acid signaling. Thymol has been shown to influence these pathways, reducing the production of pro-inflammatory cytokines and mediators. Beyond its anti-inflammatory effects, thymol also exhibits a broad range of biological activities, including antimicrobial, antioxidant, and anticancer properties. The applications of thymol and thymol-containing essential oils in therapeutic formulations, food additives, and veterinary medicine are also reviewed. Despite promising preclinical results, challenges such as low bioavailability and toxicity at high doses limit their clinical use. Recent developments in drug delivery systems, such as encapsulation in micro- and nanoparticles, are suggested as strategies to enhance efficacy. Additionally, the synergistic effects of thymol with other natural products are examined, offering the potential for improved therapeutic outcomes.

## 1. Introduction

**Figure 1:**
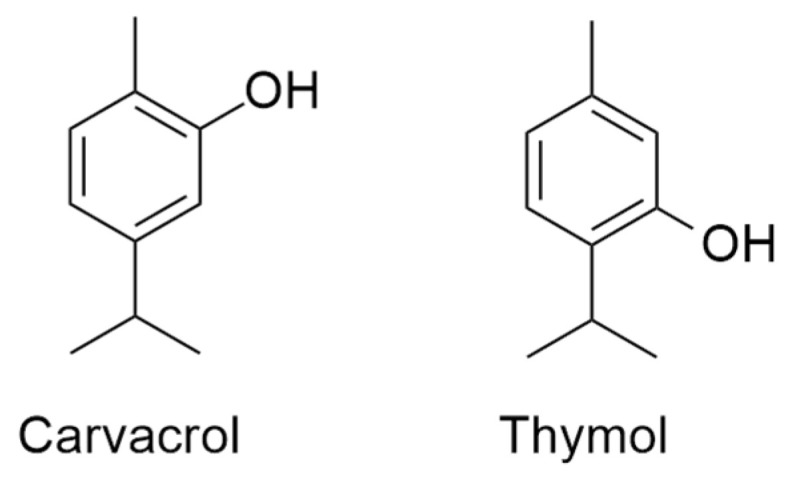
Chemical structures of carvacrol and thymol.

Carvacrol (Figure 1) also known as 2-methyl-5-(1-methylethyl)phenol, 5-isopropyl-2-methylphenol, *p*-cymene-2-ol, 2-hydroxy-*p*-cymene, and iso-thymol (C_10_H_14_O) is a monoterpenoid, an isomer of thymol, belonging to the *p*-menthane class, obtained following the biosynthetic route involving geranyl pyrophosphate (GPP) as precursor and *γ*-terpinene and *p*-cymene as intermediates [1]. Carvacrol can be found in the essential oils isolated from many aromatic plants (e.g., *Origanum vulgare*, *O. compactum*, *O. dictamnus*, *O. microphyllum*, *O. onites*, *O. scabrum*; *Thymbra capitata*; *Thymus vulgaris*, *T. glandulosus*; *Lepidium flavum*; *Citrus aurantium* var. *bergamia* Loisel; *Satureja hortensis*) [2].

Thymol (Figure 1), also known by the chemical names 2-isopropyl-5-methylphenol, 5-methyl-2-isopropylphenol, 3-*p*-cymenol, *p*-cymene-3-ol, 3-hydroxy-*p*-cymene, 6-isopropyl-*m*-cresol, or thyme camphor (C_10_H_14_O), is a carvacrol isomer. Thymol is crystalline (melting point of 49.6 °C and boiling point of 233 °C), colorless, and with a characteristic odor, with a density of 0.970 g/mL. It is slightly soluble in water, soluble in ethanol, diethyl ether, carbon tetrachloride; and very soluble in acetone [3,4].

Thymol is “generally recognized as safe” by the Food and Drug Administration (FDA) and has been allowed by the European Commission for use as a food flavoring because it poses no health hazards to consumers [5]. Thymol can be found in the essential oils of some species of the genus *Thymus* (e.g., *T. vulgaris*), *Origanum*, but also in other plants such as *Ocimum gratissimum* L., *Trachyspermum ammi* (L.), different species of the genus *Satureja* L. and *Monarda* L. (Lamiacaeae), *Lippia gracilis* Schauer (Verbenaceae), *Euphrasia rostkoviana* Hayne (Scrophulariaceae), *Nigella sativa* L. (Ranunculaceae), *Carum copticum* L. and *Oliveria decumbens* Vent (Apiaceae), and *Anemopsis californica* (Saururaceae) [4,5].

Thymol as the main compound in essential oils (EOs) or extracts can be found in several species mainly belonging to the Apiaceae and Lamiaceae families (Table 1). There is only one exception in which the species *Selacia pallescens* belongs to the Celastraceae family.

There are many review articles about the biological properties of thymol. Antimicrobial [5]; anti-inflammatory and wound healing agent [30,31]; anticarcinogenesis; antioxidant; and antispasmodic activities [32] are only very few examples. These characteristics, in addition to those not covered here, make thymol a valuable natural candidate for both pharmaceutical and cosmetic formulations, which has resulted in the creation of patents that were recently reviewed [33]. However, some drawbacks hinder its usage, including drug resistance, toxicity at high dosages, poor water solubility, and poor drug delivery, all of which result in low stability, high hydrophobicity, and poor bioavailability. The same happens for carvacrol. These isomeric chemicals are mutagenic and genotoxic when used in high doses. Therefore, it urges the development of strategies to enhance their use in health settings, for example through the synthesis of hybrid compounds containing their pharmacophores. Peter et al. [34] compiled the research work that has been developed on hybrid compounds containing carvacrol and thymol with considerable antibacterial and anticancer activity.

The present work intends to review the anti-inflammatory activity of thymol and essential oils in which this compound is present.

## 2. Inflammation Mechanism

Inflammation, clinically recognized by signs of heat, redness, edema, pain, and tissue functional loss, is a complex and dynamic innate immunity mechanism that ensures the defense and protection of the human body in response to a disturbance of homeostasis caused by a variety of chemical, mechanical or biological insults. Involving a series of highly regulated molecular and cellular processes, with either local and/or systemic effects, inflammation can lead to the ultimate goal of tissue repair and homeostasis restoration [35,36].

Inflammation can be classified as acute or chronic. Acute inflammation consists of the initial response of the host organism through the release of several inflammatory mediators (e.g. histamine, serotonin, prostaglandins, leukotrienes, chemokines, and cytokines that provide vasodilation, increased blood flow, and vascular permeability as well as neutrophil infiltration and macrophage recruitment to the injury site [37,38,39]. While in acute inflammation, the process terminates with the removal or resolution of the injurious stimulus, in some cases, the organism is unable to achieve tissue repair and overcome the noxious injury, thus perpetuating the inflammatory response [40,41]. Thus, chronic inflammation is characterized by exacerbated and uncontrolled inflammatory responses that are no longer beneficial, damaging tissues and resulting in poor outcomes. Some chronic disease conditions like rheumatoid arthritis, inflammatory bowel disease, atherosclerosis, diabetes, neurodegenerative diseases (Parkinson’s disease, Alzheimer’s disease, and Multiple Sclerosis), obesity, and even cancer have been physiopathologically underlined by chronic inflammation.

**Figure 2:**
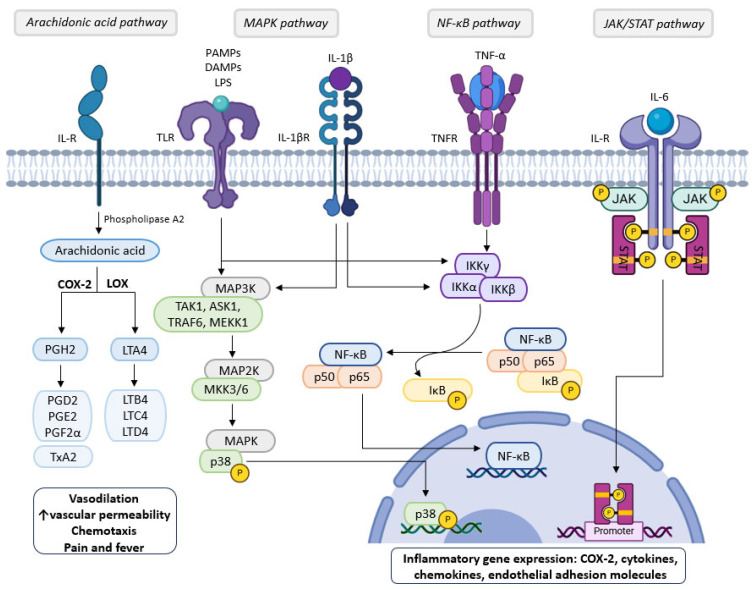
Main intracellular signaling pathways involved in the inflammatory response.

Typically, the mechanisms of inflammation involve the initial activation of cell surface pattern-recognition receptors (PRRs), such as Toll-like receptors (TLRs), that trigger several intracellular signaling events. Pathogen-associated molecular patterns (PAMPs) and/or damage-associated molecular patterns (DAMPs) (Figure 2) are recognized by PRRs that lead to the activation of an intracellular signaling cascade by inducing the nuclear translocation of some transcription factors which stimulate pro-inflammatory cytokines expression and immune cells recruiting [42,43,44].

Upon pro-inflammatory cytokines interaction, especially interleukin-1β (IL-1β), interleukin-6 (IL-6), and tumor necrosis factor-α (TNF-α) (Figure 2), with the corresponding cell membrane receptors, multiple intracellular signaling pathways are propagated due to the activation of a variety of signaling molecules, specific proteins, and transcription factors. Among the various chemical elements involved in the molecular signaling pathways of inflammation, cyclooxygenase-2 (COX-2), mitogen-activated protein kinases (MAPKs), Janus protein tyrosine kinases (JAKs), nuclear transcription factor kappa-B (NF-*κ*B) and signal transducers and activators of transcription (STAT) (Figure 2) play a central role through inflammation escalating [39,40].

The metabolism of arachidonic acid provides the synthesis of several inflammatory mediators such as prostaglandins (PG), thromboxanes, and leukotrienes (LT). The stimulation of the mitogen-activated protein kinases (MAPK) pathway results in a cascade of reactions that culminate in the phosphorylation of p38 which acts as a transcription factor after translocation to the nucleus, where it binds to DNA inducing the production of pro-inflammatory cytokines. Upon membrane receptor stimulation by pro-inflammatory cytokines, the nuclear factor-kappa-B (NF-*κ*B) is activated through NF-*κ*B inhibitor (I*κ*B) phosphorylation and dissociation, being thereafter translocated from cytoplasm to the nucleus where it promotes inflammatory gene expression. The activation of tyrosine kinase receptors mediates the phosphorylation of a Janus kinase (JAK) with the subsequent recruitment of signal transducers and activators of transcription proteins (STAT) that combine with DNA at the nucleus, modulating and regulating the inflammatory response.

COX-2, ciclooxigenase; DAMPs, damage-associated molecular patterns; IKK, I*κ*B kinase; IL-1β, interleukin-1β; IL-6, interleukin-6; IL-R, interleukin receptor; JAK, janus kinase; LOX, lipooxigenase; LPS, lipopolysaccharides, MAP3K, mitogen-activated protein kinase kinase kinase; MAP2K, mitogen-activated protein kinase kinase; PAMPs, pathogen-associated molecular patterns; TLR, Toll-like receptor; TNF-α, tumor necrosis factor alpha, TNFR, tumor necrosis factor alpha receptor; TRAF6, TNF receptor-associated factor 6; TxA2, thromboxane A2.

### 2.1. MAPK Signaling Pathway

MAPKs are members of the protein kinases family which are involved in the regulation of multiple cellular processes, namely, gene expression, apoptosis, survival, mitosis, proliferation, differentiation, inflammatory responses, and stress [45]. Several extracellular stimuli such as oxidative stress, growth factors, inflammatory cytokines, DNA damage, and heat shock are able to activate MAPK signaling. Thus, dysregulation or the induction of functional disturbance of these cascades is commonly related to the induction and progression of many diseases, including inflammatory and autoimmune conditions [46]. MAPKs are subdivided into three main subfamilies: extracellular signal-regulated protein kinases (ERK1 and ERK2) which mediate pro-survival signaling; N-terminal kinases c-Jun (JNK1, JNK2, and JNK3) involved in controlling cell growth, differentiation, and apoptosis; and p38 MAPKs (p38α, p38β, p38γ, and p38δ) that participate in diverse cellular responses like differentiation, inflammation, cell death and tumorigenesis [45,47]. Activation of MAPKs leads to p38 phosphorylation that regulates the activation of transcription factors implicated in the inflammatory response [44,45].

### 2.2. NF-κB Signaling Pathway

**Figure 3:**
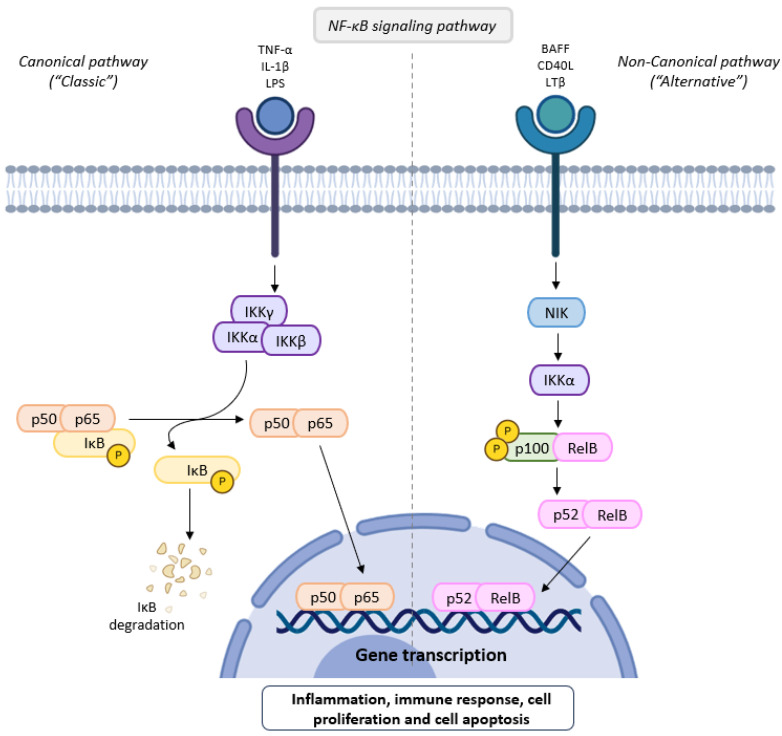
Canonical and non-canonical nuclear factor-kappa-B (NF-*κ*B) signaling pathway.

NF-*κ*B is a group of five transcription factors (p50, p52, p65, RelB, and c-Rel) that play a key role in immune response, inflammation, and cancer regulation, by controlling the expression of COX-2 as well as the synthesis of pro-inflammatory cytokines (IL-6, IL-8 and TNF-α) (Figure 3) [40,45]. In the inactive cytoplasmatic state, NF-*κ*B proteins are attached to an NF-*κ*B inhibitor (I*κ*B) forming a complex. Following cell membrane receptor stimulation by pro-inflammatory cytokines (IL-1 and TNF-α), the I*κ*B kinase (IKK) complex composed of two catalytic subunits (I*κ*B kinase-α, and I*κ*B kinase-β) and a non-enzymatic regulatory protein (IKKγ or NEMO) is activated, mediating the phosphorylation of I*κ*B with its subsequent dissociation and degradation (Figure 3). As a result, functional NF-*κ*B transcription factors are released from the NF-*κ*B/I*κ*B complex and are translocated from cytoplasm into the nucleus where they bind to DNA, inducing the expression of specific target genes such as cytokines, chemokines, and endothelial adhesion molecules which restimulate and maintain inflammation (Figure 3) [40,41,44].

.

The canonical, also known as “classic”, pathway is induced upon membrane receptor stimulation by tumor necrosis factor alpha (TNF-α), interleukin-1β (IL-1β) and lipopolysaccharides (LPS), leading to NF-*κ*B inhibitor (I*κ*B) kinase complex (IKKγ, IKKα and IKKβ) activation which mediate the phosphorylation and subsequent degradation of the I*κ*B. As a result, NF-*κ*B (composed of p50 and p65 transcriptive factors) is release and translocated to the nucleus, where it binds to DNA and regulates downstream gene transcription. Conversely, the non-canonical or “alternative” pathway is mainly dependent of p100/RelB complex activation, which specifically responds to B-cell activating factor (BAFF), CD40 ligand (CD40L), lymphotoxin beta (LTβ) and receptor activator of NF-*κ*B ligand (RANKL). This cascade is initiated by the phosphorylation of both NF-*κ*B-inducing kinase (NIK) and IKKα that activate p100, promoting its conversion into the p52 active form which is then translocated to the nucleus, together with RelB, to target gene expression. NF-*κ*B signaling regulates several cellular processes involving inflammation, immune response, cell proliferation and apoptosis.

### 2.3. JAK/STAT Signaling Pathway

The JAK/STAT pathway plays an important role in numerous cellular processes which include cell proliferation, apoptosis, and immune regulation. It consists of tyrosine kinases receptors that, following endogenous ligand (cytokines, growth factors, granulocyte-macrophage colony-stimulating factor and interferon) stimulation, mediate the JAK coupling with subsequent recruitment and phosphorylation of the STAT family proteins (STAT1-4, STAT5A, STAT5B and STAT-6) present in cytoplasm, governing the direct communication from transmembrane receptors to the nucleus [48,49]. After phosphorylation, STATs dimerize and are transferred into the nucleus to modulate the expression of cytokine-responsive genes by combining specific DNA elements [48].

### 2.4. Arachidonic Acid Signaling Pathway

The release of arachidonic acid from the cell membrane phospholipids by the action of phospholipase A2 and its subsequent enzymatic metabolism into different inflammatory mediators as prostaglandins (PGs), thromboxanes, and leucotrienes, is one of the most significant pathways of inflammation. Eicosanoids derive from araquidonic acid metabolism involving two enzymatic routes. The first one is mediated by COX-1 and COX-2 enzymes and is responsible for the synthesis of proinflammatory prostaglandins (PGD2, PGE2, and PGF2α) and thromboxane A2; while the second one is catalyzed by 5-lipoxygenase with the production of leukotrienes that enhance vascular endothelium permeability and exhibit chemotactic properties [41,50]. Among the various PGs, PGE2 plays a crucial role in inflammation since it is related to vasodilation, nociception stimulation, and pyrogenetic effect. In addition, PGE2 is involved in the T cell activation that recruits more immune cells to the site of inflammation, amplifying the inflammatory response [50].

## 3. Results and Discussion

### 3.1. Thymol-Rich Essential Oils/Volatiles

Thymol as main compound in EOs or extracts can be found in several species mainly belonging to the Apiaceae and Lamiaceae families (Table 1). There is only one exception in which the species *Selacia pallescens* belongs to the Celastraceae family.

The EOs in which thymol is present, regardless its concentration, are shown in Table S1. The EOs in which thymol has the highest percentage are shown in Table 2. In the same tables (Table S1 and Table 2) it is possible to see the other constituents present in the EOs up to a maximum concentration of 5%. Since in most cases, carvacrol is also present in the oils (Table S1 and Table 2) the thymol and carvacrol duo are always the first constituents represented in the tables, regardless of the concentration of carvacrol. The other constituents are represented in descending order, after carvacrol.

Whenever the extraction method is not hydrodistillation, the method is indicated in the table. If the extraction method is not hydrodistillation, the final product obtained is not called EO but volatiles. Essential oils are also mixtures of volatiles but are called EOs because of the extraction process used. This name is given by convention. The units used to represent the concentrations of the constituents of volatile mixtures (EOs or not) are mostly in percentages obtained by internal normalization of the total area of peaks, in one case in mass/mass [8] and in another case in relative abundance of the chemicals [11].

The EOs of the species of the genus *Thymus* and *Trachyspermum* of diverse origins were the most studied (Table 2). Many other species were also targets of study such as *Achillea millefolium*, *Ferulago angulata*, *Ocimum basilicum*, *Oliveria decumbens*, and *Salacia pallescens*, with thymol at higher concentration in EOs (Table 2). In the EOs of *Atractylodes macrocephala* [51], *Coriandrum sativum* [52], *Isodon melissoides* [53], *Lantana camara* [54], *Lantana rhodesiensis* [55], *Lippia multiflora* [56], *Machilus konishii* [57], *Monarda didyma* [58], *Mosla dianthera* [59], *Nigella sativa* [60], oregano [61], *Origanum compactum* [62], *O. minutiflorum* [63], *O. vulgare* [12,64,65], and *Thymbra capitata* [65] thymol was present but carvacrol predominated (Table S1).

**Figure 4:**
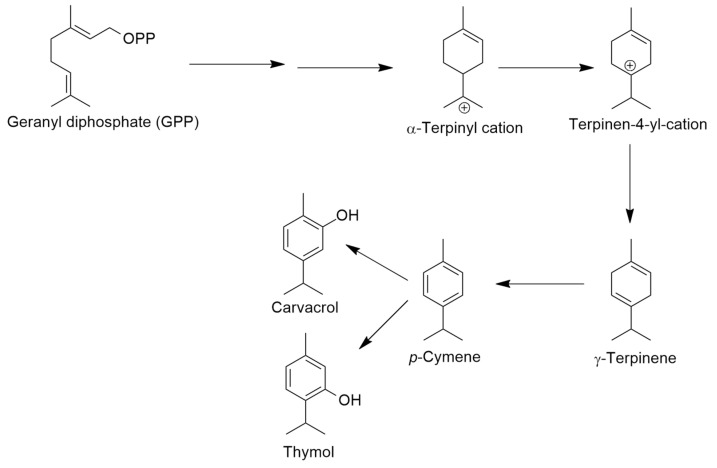
Schematic biosynthesis of γ-terpinene, thymol, and carvacrol (adapted from [66]).

The aerial parts of plants were the material most used for the extraction of volatiles, followed by seeds, particularly for *Trachyspermum ammi*. For *Thymus vulgaris* from Hungary, the impact of harvesting time on the chemical composition was also investigated (beginning and end of flowering). The findings indicated that the percentages of thymol and carvacrol were not significantly impacted, while *p*-cymene increased from the start to the finish of flowering and γ-terpinene decreased during the same time period. This can be explained by the aromatization of γ-terpinene to *p*-cymene as previously reported in *T. vulgaris* plants [66]. Figure 4 shows a very simple schema of the biosynthesis of γ-terpinene, thymol, and carvacrol, adapted from Rudolph et al. [67].

The percentages of thymol in the EOs obtained from diverse populations of *O. heracleoticum* from Italy changed (Table 2), whereas in one case, the authors [11] related the variation of the chemical composition in the EOs to the altitude, in the other case [12], the authors did not report this relationship. *Thymus zygis* collected at different places in Spain also produced EOs with different percentages of thymol (30–54%), there was even one case in which the main component was linalool and not thymol, that is, a linalool chemotype (Table 2). These variabilities may induce different anti-inflammatory responses, which will be analyzed below.

### 3.2. Anti-Inflammatory Activity of Essential Oils or Volatiles with Thymol

The loss of secondary and tertiary protein structure due to external stress factors, such as strong acids or bases, concentrated inorganic salts, organic solvents, or high temperatures is a well-documented basis of arthritic and inflammatory diseases [68]. The anti-inflammatory activity measured through the bovine serum albumin (BSA) denaturation method was done with one EO of *Trachyspermum ammi* seeds [26] from India, and two extracts of the same species, also from India [27] (Table 2). The results are presented in inhibition percentages or IC_50_ values. All samples had better activity than the positive control used (dichlofenac) (Table 2).

Nitric oxide is considered a pro-inflammatory mediator that induces inflammation when there is an overproduction of abnormal factors. The isoform of nitric oxide synthase (NOS) catalyzes the conversion of L-arginine to L-citrulline in human cells, which results in the synthesis of NO. Numerous factors, including inflammatory cytokines, TNF-α, and lipopolysaccharide (LPS), which attaches to the heme group of soluble guanylyl cyclase to produce cGMP, express the inducible NOS [iNOS]. Since NO plays a role in the pathophysiology of inflammatory illnesses of the joints, stomach, and lungs, NO inhibitors represent a significant therapeutic advancement in the management of inflammatory diseases [69].

Several studies have employed the in vitro model of LPS-stimulated RAW264.7 cells to suppress NO generation (Table 2), but the results are presented in a variety of ways that make it difficult to draw conclusions. For example, in [6] the results were presented as the production of nitrites when the cells were subjected to a defined concentration of LPS; production of NO [28] in molarities or in percentage [8,25], in the presence of LPS at different concentrations; and IC_50_ (half maximal inhibitory concentration) values [11,13,15,20]. In [25] the authors also evaluated the NO inhibition capacity of the main individual constituents of the EO and they verified that thymol along with the EO had the best activity in contrast to β-pinene and *p*-cymene, which had the lowest activities (Table 2). In [11] the EOs obtained from several collective samples of the same species showed different abilities to inhibit the production of NO and, in the samples where thymol was present at higher concentrations, the IC_50_ values ranged from 78.62 and 128.7 µg/mL, that is, with significant differences among them. Moreover, in those samples in which carvacrol dominated in the EOs, there were two samples with the best activities but there was one sample with the worst ability to inhibit the production of NO (170,9 µg/mL), including those with higher concentrations of thymol. These findings make it difficult to draw a relationship between the activities and the main EO components, and frequently the results obtained are explained by the potential for antagonism or synergism among the various EO constituents.

The in vitro inhibition of lipoxygenase, preventing the formation of leukotrienes (LT), has been another method used to determine the anti-inflammatory activity of EOs. The results were generally presented as IC_50_ values, with one exception in which the result was expressed as a percentage of inhibition when using 100 µg/mL of sample or positive control (nordihydroguaiatic acid, NDGA). This positive control had much better activity than the EO (100% and 12%, respectively) [17] (Table 2). *Thymus zygis* thymol-rich EO had much better activity than the *Thymus zygis* linalool-rich EO (IC_50_ ranging from 54–73 µL/mL to 299–402 µL/mL), indicating a positive effect of thymol on the inhibitory activity of lipoxygenase (Table 2). The inhibition of cyclooxygenase-2 (COX-2), preventing the formation of prostaglandins (PG) (Figure 2) was also determined (Table 2). The comparison between tetraploid and diploid *Thymus vulgaris* EOs showed that tetraploid had better ability to inhibit COX-2 activity (6.74% at 5 µg/mL) than the diploid one (2.02% at 5 µg/mL) (Table 2) but both far of that of positive control, which at the same concentration (5 µg/mL) the percentage of inhibition was 74.52% [21] (Table 2). *Trachyspermum ammi* EO had also the capacity to inhibit COX-2 [29] (Table 2) with a value of IC_50_ = 4.49 µg/mL. In the same reference, the authors described that carvacrol had better activity than thymol with IC_50_ values of 0.8 and 1.0 µM, respectively. In the same assay, the inhibition of prostaglandins (PGE2) production employing an in vitro model of LPS-stimulated RAW-264.7 macrophages was also evaluated and the activity found was dose-dependent (Table 2). The inhibition of pro-inflammatory cytokine IL-6 production by *Salacia pallescens* EO was also studied using an in vitro model of LPS-stimulated RAW-264.7 macrophages (Table 2). Using 400 µg/mL of EO as a pretreatment decreased IL-6 production ninefold, whereas using 50 µg/mL, decreased it by 1.4 times. LPS-activated THP-1 macrophage cells in the presence of *Thymus vulgaris* EO were able to decrease the production of the pro-inflammatory cytokines IL-6, IL-1β (Figure 2), and TNF-α, but without effect on the pro-inflammatory expression at the mRNA and protein levels [18] (Table 2), in contrast to [19] in which a decrease of expression of IL-1β in the liver and cerebellum, and IL-6 in the hippocampus compared to control in male C57BL/6J mice with 8 (young)- and 53 (old)-week Male C57BL/6J mice and lower expression of *p65* and *p50* (two transcription factors of NF-*κ*B) (Figure 3) than the control (Table 2).

Most in vivo assays used the methods of carrageenan-induced intraplantar edema, croton oil-induced ear edema, phenol-induced ear edema, and formaldehyde-induced arthritis (Table 2). The EO of the aerial parts of *Ferulago angulata* produced 66% inhibition of ear edema induced by croton oil in male Swiss mice at 100 and 200 µL/kg, less active than the positive control (indomethacin) with an inhibition of 82% at 10 mg/kg [7] (Table 2). Nevertheless, the topical application (1 mg/ear) of the EO of *Thymus atlanticus* was able to reduce the ear edema induced by phenol in Wistar albino rats by 19.39%, better than indomethacin that reduced 14.71% [14] (Table 2). This result cannot be compared to that of [7], due to several factors: the type of inflammatory inducer, the breeding animals, the composition of the EO, and the administration type. Conclusions about the effectiveness of thymol-rich essential oils’ anti-inflammatory properties are challenging due to all of these factors. The carrageenan-induced paw edema of Swiss albino mice test was used to test for in vivo anti-inflammatory activity [22] under identical conditions, with the exception that the EO sample (*Thymus vulgaris*) had a distinct chemical profile, despite thymol being the main monoterpenoid in both cases (Table 2). The EO with a higher percentage of thymol showed better activity, albeit much less than the positive control (diclofenac), where 10 mg/kg reduced the thickness of Swiss albino mice’s paws after 6 h under the action of carrageenan, while only 100 to 400 mg/kg of the EO caused a reduction of the paw edema (Table 2). *Thymus linearis* EO had a 67% thymol content, and the anti-inflammatory action against to the effect of carrageenan in the Swiss albino mice’s paw differed significantly from that of the positive control (Table 2), revealing that ibuprofen had much better anti-inflammatory activity than the EO.

Since inflammation is a complex molecular process, there are various ways to assess the anti-inflammatory activity, as was just mentioned. Complementing this context is the intricacy of the EOs’ chemical composition, which varies for the same species based on a number of variables (Table 2). All of these factors may render the use of EOs less effective than the pure chemical, such as thymol, which has been shown in diverse experiments to be more effective against inflammation than either *p*-cymene or γ-terpinene (Table 2). The next sections of this article evaluate and summarize studies made with thymol to investigate its anti-inflammatory properties and occasionally compare it to other natural substances.

## 4. Thymol

### 4.1. In Vitro Anti-Inflammatory Activity of Thymol

Human embryonic kidney HEK293 cells were used by Erzurumlu et al. [70] to study the potential protective benefits of thymol against inflammation-mediated lysosomal stress in the LPS-induced acute kidney inflammation model. LPS was given to HEK293 cells for 24 h in order to simulate inflammation. After that, cells were exposed to different thymol concentrations (25, 50 and 100 μM). TNF-⍺, Nf-*κ*B and phospho-Nf-*κ*B protein levels, and other parameters were examined [autophagy-related Beclin-1, autophagy-related 5 (Atg5), sequestesome 1 (p62/SQSTM1) and microtubule-associated protein 1A/1B-light chain 3 (LC3-I/II), ubiquitin proteasome system-associated polyubiquitin, caspase-3 linked to cell death, and poly (ADP-ribose) polymerase (PARP-1)]. Thymol treatment at 25, 50, and 100 μM doses significantly decreased LPS-induced elevated levels of phosphorylated Nf-*κ*B p65 levels, in particular at 100 μM. The authors also found that LPS-induced acute inflammation caused the suppression of autophagic flux and reduced degradation of polyubiquitinated proteins. The reduction of autophagy and stacking of polyubiquitinated protein by LPS was significantly reversed by thymol administration. Therefore, autophagic flow was blocked by LPS-induced acute inflammation, and thymol protects against LPS-induced lysosomal stress [70].

**Figure 5:**
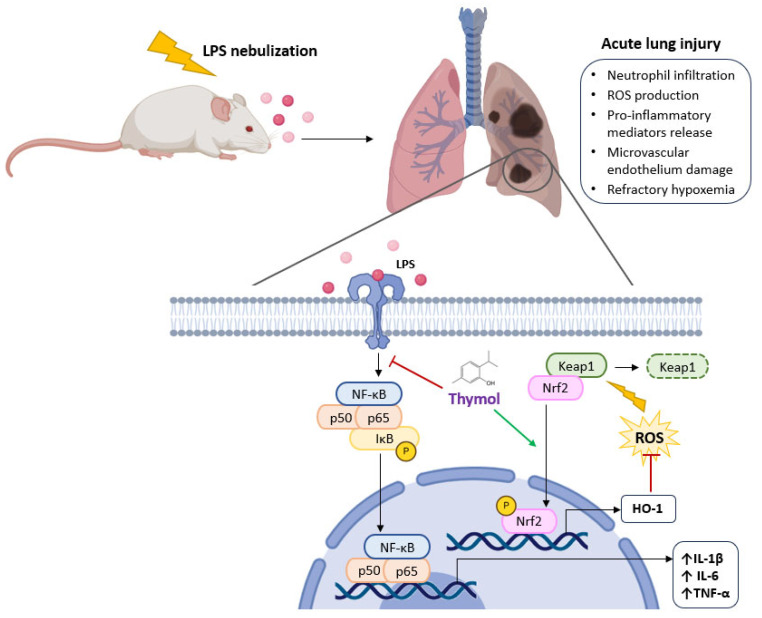
Schematic diagram illustrating the protective effects of thymol in a mouse model of acute lung injury caused by lipopolysaccharides (LPS) exposure. 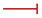 inhibition; 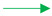 stimulation.

In acute lung injury, there is massive neutrophil infiltration into the lungs, leading reactive oxygen species (ROS) and pro-inflammatory mediators to be released, which damages the pulmonary microvascular endothelium and epithelium and causes refractory hypoxemia, posing a serious risk to a patient’s life. Pathogenesis and treatment of acute lung injury have advanced significantly over the last few years, yet the fatality rate is still high. Therefore, there is an urgent need for a fresh, effective therapeutic [71]. Wan et al. [71] studied thymol’s preventive properties in a mouse model of lung damage caused by LPS and they found that thymol (100 mg/kg) treatment either prior to or following LPS markedly ameliorated pathological alterations in lung tissues in mice that were exposed to LPS. Thymol also reduced the amount of proteins in the bronchoalveolar lavage fluid and the release of TNF-α and IL-6. Furthermore, thymol significantly inhibited the reduction of superoxide dismutase (SOD) activity and prevented LPS from raising malonaldehyde (MDA) and myeloperoxidase (MPO) levels. MPO activity in tissues is an indicator of neutrophil infiltration. Subsequent research revealed that thymol successfully prevented the lung’s NF-*κ*B activation. Similar results were also found by Yao et al. [72] added to the up-regulation of the expression of Nrf2 and HO-1. In mice, Nrf2 is necessary for defense against acute lung injury. Furthermore, pulmonary fibrosis may be prevented by Nrf2. According to the results of both teams [71,72] thymol prevents acute lung injury brought on by LPS by blocking NF-*κ*B and triggering the Nrf2 signaling pathway, implying that thymol might help treat acute lung damage (Figure 5).

Thymol appears to prevent lung damage caused by LPS by blocking the nuclear factor-kappa-B (NF-*κ*B) signaling pathway with the subsequent reduction of pro-inflammatory cytokines release, and also by up-regulating the expression of nuclear factor erythroid 2-related factor 2 (Nrf2) and heme oxygenase-1 (HO-1) that exhibit anti-oxidative effects. IL-1β, interleukin-1β; IL-6, interleukin-6; I*κ*B, NF-*κ*B inhibitor; Keap1, Kelch-like enoyl-CoA hydratase-associated protein 1; ROS, reactive oxygen species.

Diverse cell models have been used to evaluate the anti-inflammatory activity of thymol or their standardized extracts on human airway epithelial cell lines. Standardized hydroalcoholic extract of thyme (0.3% thymol) was assessed on primary human airway (bronchial/tracheal) epithelial cell lines (HBEpC/HTEpC), in a model of lung inflammation induced by LPS. Standardized hydroalcoholic extract of thyme (0.3% thymol) showed anti-inflammatory activity by decreasing the NF-kB p65 and NF-kB p52 transcription factors protein levels followed by the decrease of pro-inflammatory cytokines IL-1β and IL-8, and Muc5ac secretion, in human normal bronchial and tracheal epithelial cells [73]. Muc5ac is a gel-forming mucin that is elevated in patients with lung diseases, such as asthma, chronic obstructive pulmonary disease, and cystic fibrosis. The *muc5ac* gene is up-regulated by LPS [74]. This experiment supports the findings of Zhou et al. [74], who suggested that thymol might be used to treat inflammatory chronic disorders like asthma. However, this is supplemented by the fact that thymol can also be beneficial in cases when mucus hypersecretion is clogging airways [74].

**Figure 6:**
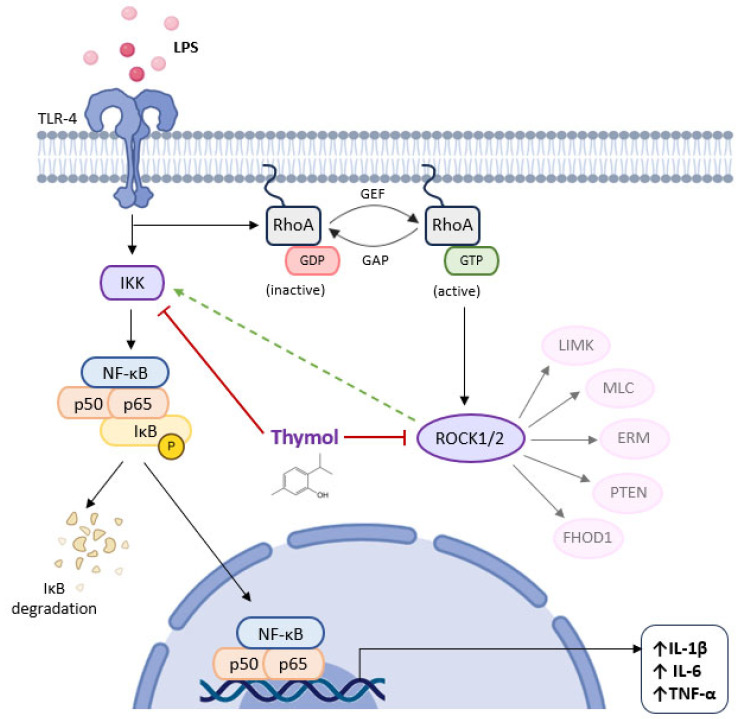
Schematic diagram illustrating the suppressive effects of thymol on lipopolysaccharides (LPS)-induced inflammation and its possible interplay with Ras homolog family member A/Rho-associated coiled-coil containing protein kinase (RhoA/ROCK) signaling pathway. 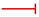 inhibition; 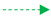 potential stimulation.

The protective effects of thymol on the inflammatory reactions caused by LPS in the human peritoneal mesothelial cell line (HMrSV5) were shown through the inhibition of the synthesis of cytokines, including α-smooth muscle actin (α-SMA), MCP-1, IL-6, and TNF-α, and suppression of the NF-*κ*B p65, IKK, and I*κ*Bα phosphorylation, as well as RhoA and ROCK activation and Toll-like receptor 4 (TLR4) expression (Figure 6). Finally, thymol suppressed the TLR4-mediated RhoA-dependent NF-*κ*B signaling pathway, thereby preventing LPS-induced inflammation in HMrSV5 cells. According to the authors’ research, thymol shows promise as a treatment for peritonitis [74].

Thymol decreases the synthesis of several pro-inflammatory cytokines through the suppression of the nuclear factor-kappa-B (NF-*κ*B) signaling pathway by inhibiting the activation of NF-*κ*B inhibitor kinase (IKK). In addition, it seems that thymol also reduces the RhoA and ROCK expression, preventing the activation of NF-*κ*B via the RhoA/ROCK signaling pathway.

ERM, Ezrin-Radixin-Moesin; FHOD1, formin homology 2 domain containing 1; GAP, GTPase-activating protein; GDP, guanosine diphosphate; GEF, guanine nucleotide exchange factor, GTP, guanosine triphosphate; IL-1β, interleukin-1β; IL-6, interleukin-6; I*κ*B, LIMK, LIM kinase; MLC, myosin light chain; NF-*κ*B inhibitor; PTEN, phosphatase and tensin homolog; TLR-4, toll-like receptor-4; TNF-α, tumor necrosis factor alpha.

The anti-inflammatory properties of thymol (10, 20, and 40 mg/kg) were studied in vivo using LPS-induced mice endometritis. In addition, the molecular mechanism and targets of thymol were also investigated in vitro, using RAW264.7 cells. Thymol significantly reduced MPO activity, TNF-α, and IL-1β production in mice in a dose-dependent manner and LPS-induced pathological damage in vivo. The expression of the Toll-like receptor 4 (TLR4)-mediated NF-*κ*B pathway (Figure 2) was also investigated and the results demonstrated that thymol administration suppressed the expression of the TLR4-mediated NF-*κ*B pathway. In vitro, it was found that thymol reduced the production of COX-2, IL-1β, TNF-α, and iNOS in LPS-stimulated RAW264.7 cells in a dose-dependent manner [75].

Neurodegenerative disorders are among the costliest illnesses in both industrialized and developing countries. Most neurodegenerative diseases start with a long-lasting inflammatory reaction. When inflammation occurs, glial cells, important immune cells in the brain, produce and release ROS and NO. At low levels, NO acts as a vasodilator and neurotransmitter, nonetheless, it is believed to have a critical role in the pathogenesis of stroke, demyelination, and other neurological diseases when it is present in higher concentrations [76]. Javadian et al. [76] focused on comparing the anti-inflammatory effect of methanolic extracts of oregano aerial parts and thymol on LPS-induced neural cells, glial (combination of microgial and astrocytes) and microglial cells obtained from primary glial cell cultures prepared from Wistar rats. In the LPS-treated mixed glial cell, the production of NO was lower when thymol (0.03 mg/mL) was used than in oregano extract (2.7 mg/mL). On LPS-stimulated microglial cells, the results were similar, that is, thymol reduced much more the production of NO than the oregano extract. In addition, oregano extracts had anti-inflammatory effects on the activated mixed and microglial cells by inhibiting inducible isoform of nitric oxide synthase (iNOS) and TNF-α expression. This assay was not performed with thymol, nevertheless, this monoterpenoid was also present in the oregano extracts with concentrations ranging from 13 and 25 μg/g of the dried mass of the stem and leaves, respectively [76].

**Figure 7:**
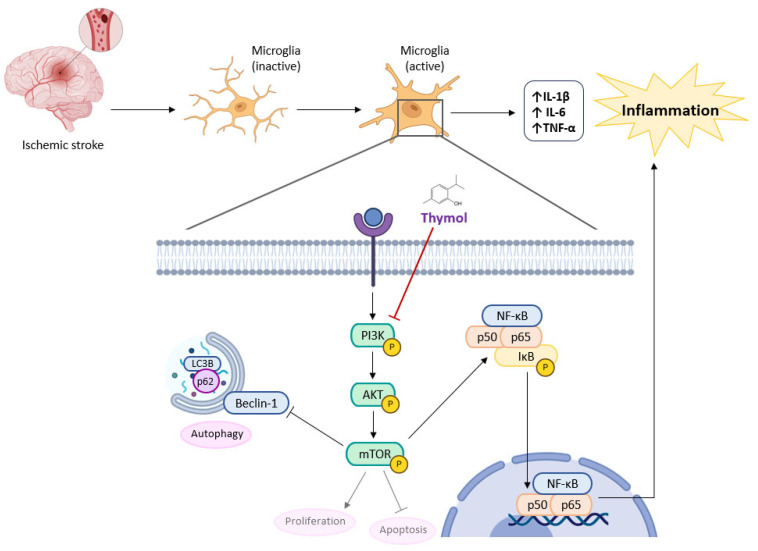
Schematic diagram illustrating the inhibitory effects of thymol in microglia-mediated neuroinflammation subsequent to ischemic stroke, as well as microglial autophagy promotion. 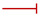 inhibition.

One important element in the development of ischemic stroke is inflammation mediated by microglia. As the resident immune cells in the central nervous system (CNS), microglia can get activated quickly in response to brain injury. Meanwhile, pro-inflammatory cytokines generated by microglia (TNF-α, IL-1β), and IL-6), would worsen secondary brain injury by initiating an inflammatory cascade. Therefore, preventing microglia-mediated inflammation is increasingly being considered as a therapy goal for ischemic stroke [77]. Zhao et al. [77] used a rodent transient middle cerebral artery occlusion (tMCAO) model to simulate ischemic stroke, and LPS to induce the inflammatory response of primary microglia in vitro. Immunofluorescence staining showed that thymol could inhibit microglial inflammation in tMCAO mice. The TNF-α in microglia was significantly reduced following thymol treatment. In in vitro LPS stimulation, thymol (100 µM) treatment markedly reduced IL-1β, IL-6, and TNF-α as well as the expression of *Il1b*, *Il6*, *Tnfa*, *Cox2*, and *Nos2* [77]. To investigate the mechanism involved in the regulation of microglia-mediated neuroinflammation by thymol, the authors examined the key signaling pathways associated with inflammation (Figure 2) and found that after thymol administration, the phosphorylation levels of I*κ*B and NF-*κ*B (p65) significantly decreased. In contrast, the phosphorylation state of STAT3, p38, and ERK after LPS stimulation was unaffected by thymol administration [77]. Moreover, p65 in the microglia nucleus decreased following thymol treatment suggesting that thymol interferes with the translocation of p65 from the cytoplasm to the nucleus (Figure 2) [77]. Following thymol therapy, the primary microglia and penumbra of tMCAO animals also showed a reduction in PI3K, Akt, and mTOR phosphorylation. Moreover, thymol also altered the expression of autophagy-related proteins (p62, Beclin1, LC3B) regulated by the PI3K/Akt/mTOR pathway (Figure 7), therefore, thymol may promote microglial autophagy, hence averting inflammatory responses. According to Zhao et al. [77], thymol might be a neuroprotective agent against ischemic stroke.

The PI3K/AKT/mTOR pathway stimulation is related to NF-*κ*B signaling activation, upregulating the expression of pro-inflammatory cytokines (IL-1β, IL-6, and TNF-α). On the other hand, the PI3K/AKT/mTOR pathway is a central negative regulator of autophagy by phosphorylating and inactivating ULK1 (Unc-51-like autophagy activating kinase 1), which is essential for the initiation of autophagy through the activation of Beclin-1, a cytosolic protein that contributes to initiating the nucleation of autophagosomal membranes.

Thymol seems to inhibit the PI3K/AKT/mTOR phosphorylation, providing not only a neuroinflammation reduction but also a microglial autophagy induction, being a promising neuroprotective agent against ischemic stroke.

AKT, protein kinase B; IL-1β, interleukin-1β; IL-6, interleukin-6; I*κ*B, mTOR, mammalian target of rapamycin; NF-*κ*B inhibitor; NF-*κ*B, nuclear factor-kappa-B; PI3K, phosphoinositide 3-kinase; TNF-α, tumor necrosis factor alpha.

### 4.2. In Vivo Anti-Inflammatory Properties of Thymol in Some Disease Models

The anti-inflammatory activities of thymol were evaluated by Lanzarin et al. [78] using zebrafish (*Danio rerio*) larvae transgenic line (Tg(mpxGFP)^i114^). This assay consisted of the tail section of the larvae that were cut and then exposed to the pro-inflammatory copper (CuSO_4_) to assess the neutrophil migration response, which is an inflammatory marker to the site of injury. The decrease in the number of neutrophils migration (72%) than the control was observed in those samples that had been exposed to thymol 20 µM, nevertheless, diclofenac (positive control) also reduced (55%) that migration as thymol, but at lower concentration (1.5 µM), therefore exhibiting better activity than thymol. The antioxidant and anti-apoptotic activities were also assessed by the authors [78] but using wild-type AB larvae with 72 h post-fertilization. Thymol was able to reduce the production of ROS as well as the expression of apoptotic cells than the control [78].

Acute kidney injury caused by rhabdomyolysis is known as rhabdomyolysis-mediated myoglobinuric renal damage, and it frequently appears following crush syndrome, strenuous activity, drugs, illnesses, and poisons. About 40% of acute kidney injury cases occur in 15% of all rhabdomyolysis patients [78]. Wang et al. [79] used rats in which glycerol injection triggered acute kidney injury linked to rhabdomyolysis. Thymol (20 mg/kg and 40 mg/kg) was administered 24 h prior to the glycerol injection and continued to be administered daily for 72 h following the injection. Several parameters were measured to evaluate kidney injury such as serum creatinine and urea levels, histopathological examination of kidneys, and expression of proliferating cell nuclear antigen. The oxidative stress was also followed by measuring superoxide dismutase (SOD), malondyaldehyde (MDA), and oxidative stress-related nuclear factor erythroid 2 (NF-E2)-related factor 2 (Nrf2), Nrf2/heme oxygenase-1 Nrf2/HO-1 signaling pathways. The anti-inflammatory activity was assessed through the expression of TNF-α, IL-6, monocyte chemoattractant protein-1 (MCP-1), and NF-*κ*B. Glycerol injection increased the oxidative stress as well as the kidney inflammation compared to the control, nevertheless, thymol treatment significantly decreased the expression of IL-6 and TNF-α, (MCP-1) and NF-*κ*B p65 were markedly downregulated (Table 3) compared with the control group. With these results, the authors concluded that thymol may be useful in treating acute kidney injury. Finally, because of its anti-inflammatory and antioxidant properties, thymol may be useful in treating acute kidney injury [79].

Irreversible airflow obstruction and inflammation occur in chronic obstructive pulmonary disease. Games et al. [80] evaluated elastase-induced pulmonary emphysema in mice and the anti-inflammatory effects of *p*-cymene, carvacrol, and thymol. Porcine pancreatic elastase was administered to mice and then they were treated with *p*-cymene, carvacrol, and thymol (each one at 20 mg/kg) by intranasal instillation, 30 min later and again on the 7th, 14th, and 28th days. In the elastase-treated animals, all terpenes reduced alveolar enlargement, macrophages, and the levels of IL-1β, IL-6, IL-8, and IL-17 in BALF, and collagen fibers, matrix metalloproteinase-9 (MMP-9) and p-65-NF-*κ*B-positive cells in lung parenchyma when compared to the animals only treated with elastase. All monoterpenoids attenuated the levels of 8-*iso*-PGF2α, but only thymol was able to reduce exhaled NO (Table 3).

The mechanisms involved in the allergic inflammation in ovalbumin-induced mice asthma showed that pretreatment with thymol (16 mg/kg) reduced the level of ovalbumin-specific IgE, inhibited recruitment of inflammatory cells into airway (eosinophils, neutrophils, macrophages and lymphocytes), and decreased the levels of IL-4, IL-5, and IL-13 (Table 3) in BALF, and reduced the development of airway hyperresponsiveness and blocked the activation of NF-*κ*B pathway, in contrast to ovalbumin that increased all of these parameters. Thymol (16 mg/kg) had a similar response to dexamethasone (2 mg/kg) used as positive control. Based on these results, the authors [74] considered that thymol has the potential to be used as a therapeutic agent in the treatment of allergic asthma, although much more research is needed to confirm the results.

Type 2 helper T cells are responsible for the immunoglobulin-E (Ig-E)-mediated reaction that causes allergic rhinitis. The flavonoids hesperidin and thymol have anti-inflammatory and antioxidant properties. The effects of hesperidin (100 mg/kg) and thymol (20 mg/kg) in Sprague–Dawley rats that had allergic rhinitis caused by ovalbumin were studied by Kilic et al. [81]. Desloratadine was used as a positive control. Among several parameters measured, plasma total Ig-E, IL-5, IL-13 (Table 3), total antioxidant capacity (TAC), and total oxidant status (TOS) levels were included. In the allergic rhinitis group, the levels of Ig-E, IL-5, IL-13, and TOS increased compared to the group not submitted to ovalbumin, however, the animals treated with hesperidin or thymol showed a significant improvement of these parameters, practically similar to that of the positive control. Rats from the allergic rhinitis group showed significant allergic inflammation and severe TNF-α expression at the nasal cavity’s histological and immunohistochemical evaluation, whereas in those treated with thymol, hesperidin or desloratadine only showed modest TNF-α expression and mild inflammatory alterations [81].

Ulcerative colitis is a chronic inflammatory disease of the colon that increases the risk of colorectal cancer. Mice with colitis caused by dextran sulfate sodium (DSS) have been used extensively in ulcerative colitis research because their symptoms (body weight loss, diarrhea, blood in the stool, and mucosal ulcers) are similar to some clinical characteristics of human ulcerative colitis [82]. The administration of drinking water with 2.5% (*w*/*v*) DSS induced colitis in mice and thymol (60 mg/kg) markedly reduced disease activity index scores and recovered the colon length. In addition, it also reduced mRNA expressions of TNF-α, IL-1β, and IL-6 (Table 3) in the colon, downregulated MDA levels, and elevated glutathione (GSH) and SOD levels. 5-Aminosalicylate, the positive control, showed the same ability but at 100 mg/kg. The anti-inflammatory activity was also performed in vitro. The results obtained showed that thymol (100 and 200 µM) significantly inhibited LPS-induced secretion of NO, TNF-α, IL-1β, and IL-6 in macrophages, and suppressed the activation of the NF-*κ*B pathway. Liu et al. [82] concluded thymol is a viable candidate medication for the treatment of ulcerative colitis because it reduces experimental colitis by inhibiting the activation of the NF-*κ*B pathway.

In another experimental model, Wistar rats were given 2 mL of a diluted acetic acid (4%) solution intrarectally, via a flexible plastic rubber catheter, to induce colitis. The anti-inflammatory effect of thymol was evaluated by giving it orally at doses of 10, 30, and 100 mg/kg. On the first day, colitis was induced, and all therapies were administered five days later. Thymol at 100 mg/kg significantly reduced the acetic acid-induced group’s colon tissue’s production of MPO and TNF-α (Table 3) and inhibited the up-regulation of pNF*κ*B p65 protein also caused by acetic acid. Dexamethasone (positive control) at a lower concentration (2 mg/kg) had similar behavior to thymol at a higher concentration (100 mg/kg). Those findings suggest that thymol reduces inflammation in acetic acid-induced rat colitis by blocking the NF-*κ*B signaling pathway as observed in DSS-induced colitis in mice [82] and suppressing the production of TNF-α and MPO [83]. In addition to thymol, Laurindo et al. [84] reported that other phenols (galangin, nobiletin, naringin), terpenes (diosgenin, ginsenoside Rk3, euphol), stilbenes (resveratrol), or curcumin had anti-inflammatory activity, mainly through the NF-kB pathways, with decreases in TNF-α, IL-1β, IL-6, IFN-γ, and COX-2, and increases in the expression levels of occludin, claudin-1, zonula occludens-1, and IL-10. Such results lead the authors to suggest that those compounds can be used in the treatment of inflammatory bowel disease such as the continuous inflammatory process of the colonic mucosa (ulcerative colitis).

As aforementioned, thymol possesses anti-inflammatory in ulcerative colitis acting directly by inhibition of LPS-induced secretion of NO, TNF-α, IL-1β, and IL-6 in macrophages, and suppression of the activation of NF-*κ*B pathway. Nevertheless, in other work, Zhang et al. [85] showed that a mixture of carvacrol and thymol 1:1 (40 µL/kg) orally administered to mice for 14 days following 12 days of DSS exposure was able to mitigate the induced colitis by enhancing *Bifidobacterium pesudolongum* abundance (Table 3) in colon, that in turn, such induced an increase of hyodeoxycholic acid (HDCA) and 12-ketodeoxycholic acid (12-KCAC). These secondary bile acids exerted anti-inflammatory effects through a regulatory mechanism in which transmembrane guanylate cyclase 1A expression in the colonic epithelium is decreased by them, hence inhibiting colonic inflammation. Protein kinase G (PKG) was activated by this downregulation, through the elevation of intracellular Ca^2+^ and cGMP levels. Activated PKG subsequently suppressed the mammalian target of the rapamycin (mTOR) signaling pathway, and colon damage caused by DSS was lessened [85]. The same assay performed in a pseudo-germ-free mouse model (mice treated with a broad-spectrum antibiotic mixture constituted by ampicillin, streptomycin, gentamicin, and vancomycin), the mixture of carvacrol and thymol had not exerted any anti-inflammatory effect. With these results, the authors [85] intended to show the importance of gut microbiota in the inflammatory/anti-inflammatory processes.

A condition known as myocardial infarction affects the coronary artery, which supplies blood to the heart muscle. An abrupt blockage of the coronary artery causes irreparable damage to the heart muscle. The pathophysiology of myocardial infarction is the plaque rupture with thrombosis. Inflammatory mediators such as cytokines have a role in the development of atheromatous damage, the rupture of plaques, and intraluminal thrombosis. Therefore, it is thought that inflammation plays a key part in the pathophysiology of myocardial infarction by causing plaques to rupture [86]. Isoproterenol is a sympathomimetic β-adrenergic agonist that induces cardiotoxicity in the form of myocardial infarction in rats. This cardiac damage may be due to the unbalance of highly cytotoxic free radicals, the intracellular release of lysosomal enzymes, or an increase in their activities [86]. The authors aimed to know the anti-inflammatory ability of thymol on isoproterenol-induced myocardial inflammation in rats. The myocardial infarction was induced in rats through the subcutaneous injection of isoproterenol 100 mg/kg at an interval of 24 h for 2 days. The disease was confirmed by elevated levels of serum cardiac troponin-T. The levels of thiobarbituric acid reactive substances (TBARS) in the lysosomal fraction of the heart tissue homogenate were significantly higher in isoproterenol-induced myocardial infarcted rats than in normal control rats. Pre- and co-treatment with thymol (7.5 mg/kg) decreased the levels of TBARS in the lysosomal fraction of the heart tissue homogenate, being significantly lower (0.35) than in isoproterenol-induced myocardial infarcted rats (0.89). When compared to the myocardial of normal control rats, the expression of pro-inflammatory cytokines, TNF-α, IL-6, and IL-1β was raised in the myocardium of rats induced by isoproterenol. The pre-and co-treatment with thymol (7.5 mg/kg body weight) decreased the expression of those pro-inflammatory cytokines (Table 3) [86].

Atherosclerosis, which is frequently linked to oxidative stress and local inflammation, is a major factor in the development of cardiovascular disorders. The production of several proinflammatory cytokines by endothelial cells, such as IL-1 and TNF-α, is stimulated by oxidized low-density lipoprotein (ox-LDL). These cytokines then trigger the expression of adhesion molecules and chemotactic factors, which leads to monocyte adherence and migration. To aid in the absorption of ox-LDL, monocytes go into the artery wall and develop into macrophages. These macrophages release a variety of proinflammatory cytokines in addition to changing into foam cells. The components of the extracellular matrix are broken down by matrix metalloproteinases (MMPs), which allow vascular smooth muscle cells (SMCs) to migrate more easily. The pathophysiology of atherosclerosis and neointima formation may be influenced by MMP production and SMC migration. Numerous risk factors, including dyslipidemia, are linked to atherosclerosis. Natural supplements, a balanced diet, and regular exercise are becoming more and more important ways to prevent or reduce the disease’s progression [87]. These authors evaluated thymol’s in vivo antioxidant activity and looked at how it affected atherosclerosis and hyperlipidemia caused by a high-fat diet. For eight weeks, New Zealand white rabbits were fed either standard chow, a high-fat and high-cholesterol diet, or supplemented with thymol at 3 or 6 mg/kg/day. Serum lipid parameters, proinflammatory cytokines, and diverse inflammatory markers among other parameters were evaluated. In what concerns the proinflammatory cytokines, IL-1β, IL-6, TNF-α, and TNF-β that increased in the group with a high-fat and high-cholesterol diet, the supplementation with thymol at 3 or 6 mg/kg/d decreased the levels of these proinflammatory cytokines (Table 3). The presence of thymol (3 and 6 mg/kg/day) also decreased the expression levels of atherosclerosis-associated indicators, namely, vascular cell adhesion molecule-1 (VCAM-1), MCP-1, and MMP-9, shifting the effect of high-fat and high-cholesterol diet [87]. These authors chose to use the rabbit model because it has been useful in studying how food and statins affect the development of atherosclerosis and the decrease of cholesterol [87]. The same animal model was used by Kong et al. [88] to study the effect of kaempferol on atherosclerosis and the results were similar to those found for thymol, that is, kaempferol showed anti-atherosclerotic effect by modulating the gene and protein expression of inflammatory molecules, but at 30 mg/kg, 150 mg/kg/day.

Endometriosis is a chronic inflammatory disease that is estrogen-dependent and is characterized by the presence and proliferation of endometrial cells outside the uterus, also known as ectopic endometrium. Infertility, fatigue, and chronic discomfort are common signs of endometriosis [89]. Endometriosis and other gynecological disorders can result from imbalances in the estrogen and progesterone ratio and the development of this disease is greatly aided by immunological dysfunction, especially in the pelvic cavity, where the majority of lesions are found. Macrophages accumulate in the peritoneal cavity of women with endometriosis due to the local production of chemotactic chemicals. However, under normal conditions, peritoneal macrophages can destroy endometrial tissue, and this phagocytic mechanism appears to be ineffectual in endometriosis [89]. The peritoneal lining consists of monolayer epithelioid cells, called mesothelial cells (MCs). Upon exposure to leukocytes, they produce pro-inflammatory mediators; activated MCs produce lipid mediators (prostaglandins), growth factors, cytokines, chemokines, and adhesion molecules, resulting in the recruitment of additional leukocytes to the mesothelium [89]. These authors investigated the role of thymol in the treatment of endometriosis using an endometriotic mouse model and Ishikawa cells. In in vivo assays, female mice were subcutaneously administered 17β-estradiol (100 ng/mouse) for three consecutive days. After adequate surgery to induce endometriosis, the animals were submitted to the administration of thymol 30 or 60 mg/kg/day, for three weeks. The authors found that pro-inflammatory cytokine levels, TNF-α, IL-1β, and IL-6, together with neutrophil and macrophage counts, were considerably lower in the thymol-treated group (Table 3). Moreover, in comparison to the control group (without thymol) group, thymol significantly decreased the weight and volume of ectopic tissue, inhibited cell proliferation, and induced apoptosis. Thymol also inhibited the estrogen signaling that leads to the growth of ectopic endometrial cells by competing with estrogen for binding to estrogen receptor 1 (ESR1), whereas progesterone receptor (PGR) and target genes were significantly upregulated. Thymol dramatically inhibited the growth of Ishikawa cells induced by 17β-estradiol [89].

In cases of acute or chronic hepatic failure, hyperammonemia can lead to the illness known as hepatic encephalopathy. Excessive ammonia absorption in the brain and cerebrospinal fluid (CSF) leads to multiple neurological deficits and disturbed brain functioning. As a consequence, astrocytes undergo a variety of functionally significant changes (post-translational protein modifications, senescence, altered gene expression, and RNA oxidation) that can impair glial and neural processes as well as communication. Inflammation may also play a role in the development of hepatic encephalopathy through liver-to-brain pro-inflammatory signaling pathways. Astroglial cells exposed to pro-inflammatory cytokines and ammonia exhibited elevated expression levels of genes implicated in hepatic encephalopathy [90]. The characterization of the neuroprotective potential of thymol (30 and 60 mg/kg, p.o.) against neurotoxicity and cognitive decline induced by thioacetamide in an experimental model of hepatic encephalopathy was performed by Ogaly et al. [90]. Serum ammonia was more elevated in the thioacetamide-induced rats, 50% higher than the negative control without administration of acethamide. Nevertheless, thymol at 60 mg/kg and vitamin E (positive control) at 100 mg/kg were able to decrease the level of ammonia by 20 and 36%, respectively. Thymol increased the expression of the glial fibrillary acidic protein (GFAP) and decreased NF-kB (Table 3). Furthermore, thymol, particularly at 60 mg/kg, enhanced brain cAMP levels, and markedly increased cAMP-response element binding protein (CREB) and brain-derived neurotrophic factor (BDNF) expression at the mRNA and protein levels. BDNF is a neurotrophic factor that has a significant impact on neuronal and cognitive activity and is involved in the control of synaptic plasticity and neural development. When BDNF binds to its receptor, it triggers PI3K/Akt, a downstream signaling pathway leading to the enhancement of CREB. In turn, CREB is a transcription factor that controls a large number of pro-survival genes via binding to the cAMP-responsive element in their promoter region. Thus, CREB plays a regulatory role in various functions, including mitochondrial biogenesis [90]. The authors [90] concluded that thymol had hepatoprotective and neuroprotective benefits against hepatic encephalopathy by reducing hepatotoxicity, hyperammonemia, and brain ATP depletion through its anti-inflammatory properties and by activating the CREB/BDNF signaling pathway.

Parkinson’s disease, a chronic neurodegenerative movement condition, is caused by an accumulation of Lewy bodies containing α-synuclein throughout the brain, dopamine depletion in the striatum, and a gradual and severe loss of dopaminergic neurons in the substantia pars compacta. Oxidative stress and neuroinflammation play a significant role in the onset of Parkinson’s disease. One of the signaling pathways that has been linked to Parkinson’s disease neuroinflammation in the brain is the NF-*κ*B pathway, which regulates inflammation by controlling the expression of pro-inflammatory genes. Moreover, NF-*κ*B regulates inflammasome activity, which in turn triggers the NLRP3 inflammasome and causes Parkinson’s disease patients to release NLRP3-dependent inflammatory cytokines. The NLRP3 inflammasome is a multiprotein inflammatory signaling complex that functions as a molecular driver of the inflammatory response. Toll-like receptors (TLRs) are activated by microbial or damage-associated molecular patterns that may arise from particular cells during inflammation. Accumulation of α-synuclein protein, characteristic of Parkinson’s disease, also stimulates TLR that can activate NLRP3 inflammasome [91]. In turn, this leads to the activation of caspase-1, which stimulates the release of IL-1β. The Nrf2/HO-1 signaling pathway is essential for protecting against oxidative and inflammatory damage by inhibiting NF-*κ*B. GSK-3β is another factor linked to the degeneration of dopaminergic neurons that is characteristic of Parkinson’s disease. GSK-3β is essential for both neurodegeneration and cell apoptosis; activation of GSK-3β causes microglia to become activated and increases the production of inflammatory cytokines, which causes neuroinflammation and degeneration [91]. Abu-Elfotuh et al. [91] studied the effect of sesamol, thymol, wheat grass, and coenzyme Q10 (CoQ10) on the anti-inflammatory, anti-apoptotic, and antioxidant activities, in rats with MnCl_2_-induced Parkinson’s disease. For thymol and sesamol, the rats were treated with thymol and sesamol (30 mg/kg/day and 15 mg/kg/day, p.o., respectively) 1 h before MnCl_2_ (10 mg/kg/day, i.p.) for 5 weeks. In comparison to the control rats, MnCl_2_ caused inflammation by markedly increasing the levels of TNF-α, TLR4, NLRP3, NF-*κ*B, caspase-1, and IL-1 in the brain by 9.1, 97.2, 60.1, 44.75, 51.6, and 6 times, respectively, along with the COX-2 level. Sesamol, thymol (Table 3), CoQ10, WG, or their combination (sesamol + thymol + CoQ10 + wheat grass), however, dramatically reversed such increases in TNF-α level by 61%, 57.4%, 49%, 49%, and 67.3%, respectively; TLR4 level by 73.3%, 62.2%, 44.8%, 56.3%, and 85.8%, respectively; NF-*κ*B level by 60.8%, 48.5%, 55.6%, 34.4%, and 74.3%, respectively; NLRP3 level by 59.9%, 49.7%, 42.7%, 48.9%, 48.8%, and 74.2%, respectively; caspase-1 level by 58.5%, 54.1%, 47.6%, 48.8%, and 69.6%, respectively; IL-1 level by 58.5%, 53%, 50.5%, 37.7%, and 63.4%, respectively; and COX-2 contents by 56.1%, 53.6%, 53.4%, 52.7%, and 36.1%, respectively [91]. Sesamol and thymol demonstrated superior protection against neuronal degeneration and certain behavioral deficits induced by MnCl_2_ compared to wheatgrass or CoQ10, owing in part to the interaction between the *Nrf2/HO-1*, *TLR4/NLRP3/NF-kB*, *GSK-3β*, and *Bax/Bcl2* pathways [91]. Because of their anti-inflammatory properties, other phenols, such as kaempferol, can be regarded as possible natural drugs in Parkinson’s disease treatment [92]. Beyond its benefits on Parkinson’s disease treatment and other inflammatory neuropathologies, this flavonol has also demonstrated anti-inflammatory activity on ischemic brain injury and spinal cord injury [92].

**Table 3:** In vivo anti-inflammatory activity of thymol.

Disease	Disease Induction	Treatment	Results	Reference
Acute kidney injury	Glycerol injection in rats	20 mg/kg and 40 mg/kg 24 h before glycerol administrations continued for 72 h, by gavage	IL-6↓, TNF-α↓, monocyte chemoattractant protein-1 (MCP-1)↓, NF-kB p65↓	[79]
Chronic obstructive pulmonary disease	Porcine pancreatic elastase nasal instillation to mice	20 mg/kg later the administration of elastase and then at days 7, 14 and 28, by instillation	IL-1β↓, IL-6↓, Il-8, IL-17, matrix metalloproteinase-9 (MMP-9)↓, p-65-NF-kB↓, 8-iso-PGF2α↓, NO exhalation↓	[80]
Allergic inflammation	Intraperitoneal (i.p.) injection of ovalbumin at day 0, and on days 21, 22 and 23 the mice were submitted to intranasal inhalations	16 mg/kg per os (p.o.) had similar response to 2 mg/kg of dexamethasone (positive control)	Ovalbumin-specific IgE↓, recruitment inhibition of eosinophils, neutrophils, macropages and lymphocytes into airway, IL-4↓, IL-5↓, IL-13, NF-kB↓	[74]
Allergic rhinitis	Injection i.p. of ovalbumin in rats every other day for 14 days, then administration into both nostrils for 7 days	20 mg/kg through intragastric lavage	IgE↓, IL-5↓, IL-13↓, total antioxidant capacity↓	[81]
Ulcerative colitis	Dextrane sulfate sodium in drinking water to mice	60 mg/kg p.o.	mRNA expression of TNF-α↓, IL-1β↓, IL-6↓. Malondalehyde (MDA) ↓, superoxide dismutase (SOD) activity↓, glutathione↑	[82]
Colitis	Acetic acid intrarectally to rats	100 mg/kg p.o. 5 days after the administration of acetic acid	MPO↓, TNF-α↓, upregulation inhibition of NF-kB p65 protein	[83]
Colitis	Dextrane sulfate sodium in the drink water to mice	40 µL/kg thymol:carvacrol (1:1) p.o. for 14 day following 12 days of dextrane sulfate sodium exposure	*Bifidobacterium pesudolongum*↑, hyodeoxycholic acid (HDCA)↑, 12-ketodeoxycholic acid (12-KCAC)↑, protein kinase G (PKG) activated, mammalian target of rapamycin (mTOR) signaling pathway suppressed	[85]
Myocardial infarction	Isoproterenol injection subcutaneous at an interval of 24 h for 2 days to rats	Thymol (7.5 mg/kg) during pre- and cotreatment	Expression of TNF-α↓, IL-6↓, IL-1β↓. Thiobarbituric acid reactive substances (TBARS) ↓	[86]
Atherosclerosis	Rabbits with high-fat and high cholesterol diet	Rabbits with a diet supplemented with 3 or 6 mg/kg/day	IL-1β↓, IL-6↓, TNF-α↓, TNF-β↓, vascular cell adhesion molecule-1 (VCAM-1) expression level↓, monocyte chemoattractant protein 1 (MCP-1)↓, matrix metalloproteinase 9 (MMP-9)↓	[87]
Endometriosis	Female mice submitted with 17β-estradiol subcutaneously for 3 consecutive days. At the last injection day, the mice underwent surgical induction of endometriosis	30 mg/kg/day or 60 mg/kg/day p.o. for 3 weeks	IL-1β↓, IL-6↓, TNF-α↓, TNF-β↓, neutrophil and macrophage counts↓, estrogen signaling inhibited by competing with estrogen for binding to estrogen receptor 1 (ESR1)	[89]
Hepatic encephalopathy	Thioacetamide i.p. 3 times per week, for 2 weeks to rats	30 or 60 mg/kg p.o., daily for 1 month	Alanine aminotransferase (ALT)↓, aspartate aminotransferase (AST)↓, ammonia↓, brain oxidative stress↓, brain ATP↑, astrocyte swelling↓, brain edema↓, NF-kB protein expression↓, glial fibrillary acidic protein (GFAP) protein expression↑	[90]
Parkinson’s disease	MnCl_2_ i.p. for 5 weeks to rats	30 mg/kg/day p.o., 1 h before Mn administration for 5 weeks	TNF-α↓, TLR4↓, NF-kB↓, NLRP3↓, IL-1↓, COX-2↓	[91]
Autism	Valproic acid to male rats	Thymol 30 mg/kg	Phosphorylated p38 MAPK↓, IL-1β↓, TNF-α↓, Pin1↓, postsynaptic density protein 95 (PSD95)↑, synaptophysin (SYP)↑	[93]
*Aspergillus fumigatus* corneal keratitis	Right eyes of female mice infected with conidia (2.5 μL, 5 × 10^7^ CFU/mL)	50 µg/mL subconjunctival injection 1 day and 2 h before infection and treated with subconjunctival injection twice a day	TLR4↓, MyD88↓, NF-kB↓, IL-1β↓	[94]

↓: decrease; ↑: increase.

Autism spectrum disorder is a heterogeneous neurodevelopmental syndrome typified by repetitive stereotypical behaviors, limited interests, and impairments in social interaction difficulties with social interaction and communication. Inflammation plays an essential role in the pathogenesis of autism spectrum disorder. The etiology of autism spectrum disorder is increasingly supported by evidence of inflammatory dysregulation. Brain tissue and cerebral spinal fluid were shown to have higher levels of inflammatory cytokines, IL-1β, and TNF-α. Brain inflammation in psychiatric disorders is linked to elevated TNF-α and IL-1β in the prefrontal cortex (responsible for social behavior, emotional processing, and decision-making). Furthermore, aberrant synaptic formation is typically induced by inflammation. Other players have been reported as being involved in the autism spectrum disorder: peptidyl-prolyl *cis*/*trans* isomerase, NIMA interaction 1 (Pin1) that recognizes the serine/threonine-proline motif of phosphorylated substrates, and has an important role in inflammation; and p38 MAPK (mitogen-activated protein kinases) (p38 kinase is a proline-directed serine/threonine kinase that belongs to the MAPK family and is activated by environmental stress and inflammatory signals) [93]. Xiong et al. [93] examined the impact of thymol on behaviors resembling autism using a rat model of autism spectrum disorder produced by valproic acid. Through genetic suppression of Pin1, the authors also investigated the impact of Pin1/p38 MAPK on inflammation and synaptic development in a rat model of autism spectrum disorder caused by valproic acid. PSD95 and synaptophysin are indicators of synaptic plasticity involved in synapse formation and reconstruction, as well as pre- and post-synaptic components of synapses. A loss of synapses is typically implied by their diminished expression. Nevertheless, it is still unknown how inflammation controls synaptic growth in rats with valproic acid-induced autism spectrum disorder [93]. The authors also aimed to unveil whether this relationship exists or not. The results showed that in the prefrontal cortex of the valproic acid-treated rat model, there was an increase in the levels of Pin1, phosphorylated p38 MAPK, IL-1β, and TNF-α, while the levels of PSD95 and synaptophysin decreased. However, thymol treatment (30 mg/kg) resulted in a reduction of valproic acid-induced autism-like behaviors, and it also restored the dysregulated levels of Pin1, phosphorylated p38 MAPK, IL-1β, TNF-α, PSD95, and SYP (Table 3). The correlation between Pin1 and phosphorylated p38 MAPK in the group treated with thymol decreased, according to the results obtained in the immunofluorescence experiments. Mechanistically, Pin1 knockdown by RNA interference demonstrated that, in the rat model of valproic acid exposure, Pin1 stimulates inflammation via phosphorylating p38 MAPK [93]. In conclusion, the authors [93] were able to show that thymol enhanced autism-like behaviors in rats with valproic acid-induced autism spectrum disorder through the Pin1/p38 MAPK pathway by lowering inflammation and enhancing neurodevelopment.

After corneal abrasions linked to plant-based materials and contact lenses, fungal keratitis may occur caused by filamentous fungi or yeasts [94]. A corneal epithelial defect allows a pathogenic fungus (*Aspergillus fumigatus*) to infiltrate the cornea, which is identified by TLRs, as pattern recognition receptors (PRRs). After TLR4 binds to its corresponding ligand, it activates the myeloid differentiation factor 88 (MyD88) protein and facilitates NF-*κ*B’s translocation into the nucleus. This triggers the transcription of downstream-related genes and causes macrophages, monocytes, and other cells to produce and release inflammatory transmitters such as IL-1β, TNF-α, and IL-12, as well as chemotactic cytokines (CXCL8, CCL2, CCL3) [95]. Wang et al. [94] demonstrated that thymol (50 µg/mL) prevented the aggregation of inflammatory cells, inhibited the production of the TLR4/MyD88/NF-*κ*B/IL-1β signal (Table 3), and decreased necroptosis and pyroptosis in *A. fumigatus* keratitis.

## 5. Anti-Inflammatory Activity of Thymol in Drug-Induced Inflammation

Inflammation is a necessary biological activity as a defense of the organism against dangerous stimuli including infections, damaged cells, and toxic compounds. Acute inflammation is essential for recovery and repair, but persistent inflammation can cause a number of illnesses. The morbidity and mortality of a wide range of chronic diseases, such as diabetes mellitus, cancer, autoimmune diseases, non-alcoholic fatty liver disease, cardiovascular disease, chronic kidney disease, arthritis, and neurodegenerative and behavioral health disorders, are directly linked to inflammation. Inflammatory cells which are activated by infectious and non-infectious stimuli, as well as cell injury, trigger inflammatory signaling pathways, most frequently the NF-*κ*B, MAPK, and JAK-STAT pathways (Figure 2 and Figure 3) [96,97,98].

Although chemotherapy drugs are important in overcoming some cancer diseases, they have also harmful side effects, many times associated with an unbalance of ROS and an inflammatory process in some organs. Finding ways to mitigate these effects is essential, and thymol has been the target of study, as can be read below.

Ototoxicity, gastrotoxicity, myelosuppression, and allergic responses are observed in cisplatin therapeutics to treat a variety of solid organ malignancies. Nevertheless, cisplatin’s primary dose-limiting adverse effect is nephrotoxicity, which restricts its application as a chemotherapeutic drug. Studies have suggested that inflammation plays an important role in cisplatin-induced renal injury. Taking into account this fact, El-Sayed et al. [99] evaluated the possible protective effect of thymol and carvacrol against cisplatin-induced nephrotoxicity, using an animal model by injecting intraperitoneally (i.p.) in male rats, a single dose of cisplatin (6 mg/kg) and diverse parameters (serum urea, creatinine, TNF-α, MDA, caspase-3, SOD and catalase activity) were followed and compared to a control without the administration of cisplatin. Administration of thymol (20 mg/kg/day) for 14 days before cisplatin and for 7 days after cisplatin significantly decreased the levels of urea, creatinine, and TNF-α in serum by 50, 63, and 21%, respectively (Table 4), in comparison with the cisplatin-treated group. Thymol also decreased the caspase-3 (43%) and MDA content (32%), and restored reduced glutathione (GSH), SOD, and catalase levels (78, 58, and 246%, respectively) in comparison with the cisplatin-treated group. Compared to this group, the animals treated with thymol (20 mg/kg/day) and carvacrol (15 mg/kg/day) for 14 days prior to cisplatin and 7 days following cisplatin showed a significant decrease in elevated serum levels of urea, creatinine, and TNF-α by 60, 80, and 34%, respectively, that is, the combined isomers showed synergistic nephroprotective effect. These findings enabled the authors to propose that thymol and carvacrol, or their combinations, may be viable options for preventing nephrotoxicity caused by cisplatin through their anti-inflammatory, anti-apoptotic, and antioxidant properties [99].

Taking the same approach, that team has now conducted a study with another drug, doxorubicin, a cytotoxic anthracycline antibiotic used in the treatment of diverse types of cancer that induces cardiotoxicity [100]. A single dose of 10 mg/kg of doxorubicin injected intravenously in rats increased serum levels of lactate dehydrogenase (LDH), aspartate aminotransferase (AST), creatine phosphokinase (CPK), creatine kinase isoenzyme-MB (CK-MB), TNF-α, and cardiac troponin-I cTn-I after 2 days of treatment as compared with the control group. In addition, doxorubicin produced a significant increase in the heart contents of caspase-3 and MDA, and a decrease in GSH cardiac levels and the enzymatic antioxidant activities of SOD and catalase as compared with the control group. However, administration of thymol (20 mg/kg/day) for 14 days before doxorubicin and for 2 days after doxorubicin reversed those results, that is, significantly decreased the levels of LDH, AST, CPK, CK-MB, cTn-I, and TNF-α (Table 4) in serum by 31%, 53%, 31%, 35%, 51%, and 40%, respectively; and reduced the cardiac contents of caspase-3 (27%) and MDA (45%) and increased GSH content (35%) as well as the cardiac activity of SOD and catalase (57% and 33%, respectively) in comparison with the doxorubicin-treated group [100]. Carvacrol (25 mg/kg) induced a similar effect to that of thymol and a combination of both had a synergistic protective effect against doxorubicin-induced cardiotoxicity in rats [100]. As aforementioned for cisplatin, the authors [100] believed that the protective effect of thymol might be attributed to the antioxidant, anti-apoptotic, and anti-inflammatory activities.

Gentamicin is an aminoglycoside antibiotic frequently used to treat severe infections brought on by aerobic Gram-negative bacteria; nevertheless, after a single dose, administration can cause nephrotoxicity, restricting its usefulness [101]. These authors used rats that received thymol 20 mg/kg/day, per os, for 15 days, and gentamicin (80 mg/kg/day, i.p.) starting from the 8th day. In the group to which only gentamicin was administered, there was an increase of creatinine, MDA, NO, TNF-α, IL-18, Bax, and caspases-3 and 9 when compared to the control without any treatment, nevertheless, the administration of thymol lowered serum creatinine as well as all of the other parameters. Thymol also reduced renal NF-*κ*B p65 and kidney injury molecule-1 (KIM-1) expressions (Table 4). KIM-1 is a transmembrane glycoprotein expressed by proximal tubular cells in response to damaging kidney stimuli. It may play a role in clearing debris from damaged renal cells [101]. These findings made it possible to conclude that thymol’s anti-inflammatory, anti-apoptotic, and antioxidant properties are responsible for its nephroprotective effects.

A clinically successful chemotherapeutic medication used to treat some cancer types is bleomycin. However, repeated bleomycin administration is associated, in a dose-and time-dependent way, with the development of lung inflammation, which can eventually lead to pulmonary fibrosis in its advanced stages. Although corticosteroids and immunosuppressive drugs are commonly used to treat pulmonary fibrosis, they are not specifically designed for this condition and have significant adverse effects [102].

About 22 nucleotides make up the single-stranded, non-protein-coding RNA species known as microRNAs (miRs). Following complementary binding to the 5′ or 3′-untranslated regions, miRs control the expression of their target mRNAs [102]. For example, miR-29 was discovered to be a master fibro-miR that controls the fibrosis process in the heart, lungs, and liver. Decreased levels of miR-29 are associated with fibrosis in these organs [102].

Differentiation, angiogenesis, cell proliferation, and cell death are influenced by the phosphatidylinositol-3-kinase (PI3K)/Akt signaling. Fibrogenesis is significantly influenced by the PI3K/Akt pathway. Bleomycin-induced lung fibrosis was linked to PI3K/Akt signaling. It seems that the PI3K/Akt pathway can be activated through the downregulation of miR-29 expression by transforming growth factor-β (TGF-β), which subsequently leads to collagen deposition in human lung fibroblasts [102]. Thus, Hussein et al. [102] investigated thymol’s capacity to prevent bleomycin-induced lung fibrosis in mice by modulating miR-29a/TGF-β expression and PI3K/phospho-Akt signaling and avoiding oxidative stress and inflammation in lung fibrosis. The antioxidant activity was evaluated through the determination of MDA and GSH levels and SOD activity. The anti-inflammatory activity was followed through the determination of TNF-α, IL-1β, IL-6, and NF-kB concentrations. The results showed that the bleomycin-treated mice group’s expression of miR-29a was down-regulated, 0.07 times lower than that of the normal group. Compared to the bleomycin group, the groups treated with bleomycin + thymol (50 mg/kg and 100 mg/kg) displayed higher levels of miR-29a expression by 3.32 and 5.08 folds, respectively. However, compared to the control (without any treatment type) or thymol groups, the up-regulation of miR-29a was substantially lowered. When compared to the control group, the bleomycin group’s TGF-β level was higher (181.7%). However, compared to the bleomycin-treated mice, the TGF-β levels were lower in the bleomycin + thymol (50 mg/kg and 100 mg/kg) groups by 50.39% and 54.91%, respectively (Table 4). PI3K and phospho-Akt protein expression were significantly higher in the bleomycin group than in the control group, increasing by 6.1 and 4.3 times, respectively. However, in comparison to the bleomycin group, the bleomycin + thymol (50 mg/kg) group exhibited considerably lower levels of phospho-Akt and PI3K expression (Table 4) by 53.8% and 53.4%, respectively. The same effect was observed when the assay used thymol 100 mg/kg. Pinosylvin, a stilbene, was also able to inhibit the PI3K/Akt pathway in vivo studies, at 30 mg/kg [103].

In what concerns the antioxidant activity, the authors [102] found that bleomycin-induced an increase of MDA (194.9%) in mice in comparison to the control without any treatment, while GSH level and SOD activity dropped by 56.8% and 63.7%, respectively. When compared to animals treated with bleomycin, the MDA levels in the bleomycin + thymol (50 mg/kg and 100 mg/kg) groups were reduced by 40.7% and 55.9%, respectively. Moreover, the bleomycin + thymol (50 mg/kg and 100 mg/kg) groups showed increases in GSH levels of 89.9% and 106.8% and SOD activity of 101.2% and 125.6%, respectively, in comparison to the bleomycin group (Table 4). Since the amounts of TNF-α, IL-1β, IL-6, and NF-kB were elevated by 353.3%, 117.7%, 271.9%, and 191.6%, respectively, compared to control mice, the bleomycin group had severe inflammation. However, compared to the bleomycin group, the TNF-α levels in the bleomycin + thymol (50 mg/kg and 100 mg/kg) groups were significantly reduced by 59.4% and 68%, respectively. Comparing the bleomycin + thymol (50 mg/kg and 100 mg/kg) groups to bleomycin-treated mice, the IL-1β levels were reduced by 34.8% and 41.8%, respectively, and the IL-6 level by 52.8% and 55.5%, respectively. In comparison to bleomycin, the bleomycin + thymol (50 mg/kg and 100 mg/kg) groups had lower NF-kB concentrations by 45.9% and 51.9%, respectively. In this context, the authors [102] consider that further studies must be developed to better understand the molecular mechanism of the anti-fibrotic activity of thymol.

5-Fluorouracil (5-FU) is used to treat a variety of cancers, such as ovarian, head and neck, gastrointestinal tract, and breast malignancies. 5-FU inhibits the thymidylate synthase, which stops the growth of cancer cells, however, 5-FU inevitably affects normal cells, particularly intestinal mucosal cells and other cells with high rates of proliferation [104]. Oxidative stress and inflammatory markers were followed in rats’ intestines submitted to 5-FU (150 mg/kg, i.p.) with previous treatment with thymol (60 or 120 mg/kg). The results were compared to the rat’s control without any treatment and with those only treated with 5-FU. Severe intestinal damages were evidenced by histopathological changes in animals treated with 5-FU along with oxidative and inflammatory responses. GSH, SOD, and GPX levels were significantly reduced by 5-FU, reaching 66%, 81%, and 67% of the control group’s levels, respectively. Furthermore, compared to the control group, 5-FU significantly increased TBARS levels by almost four times. However, thymol administration at a dose of 60 mg/kg normalized the SOD level to match that of the control group. Remarkably, it was discovered that administering 60 mg of thymol increased the deficient antioxidant GSH, by almost 150% compared to the control group. Rats treated with 5-FU and 120 mg/kg of thymol demonstrated better protection against the depletion of antioxidants caused by 5-FU because normalized SOD and GPX to levels similar to the control group and increased GSH to roughly 2.5 times the control group’s levels. Concurrent administration of either dose of thymol (60 or 120 mg/kg) significantly reduced lipid peroxidation in terms of TBARS content levels to 43% and 23%, respectively. A dose-dependent antioxidant activity of thymol was observed since as compared to the dose of 60 mg/kg, the levels of GSH, GPX, and TBARS significantly improved with a dose of 120 mg/kg [104]. 5-FU raised the level of NF-*κ*B protein by almost 2.5 times in comparison to the control group. Thymol treatment at 120 mg/kg, but not 60 mg/kg, reduced 5-FU’s capacity to promote NF-*κ*B expression to a level equivalent to that of the control group. 5-FU significantly increased intestinal TNF-α protein level expression by almost 19 times when compared to the control group. In comparison to the control rats, the results showed that 5-FU significantly increased the levels of PGE2, COX-2, and IL-6 proteins by 2.4, 1.7, and 3.2 times, respectively, whereas thymol at a dose of 120 mg/kg kept COX-2 at a level similar to the control group. While 5-FU did not significantly reduce the expression of anti-inflammatory IL-10, concurrent administration of thymol (120 mg/kg) slightly increased the anti-inflammatory IL-10 level to roughly 1.3 times that of the control and 5-FU-treated groups [88]. The authors also found that thymol suppressed the expression of p38 and phosphorylated c-Jun N-terminal kinases (p-JNK), mitogen-activated protein kinase proteins and significantly inhibited the 5-FU-induced expression of NF-*κ*B, and TGF-β1. According to these observations, the authors consider that thymol has a promising protective effect against intestinal mucositis caused by 5-FU by suppressing TGF-β/p38/p-JNK signaling and inhibiting oxidative and inflammatory pathways [104].

Badr et al. [105] studied the pathophysiological mechanism of 5-FU on intestinal mucositis, as well as its immunomodulatory action and the molecular mechanism of the immunomodulatory action of thymol against 5-FU-induced intestinal mucositis through the IL-17/Notch1/signal transducers and activators of transcription (STAT)3 signaling pathway. The assay was similar to that previously performed [105] but now focused on other mechanisms. The Notch signaling pathway has been found to regulate epithelial cell homeostasis, which is crucial for epithelial integrity because it regulates the balance between secretory and absorptive cell lineages. Blocking the Notch signaling system may be a possible treatment for intestinal inflammation since it controls the differentiation and activation of distinct immune cell types. Notch activation has a pro-inflammatory effect. IL-17 is a main pro-inflammatory cytokine produced mostly by CD4 T-helper 17 (Th17) cells in addition to other cells, including macrophages, dendritic cells, and natural killer cells. Furthermore, IL-17-Notch1 crosstalk increases Th17-induced Notch1 target genes, which are involved in cell proliferation and inflammation. It has been established that the STAT3 pathway is connected to the development and control of Th17 cells and macrophages, therefore, STAT3 may be an essential mediator in the regulation of inflammation and immune cell growth. The intestinal level of IL-17 increased by roughly 67.6% in the rats treated with 5-FU compared to the control group, but thymol 120 mg/kg reduced the level of interleukin by 25.55% compared to that group only treated with 5-FU. Compared to the control group, the 5-FU group’s intestinal Notch1 expression was significantly upregulated by 3.3 times. In contrast, the thymol 120 mg/kg group’s intestinal Notch1 expression was downregulated by 65.5%. In addition, compared to the thymol 60 mg/kg group, the thymol 120 mg/kg showed a significant downregulation of intestinal Notch1 expression by 66.6% (Table 4). The injection of 5-FU induced a marked activation of intestinal phospho-STAT3, and the intestinal phospho-STAT3/total-STAT3 ratio was significantly elevated by 16.7%, in comparison to the control group. However, administration of thymol in doses of 60 mg and 120 mg showed a marked increase in the intestinal phospho-STAT3/total-STAT3 ratio of 84.2% and 93.9% (Table 4), respectively, when compared with 5-FU intoxicated rats. These results disagree with those previously reported in which thymol and carvacrol were able to decrease STAT3 in the stimulated macrophage cells [106].

Thymol (30 and 100 mg/kg) exhibits gastroprotective properties suppressing the development of acute and chronic ulcers brought on by various substances (ethanol; indomethacin, a non-selective inhibitor of COX; and acetic acid), decreasing the inflammatory process (inflammatory cell infiltration and edema), and elevating the production of mucus in rats. Moreover, PGE_2_ and K_ATP_ channels were involved in the gastroprotective effect of thymol on ethanol-induced ulcers in rats. Thymol’s cytoprotective action and K_ATP_ channel activation in the indomethacin-induced gastric ulcer model can both be explained by PGE2’s role in the antiulcerogenic impact of that phenolic monoterpene [107]. According to these authors, their data suggest that mucus production (Table 4) is involved in the gastroprotective effect of thymol, which may be due to the stimulation of PGE_2_ production.

Beyond the gastrotoxicity of indomethacin, Geyikoglu et al. [108] also reported its hepatotoxicity, for this reason, they studied the hepatoprotective role of thymol in an indomethacin-induced gastric ulcer rat model. Sprague-Dawley rats were used in the study. Indomethacin (30 mg/kg) was used as a gastric ulcer inducer, and thymol (75, 100, 250, and 500 mg/kg) was orally administered 10 min after the administration of indomethacin. Ranitidine was used as positive control, at 50 mg/kg. The results observed were: indomethacin significantly increased the levels of hepatic enzymes AST, alanine transaminase (ALT), and LDH, TNF-α and endothelial nitric oxide synthase (eNOS), and caspase-3 activation, while decreased PGE_2_ levels. Thymol, particularly at 250 mg/kg shifted this behavior, inducing a significant improvement of those parameters (Table 4) [108]. In conclusion, thymol may protect the stomach and liver from the side effects induced by indomethacin.

Thymol can also be used to produce prodrugs to reduce the side effects of the drug and improve the pharmacological activity. Ashraf et al. [109] produced 4 prodrugs using the propionic acid derivative flurbiprofen, a nonsteroidal anti-inflammatory drug in the class of COX inhibitors, and vanillin, thymol, umbelliferone, and sesamol. The synthesis of these prodrugs could decrease the side effects of flurbiprofen that can cause gastrointestinal problems and modify the platelets’ function. All the synthesized prodrugs reduced the inflammation of the paw edema at 4 h of study of adult healthy mice of either sex weighing 20–30 g, induced by carrageenan and egg albumin. All flurbiprofen prodrugs presented anti-inflammatory activity comparable to the standard flurbiprofen. The in vitro hydrolysis of the prodrugs in the simulated gastric fluid (pH = 1.2) was null, whereas in the simulated intestinal fluid (pH = 7.4) and 80% human plasma (pH = 7.4) the regeneration of flurbiprofen was found to be from 61% to 92%. Such results may hypothesize that the prodrugs synthesized by the authors [109] might be less irritating for the gastrointestinal tract than the active drug.

Imidacloprid is a systemic neonicotinoid pesticide used to control insects, to kill fleas, flies, and lice on pets and livestock. Exposure to imidacloprid has been associated with a number of health issues, such as metabolic, neurotoxic, hepatotoxic, nephrotoxic, mutagenic, teratogenic, genotoxic, and immunotoxic effects [110]. In a 56-day oral study, Abdelgawad et al. [110] evaluated if thymol 30 mg/kg could mitigate the adverse effects of imidacloprid 22.5 mg/kg on the rat liver. The authors [110] found that thymol dramatically reduced the rise in hepatic enzyme leakage, hepatic oxidative stress, lipid peroxidation, DNA damage, and inflammation by reduction of NO and inflammatory cell infiltrations (portal and intralobular) (Table 4) linked to the neonicotinoid pesticide. Furthermore, thymol treatment significantly reduced caspase-3 in the hepatocytes of the thymol + imidacloprid-treated group compared to the imidacloprid-treated group but did not affect NF-kB p65 immunoexpression.

The possible beneficial effect of thymol 30 mg/kg on the neurotoxic effects induced by imidacloprid 22.5 mg/kg was also evaluated [111] in the brains of male Sprague-Dawley rats. The administration protocol was similar to that previously reported [110]. As reported for the liver, in the rat brain, thymol reduced the release of inflammatory elements such as NO and MPO (Table 4), and downregulated caspase-3 resulting from imidacloprid exposure, but did not alter the imidacloprid-induced nuclear factor (NF-*κ*B p65) increase [111].

**Table 4:** Anti-inflammatory activity of thymol induced by drugs.

Drug/Side Effect	Treatment	Results	Reference
Cisplatin 6 mg/kg (single dose) intraperitoneally (i.p.) to male rats/nefrotoxicity	Thymol 20 mg/kg/day for 14 days prior to cisplatin and 7 days following cisplatin	Urea↓, creatinine↓, TNF-α↓, caspase-3↓ (43%) and MDA↓, (GSH)↑, SOD↑, catalase↑	[99]
Doxorubicin 10 mg/kg (single dose) intravenously to rats/cardiotoxicity	Thymol 20 mg/kg/day for 14 days before doxorubicin and for 2 days after doxorubicin	Lactate dehydrogenase (LDH)↓, aspartate aminotransferase (AST)↓, creatine phosphokinase (CPK)↓, creatine kinase isoenzyme-MB (CK-MB)↓, cardiac troponin-I (cTn-I)↓, TNF-α↓	[100]
Gentamicin 80 mg/kg/day i.p. starting from the 8th day to rats/nefrotoxicity	Thymol 20 mg/kg/day, p.o., for 15 days	Creatinine↓, MDA↓, NO↓, TNF-α↓, IL-18↓, Bax↓, caspases-3↓ and 9↓, renal NF-*κ*B p65↓, kidney injury molecule-1 (KIM-1) expressions↓	[101]
Bleomycin 15 mg/kg i.p., twice a week for 4 weeks to mice/lung fibrosis	Thymol 50 and 100 mg/kg p.o., for 4 weeks	MDA↓, GSH↑, SOD↑, TNF-α↓, IL-1β↓, IL-6↓, NF-kB↓, TGF-β↓, phospho-Akt↓ and PI3K↓ expression	[102]
5-Fluorouracil (FU) 150 mg/kg on 6th and 7th days, i.p. to rats,/severe intestinal damages	Thymol 60 but particularly 120 mg/kg, p.o., for 11 days	GSH↑, SOD↑, glutathione peroxidase (GPx)↑, NF-*κ*B expression↓, TNF-α protein level expression↓, NF-*κ*B↓, TGF-β1↓, PGE2↓, COX-2↓, IL-6↓, IL-10↑, expression of p38↓, phosphorylated c-Jun N-terminal kinases (p-JNK)↓	[104]
5-Fluorouracil (FU) 150 mg/kg on 6th and 7th days, i.p. to rats/intestinal mucositis	Thymol 60 but particularly 120 mg/kg, p.o., for 11 days	IL-17↓, intestinal Notch1 expression↓, intestinal phospho-STAT3/total-STAT3 ratio↑	[105]
Absolute ethanol 4 mL/kg p.o. 45 min after treatment/gastric ulcer	Thymol 30 mg/kg, p.o.	Total lesion area↓, amount of mucus↑,	[107]
Indomethacin 30 mg/kg p.o., to rats/hepatotoxicity	Thymol 250 mg/kg p.o. 10 min after the administration of indomethacin	AST↓, ALT↓, LDH↓, TNF-α↓, endothelial nitric oxide synthase (eNOS)↓, and caspase-3 activation↓, decreased PGE_2_↓	[108]
Imidacloprid (pesticide) 22.5 mg/kg to rat/hepatotoxicity and brain disorder	Thymol 30 mg/kg	Hepatic enzyme leakage↓, hepatic oxidative stress↓, lipid peroxidation↓, DNA damage↓, NO↓ inflammatory cell infiltrations (portal and intralobular)↓ caspase-3↓, myeloperoxidase (MPO)↓ in brain	[110,111]

↓, decrease; ↑, increase.

## 6. Anti-Inflammatory Activity of Thymol in Livestock

The production of livestock to provide the food trade requires quality according to international standards. Extensive animal production, sometimes in artificial and claustrophobic conditions along with a diet rich in antibiotics and steroids, leads to animal stress and undesirable inflammatory patterns. Moreover, this overload of additives has harmful effects on the environment that affect human and animal health and well-being. For this reason, it is urgent to find more environmentally friendly solutions and there have been several approaches to achieve this objective, one of which is to use volatile compounds of plant origin, such as thymol.

**Figure 8:**
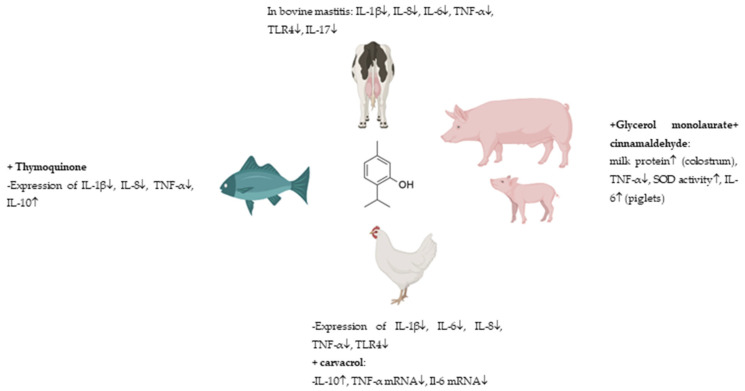
Anti-inflammatory activity of thymol in livestock (created in BioRender (https://www.biorender.com/, accesed on 2 May 2025). IL-1β, interleukin-1β; IL-6, interleukin-6; SOD, superoxide dismutase; TLR4, toll-like receptor-4; TNF-α, tumor necrosis factor-alpha; ↓, decrease; ↑, increase.

The swine industry’s economic benefits and production efficiency are directly correlated with sow reproductive performance. Sows frequently experience exhaustion, anorexia, constipation, prolonged labor, weight loss, and other symptoms during lactation due to the significant changes in hormones and metabolism in the body, particularly in the late gestation and early lactation periods. Antibiotic additives significantly reduce the stress associated with piglet weaning and sow delivery while also enhancing animal resistance to illness. Nonetheless, antibiotic misuse poses a risk to human health because of increased bacterial resistance, elevated antibiotic levels in cattle and poultry, and environmental contamination [112]. Glycerol monolaurate (C_15_H_30_O_4_) has multiple beneficial biological properties, such as antibiotics with multiple cellular targets, which hampers the development of resistance of microorganisms to it, as happens with antibiotics [112]. By improving animal immunity, antioxidant balance, and intestinal microbiota health, glycerol monolaurate can increase the feed conversion ratio (FCR), meat quality, intestinal microbiota, and barrier function of broilers. It can also decrease the amount of methanobacteria and protozoa in sheep rumen fluid, stabilize intestinal microbiota, and improve the growth performance of weaned piglets. Furthermore, glycerol monolaurate is frequently utilized in broilers to enhance growth performance and preserve the intestinal microecological balance and in weaned piglets to lessen diarrhea and boost good gut bacteria [112]. Li et al. [112] aimed to examine the effects of supplementing with maternal glycerol monolaurate complex (glycerol monolaurate 800 g/kg + cinnamaldehyde 54 g/kg + thymol 6 g/kg) and antibiotics (acetylisovaleryltylosin tartrate) during late gestation and lactation on the growth and reproductive performance of piglets and sows. Li et al. [112] observed that the glycerol monolaurate complex during lactation tended to lower the sows’ TNF-α and raise the amount of milk protein in the colostrum. In addition, 0.2% glycerol monolaurate complex reduced the level of TNF-α in sows and weaned piglets and raised the levels of IL-6 and serum total T-SOD activity (Figure 8) in weaned piglets. Moreover, the glycerol monolaurate complex tended to increase the microbiological diversity of sows and piglets, while acetylisovaleryltylosin tartrate decreased the microbial diversity of sows. Although Li et al. [112] emphasize the benefits of glycerol monolaurate, we do not want to fail to highlight that the activity may be a joint contribution of glycerol monolaurate and two volatiles, cinnamaldehyde, and thymol, that constitute the complex.

In order to determine the best supplementation amount of the mixture of carvacrol + thymol (1:1) based on linear and quadratic regression analysis, Li et al. [113] assessed the effects of dietary supplementation with varying dosages of carvacrol + thymol (30, 60 or 120 mg/kg) on the growth performance, intestinal digestive and absorptive capacities, and intestinal health, and inflammation in broiler chickens. At a dose of 30 mg/kg of carvacrol + thymol, the mRNA abundance of ileal inflammatory IL-6 showed a quadratic decrease at 42 days. An ileal anti-inflammatory IL-10 (42 days) showed a linear or quadratic increase with increasing dietary carvacrol + thymol supplementation dosage. Compared with the other three groups (0, 60, and 120 mg/kg), carvacrol + thymol supplemented at 30 mg/kg significantly decreased ileal TNF-α mRNA expression (Figure 8) in 21 days [113]. Li et al. [113] concluded that animals fed diets containing 30 mg/kg carvacrol + thymol showed improved intestinal morphology, function, and growth performance when compared to the other three groups (0, 60, and 120 mg/kg), therefore, 30 mg/kg of carvacrol + thymol supplementation was the ideal dosage for broiler diets.

Intestinal villus and enteroid culture were isolated from the small intestines (jejunum and ileum) of chicken to serve as models for searching gut functions [114]. These authors aimed to study the protective effects of thymol (0.07 µM), thyme essential oil with 50% of thymol (20 ppm), phenols-rich grape seed extract (100 ppm), capsaicinoids 10% (10 ppm), ginger essential oil (100 ppm), and tea tree oil (100 ppm) on mitigating oxidative stress and LPS-induced inflammation in chicken apical-out enteroids. Gene expression of selected cytokines and tight-junction proteins was conducted after 6 h of LPS challenge. There was a significantly higher expression of proinflammatory cytokines in LPS-induced cytokine expression in comparison to control without the presence of LPS. Among the extract treatments, thymol, thyme essential oil, and phenols-rich grape seed extract showed the largest decreases in the LPS-induced cytokine expression when compared to the LPS-induced group without extract treatments. Thymol and tea tree oil treatments nearly reversed the negative effects of LPS on *ZO1* (zonula occludens-1) and *OCCL* (occludin) expression when compared to the LPS group; capsaicinoids treatment upregulated *ZO1* but not *OCCL* expression, while phenols-rich grape seed extract treatment upregulated only *OCCL* expression; ginger essential oil and tea tree oil groups showed no difference compared with the LPS group [114].

Among the plant-based feed additives used in aquaculture are thymol and thymoquinone. They have been used as dietary supplements in fish nutrition due to their potential benefits and therapeutic effects on meat quality, antioxidant capacity, immunity, nutrient absorption, and growth performance [115]. The impact of thymol and thymoquinone on the transcription of genes encoding digestive enzymes and antioxidant potential in Nile tilapia (*Oreochromis niloticus*) has not been extensively examined. The impact of thymol and/or thymoquinone in vivo on Nile tilapia growth performance, antioxidant and digestive enzymes, cytokines, and the expression of genes linked to autophagy, as well as *Aeromonas sobria* resistance in Nile tilapia was evaluated for the first time by Ibrahim et al. [115]. Fish infected with *A. sobria* die from ulcer development, ascites, and hemorrhagic septicemia [115]. In what concerns the anti-inflammatory activity, the authors verified that compared to the control group, the transcriptional levels of the *Il-1β*, *tnf-α*, and *Il-8* genes were probably considerably lower after supplementing thymol and thymoquinone together (mixture of 1:1 with a final concentration of 200 mg/kg diet), then separately (200 mg/kg diet for thymol and 200 mg/kg diet for thymoquinone). In the meantime, the thymol/thymoquinone-supplemented group showed the highest significant expression level of the *Il-10* gene. In addition, the study also showed that the use of thymol/thymoquinone in fish diets led to increased growth rates, enhanced immunological responses, supported the expression of digestive and antioxidant genes, and offered protection against *A. sorbia* infections [115].

Using network pharmacology and molecular docking techniques, Cao et al. [95] studied the possible targets, and the mechanisms of action of the active isolated volatiles, as well as of those constituents of oregano essential oil in the treatment of bovine mastitis disease. Oregano essential oil was mostly composed of *p*-cymene, carvacrol, and thymol. Cytoscape (https://cytoscape.org/, accesed on 2 May 2025) analysis identified TNF, TLR4, albumin, IL-1, TLR2, IL-6, IFNG (interferon-γ), and MyD88 as the primary targets of those constituents. The anti-inflammatory effect of thymol, *p*-cymene, and carvacrol is due to their abilities to bind to proteins that correspond to targets such as TNF, TLR4, albumin, IL-1β, TLR2, IL-6, IFNG, and MyD88. It can also regulate the PI3K-Akt, MAPK, IL-17, and NF-B signaling pathways. As a result, bovine mastitis may benefit from the use of oregano essential oil after this theoretical approach [95].

## 7. Encapsulated Thymol in Micro- and Nanoparticles

The biological properties of thymol, particularly the anti-inflammatory activity, have been demonstrated through diverse in vitro and in vivo methods as aforementioned. However, its physicochemical characteristics may hamper its application, because thymol is hydrophobic, volatile, and not stable under oxygen or light action. In this context, it has been developed ways to improve stability and decrease volatility using encapsulation methods. Some of these works are focused on treating skin wounds or inflammations, as may be read below. For example, Pivetta et al. [116] encapsulated thymol in nanostructured lipid carriers composed of natural lipids (illipe butter and calendula oil) and produced by the sonication method. These nanostructured lipid carriers were then incorporated into a gel and tested in vivo on two different mouse models of skin inflammation (croton oil-induced acute inflammation model and anthralin-induced ear edema model), showing anti-inflammatory activity, better than free thymol. The same formulation was tested in an imiquimod-induced psoriasis mouse model and showed improved healing, compared to the negative control (carbopol gel), but less activity than the positive control (clobetasol propionate) [116]. The authors claim that encapsulating thymol in natural nanostructured lipid carriers can be used as a topical delivery method for inflammatory skin conditions.

Another approach to delivering drugs, and in this case thymol, is through the utilization of polycaprolactone (biocompatible and bioresorbable polymer that the FDA approved for use in several medical and drug delivery devices) electrospun nanofibrous patch loaded with thymol [117]. García-Salinas et al. [117] found that this patch shows anti-inflammatory activity in J774 macrophages because they observed a down-regulation of pro-inflammatory genes related to the NF-*κ*B pathway, even better than patch containing tyrosol (other compound tested by the authors). In addition, the thymol patch showed a low affinity for cell attachment, which facilitated dressing removal and prevented wound surface damage while changing dressings [117]. These findings suggest that thymol-loaded patches may be viable options for creating dressings with anti-inflammatory properties.

Surface-functionalized poly(lactic-co-glycolic acid) nanoparticles using different surface functionalization strategies and loaded with thymol were developed for topical administration for treating acne infection (antimicrobial activity against *Cutibacterium acnes*) and associated with inflammation. For surface functionalization, the following compounds were used: phosphatidylcholine (TH-NP-L-), Poloxamer 188 (TH-NP-P-), or Poloxamer 407 (TH-NP-PP-), for negatively charged particles. Positively charged particles were also produced containing chitosan (TH-NP-P-C+, THNP-PP-C+), where the aqueous solution contained 1% acetic acid. These nanoparticles were obtained by solvent evaporation method [118]. The anti-inflammatory activity was performed in two models: the TNF-α-induced human keratinocyte cells (HaCat) inflammation model; and the *C. acnes*-induced inflammation model [118]. Wound healing activity was also made by the scratch assay and using HaCaT cells. Free thymol was able to reduce TNF-α-induced human keratinocyte cells (HaCat) secretion of IL-6 as well as all surface functionalized nanoparticles. With the exception of TH-NP-PP-, the remaining nanoparticles had higher anti-inflammatory activity than the free thymol. The most active were the positively charged nanoparticles. In *C. acnes*-induced inflammation model, there was an increase in the expression of genes encoding the inflammatory cytokines TNF-α, IL-1β, IL-1 α, IL-6, and IL-8, while free thymol and the surface functionalized nanoparticles were able to reduce such expression, but with different strength [118]. Regarding wound healing surface-functionalized nanoparticles had better activity than free thymol. Such results may indicate that surface-functionalized poly(lactic-co-glycolic acid) nanoparticles loaded with thymol may be viable to be used for treating acne associated with inflammation [118].

Thymol-loaded alginate microparticles were prepared by electrospraying method and incorporated into the chitosan-gelatin film. This formulation was part of an in vivo study using rats with opened wounds on the dorsal surface by removing the skin with a diameter of 2.5 cm. The biological activities were compared to a negative control group (sterile gauze), and a positive control group (commercial silver-containing dressing) [119]. The histological analysis showed that the chitosan-gelatin/thymol-loaded alginate microparticles significantly enhance epithelialization, and collagen deposition, and induce skin regeneration. Furthermore, on day 7, the chitosan-gelatin/thymol-loaded alginate microparticles and the positive control showed no more mononuclear inflammatory cell population in the wounds, whereas the negative control showed signs of this cell population [119].

The production of nanoparticles loaded with thymol was developed by Sheorain et al. [120] using the ionic complexation method of tragacanth gum and chitosan at different concentrations to obtain a minimum particle size along with higher encapsulation efficiency. The anti-inflammatory activity of thymol-loaded nanoparticles was evaluated through human red blood cell membrane stabilization. The membrane stabilization percentage was 91.07% and 84.11% for diclofenac (0.2 mg/mL) and thymol (0.5 mg/mL), respectively, and 89.60%, 80.69%, 72.96% and 57.80%, at different concentration of thymol loaded chitosan-tragacanth (0.5 mg/mL, 0.25 mg/mL, 0.125 mg/mL, 0.0625 mg/mL, respectively). This assay is not focused on skin diseases as the former ones reported, but it shows the potential of thymol-loaded nanoparticles to be applied in the pharmaceutical industry [120].

Alzheimer’s disease, the predominant form of dementia, is rapidly rising to the top of the list of the most costly and deadly illnesses of the twenty-first century. Inflammatory indicators linked to the onset and clinical progression of Alzheimer’s disease have shown a strong correlation between sleep disturbance and dementia risk. Higher blood levels of pro-inflammatory cytokines and other inflammatory factors have been reported in individuals with this condition and mild cognitive impairment. Microglial macrophages in the brain are activated and promote sustained production of pro-inflammatory cytokines. Therefore, to halt the progression of the condition, anti-inflammatory medication needs to be considered. Safarbalou and Abbasi [121] hypothesized that liposome-encapsulated thymol may prevent Alzheimer’s disease. In this context, the authors [121] assessed the anti-inflammatory effect of liposome-encapsulated thymol administered orally to a rat model in which Alzheimer’s disease was induced. Free thymol (40 and 80 mg/kg) and liposome-encapsulated thymol (40 and 80 mg/kg) were used in rats in which Alzheimer’s disease was induced. The concentrations of IL-1β, IL-6, TNF-α, and COX-2 and brain-derived neurotrophic factor (BDNF) were assessed in serum and hippocampus. The authors found that there was a significant increase in concentrations of IL-1β, IL-6, TNF-α, and COX-2 in the Alzheimer’s group compared with healthy control rats. The results also showed the treatment with free thymol 80 mg/kg and thymol coated with liposomes also decreased serum concentrations of IL-1β, IL-6, TNF-α, and COX-2 and increased BDNF compared with control Alzheimer rats in hippocampus and serum. Moreover, the authors [121] also found significant correlations between serum and hippocampus concentrations of IL-1β, IL-6, TNF-α, and COX-2.

UiO-66 is a metal-organic framework based on zirconium ion (Zr^4+^) that can be used as an anti-cancer, anti-inflammatory, antibacterial, and other drug carrier system. This drug carrier system is due to the fact that it presents high porosity good thermal stability, and a large internal surface area, many times better than other drug carrier systems. Zheng et al. [122] fabricated UiO-66 by solvothermal method to load carvacrol (Car@UiO-66) and thymol (Thy@UiO-66) and evaluated their inhibitory effects on the growth of *Candida albicans*, *Escherichia coli* and *Staphylococcus aureus*. This assay was made using Sprague Dawley rats. UiO-66 was effectively loaded with carvacrol and thymol at loading rates of 79.60% and 79.65%, respectively. After 72 h, the carvacrol and thymol release rates were 77.82% and 76.51%, respectively. Beyond the antimicrobial activities of Car@UiO-66 and Thy@UiO-66 against *Candida albicans*, *Escherichia coli*, and *Staphylococcus aureus*, with minimum bactericidal concentrations (MBC) of 0.313 mg/mL, 0.313 mg/mL, and 1.25 mg/mL, respectively, they also presented anti-inflammatory activity determined in in vivo investigations. Car@UiO-66 and Thy@UiO-66 could lower inflammation, promoting bone formation by suppressing TNF-α and IL6 expression, increasing IL10 expression, and hastening the healing of bone defects in the rats. With these results, the authors [122] conclude that Car@UiO-66 and Thy@Ui O-66 can be attractive candidates for the treatment of oral infectious disorders and the repair of bone deformities due to their unique combination of antibacterial, anti-inflammatory, and osteogenic capabilities.

A lipid-microencapsulated blend of citric (25%) and sorbic (16.7%) acids, thymol (1.7%), and vanillin (1.0%) (AviPlus^®^p, Vetagro S.p.A., Reggio Emilia, Italy) was used to feed broiler chicken. This approach was performed by Johnson et al. [123]. The ileum and jejunum tissues of chickens fed to this diet and those not submitted (control) were subjected to kinome peptide array analysis and the results were compared. The ileum showed a broad rise in the signal transduction pathways centered on activation of NF-*κ*B (factor important on inflammation processes), but also of hypoxia-inducible factor 1 subunit alpha (HIF-1α), 5′ adenosine monophosphate-activated protein kinase (AMPK), mTOR, PI3K-Akt, according to a detailed study of peptides encoding individual kinase target sites. In comparison to control birds, these signaling responses were significantly reduced in the jejunum. The results obtained by Johnson et al. [123], allowed them to conclude that in the ileum, the microencapsulated combination of organic acids and botanicals in which thymol is present, produces improved immune-metabolic responses, while in the jejunum, it produces a more anti-inflammatory phenotype and decreased signaling.

## 8. Synergism Effect of Thymol and Other Natural Products

By targeting several inflammatory targets, the combination of various drugs can result in increased pharmacological action and decreased adverse effects. In this way, there are some research teams, some of them already aforementioned, that have studied the effect of combining thymol with other natural products (silibinin) [124]; nicotine [125]; and carvacrol [125]. For example, the combination of silibinin with thymol (40 μM and 120 μM respectively, with the molar ratio 1:3) had more potent effects on the inhibition of NO, TNF-α, and IL-6, COX-2 activity, mitogen-activated protein kinase (MAPK) and NF-*κ*B than those exerted by individual administration of those compounds in LPS-induced RAW264.7 cells [124]. According to these authors, by inhibiting the NF-*κ*B and MAPK signaling pathways in LPS-induced RAW264.7 cells, silibinin and thymol (1:3) may reduce inflammation.

The synergistic effect of thymol and nicotine in alleviating rheumatoid arthritis was made in rats: thymol (100 mg/kg orally), nicotine (2.5 mg/kg orally), and a combination of nicotine and thymol (half dosages of each given orally). The results obtained showed that the combined therapy was more effective than either medicine alone at improving weight gain and reducing the severity of the condition; produced a more notable decrease in a number of hematological and biochemical rheumatoid arthritis indicators, including rheumatoid factor, C-reactive protein, nitric oxide, MPO, IL-1, and IL-17, but without immunosuppressive adverse effect [126].

In Swiss mice and chicks, Islam et al. [127] aimed to compare the anti-inflammatory properties of thymol with those of the nonsteroidal anti-inflammatory medications celecoxib and ketoprofen. In formalin-induced paw edema in mice, the percentage of reduction paw edema for thymol at 7.5, 15 mg/kg was 18.18%, while for 30 mg/kg, the percentage was 27.27% after 1 h in comparison with the negative control. Thymol 15 mg/kg combined with ketoprofen 42 mg/kg showed a higher percentage of reduction paw edema than all remaining samples [127], after 1 h. In egg albumin-induced paw edema in chicks, thymol at 15 mg/kg had higher percentages of reduction paw edema (31.11%) than ketoprofen 42 mg/kg (26.67%) and thymol 15 mg/kg + celecoxib 42 mg/kg (17.78%). However, the thymol 15 mg/kg + ketoprofen 42 mg/kg significantly increased the percentage of reduction paw edema than their individual groups [127].

## 9. Material and Methods

**Figure 9:**
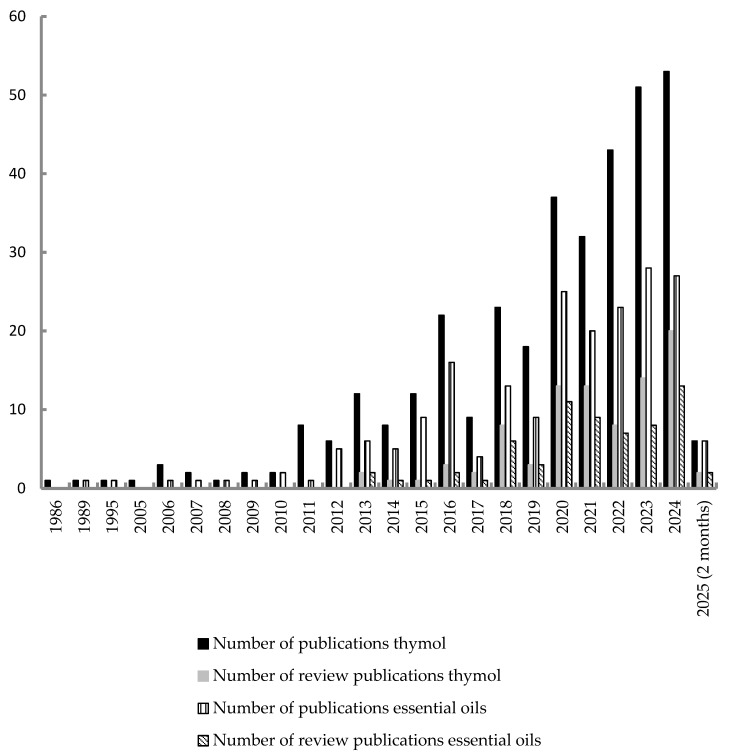
Number of publications per year since 1986.

Only the Web of Science (WOS) Core Collection was used for this review on the anti-inflammatory activity of thymol and EOs or volatiles containing this phenolic compound as a main component. Three hundred fifty-four results were obtained using the term “thymol anti-inflammatory” for the research. Refining the search and searching only for publications in the period from 2014 to the present (28 February 2025), 314 results were obtained, which means that this topic has been arousing the interest of researchers in the last 10 years, particularly from the last 5 years, since in the previous 28 years, only 40 publications could be found in WOS. Since 2014 and until now, the distribution of the publications is represented in Figure 9. The introduction of the term “review” to the “thymol anti-inflammatory” provided 90 results, being the first one from 2013 (Figure 9). This number of results corresponds to 25% of the total number of publications, considering all publications from 1985 (354 publications), and 28% of the total considering the publications from 2014 (314 publications). However, many of these reviews were not specifically about the anti-inflammatory activity of thymol, and those where the anti-inflammatory is reviewed are generally associated with other biological properties [5,32,84,128,129,130,131,132,133,134,135]. Due to the anti-inflammatory activities of thymol along with other biological properties, it has been introduced as a food additive for feeding animals, fish, and poultry [136,137,138,139,140]; on wound healing [30]; and in combination with biopolymers commonly used in skin regeneration for the treatment of chronic wounds [31,141,142] or in other human health applications [143].

Since thymol is a compound in many EOs, a search for “thymol essential oils anti-inflammatory” was done in WOS: 185 of the 205 (1989–2025) publications were identified when the search was restricted to 2014–2025 (February). Up till the first two months of 2025, there were 66 review articles published, with the first two appearing in 2013. The 90 review articles listed above include the 66 review articles on EOs.

The biological properties, including the anti-inflammatory activities, of EOs that have thymol have been extensively revised: *Zataria multiflora* [144,145]; *Adenosma buchneroides* [146]; *Thymus* spp. [147,148,149,150,151]; *Thymus vulgaris* [152,153,154]; *Thymus* spp. [155]; (*Thymus quinquecostatus* [156]; *Thymbra capitata* [157]; *Lippia graveolens* [158]; *Origanum vulgare* [159,160]; *Origanum marjorana* [161].

In summary, this review article focuses on the anti-inflammatory properties of volatiles that contain thymol in greater concentrations than the other constituents, as well as the anti-inflammatory properties of pure thymol in vitro and in vivo in various settings (disease, drug side effects, livestock, encapsulation, and synergism effect with other natural products). In order to give a more comprehensive explanation of some themes this review also cites some other sources in addition to the ones that were found during this search.

## 10. Conclusions and Future Perspectives

This review shows the interest in thymol and essential oils or volatiles where thymol is present, regardless of its amounts, as possessing antioxidant and anti-inflammatory properties, measured through in vitro and in vivo assays. Apart from seeking these attributes, this review revealed that the authors are also interested in possible mechanisms behind the anti-inflammatory action.

Extensive experimental and analytical data discussed throughout this review establish that thymol is a highly promising natural compound with broad-spectrum anti-inflammatory activity, supported by extensive in vitro and in vivo experimental data. Thymol demonstrates the ability to modulate key molecular pathways involved in inflammation, making it an increasingly relevant target for drug development and therapeutic innovation. The anti-inflammatory activity of essential oils containing thymol as the main constituent has also been demonstrated either in vitro or in vivo; however, the results may change because thymol is combined with other essential oil constituents, and some of them may act synergistically or antagonistically. Moreover, the amounts of thymol and all other constituents of essential oils vary from one species to another, even if they are harvested from the same species but at different times or locations. To avoid heterogeneity in response, it is preferable to utilize standardized blending, because combining two or more compounds will be better if a synergistic effect occurs.

Thymol has shown efficacy in inhibiting a range of inflammatory markers, such as tumor necrosis factor-alpha (TNF-α), interleukins IL-1β, IL-6, IL-8, and enzymes such as cyclooxygenase-2 (COX-2). These effects are largely attributed to its capacity to interfere with NF-*κ*B and MAPK signaling pathways, which are central regulators of inflammatory gene expression. Such molecular mechanisms underline thymol’s potential to act in various pathological conditions where chronic or acute inflammation plays a key role.

However, the therapeutic application of thymol is hindered by several physicochemical limitations. Thymol is volatile, hydrophobic, and susceptible to degradation when exposed to light or oxygen, which restricts its stability, bioavailability, and sustained activity in pharmaceutical or biomedical applications. Addressing these limitations, recent advancements in encapsulation technologies have played a pivotal role in enhancing thymol’s utility. Various carriers, such as illipe butter and calendula oil, polycaprolactone, poly(lactic-co-glycolic acid), alginate, of tragacanth gum and chitosan, liposomes, a metal-organic framework based on zirconium ion have been successfully employed to encapsulate thymol, offering improved anti-inflammatory responses.

Beyond encapsulation, synergistic combinations of thymol with other natural compounds, such as silibinin, carvacrol, nicotine, and ketoprofen, have shown additive or synergistic anti-inflammatory effects, often allowing for reduced dosages and enhanced therapeutic outcomes.

In conclusion, thymol is a versatile, naturally occurring compound that, because of its biological characteristics, including its anti-inflammatory activity, has the potential to significantly impact both human and animal health. Clinical validation, effective formulation methods, and synergistic therapy design could turn thymol from a research interest into a commonly used drug. Thymol can also be used in polymeric biomaterials for three-dimensional (3D) printing to be used in medicine. These techniques have already been investigated, with thymol and curcumin utilized in SiO_2_ coatings produced via the sol-gel process on 3D-printed surfaces for individualized orthopedic implants. Other phytochemicals can be included to prevent bacterial infections while developing orthopedic implants [162]. The use of some polymers in scaffolds, orthoses, and prosthetic devices for tissue engineering, regenerative medicine, and rehabilitation for patients with disabling neurological diseases can cause inflammation, which, if excessive, can lead to implant rejection [163,164,165,166]. The addition of phytochemicals, such as thymol, may overcome this issue.

## Figures and Tables

**Table 1:** Plant species containing thymol as main component in their EOs or volatiles.

Clade	Order	Family	Species	Reference
Angiosperms, Eudicots	Apiales	Apiaceae	*Achillea millefolium*	[6]
Angiosperms, Eudicots	Apiales	Apiaceae	*Anethum graveolens*	[6]
Angiosperms, Eudicots	Apiales	Apiaceae	*Ferulago angulata*	[7]
Angiosperms, Eudicots	Lamiales	Lamiaceae	*Ocimum basilicum*	[8]
Angiosperms, Eudicots	Apiales	Apiaceae	*Oliveria decumbens*	[9]
Angiosperms, Eudicots	Lamiales	Lamiaceae	*Origanum floribundum*	[10]
Angiosperms, Eudicots	Lamiales	Lamiaceae	*Origanum heracleoticum*	[11,12]
Angiosperms, Eudicots	Celastrales	Celastraceae	*Salacia pallescens*	[13]
Angiosperms, Eudicots	Lamiales	Lamiaceae	*Thymus atlanticus*	[14]
Angiosperms, Eudicots	Lamiales	Lamiaceae	*Thymus caespitius*	[15]
Angiosperms, Eudicots	Lamiales	Lamiaceae	*Thymus linearis*	[16]
Angiosperms, Eudicots	Lamiales	Lamiaceae	*Thymus sipyleus*	[17]
Angiosperms, Eudicots	Lamiales	Lamiaceae	*Thymus vulgaris*	[18,19,20,21,22]
Angiosperms, Eudicots	Lamiales	Lamiaceae	*Thymus zygis*	[23,24]
Angiosperms, Eudicots	Apiales	Apiaceae	*Trachyspermum ammi*	[6,25,26,27,28]
Angiosperms, Eudicots	Apiales	Apiaceae	*Trachyspermum roxburghianum*	[29]

**Table 2:** Anti-inflammatory activity of essential oils with thymol.

Species (Origin)	Plant Part	% of Thymol and Carvacrol and Main Compound (>5%)	Method	Values	References
*Achillea millefolium* Ilam, Iran *Anethum graveolens* Ilam, Iran *Carum copticum* (=*Trachyspermum ammi*) Ilam, Iran	Aerial parts Seeds Seeds	Thymol 26, carvacrol 10, borneol 16, limonene 15, α-pinene 10 Thymol 20, carvacrol 8, limonene 16, α-pinene 9 Thymol 23, carvacrol 6, sabinene 18, borneol 10	Inhibition of nitric oxide (NO) production in in vitro model of lipopolysaccharide (LPS)-stimulated RAW-264.7 macrophages Inhibition of NO production in in vitro model of LPS-stimulated RAW-264.7 macrophages Inhibition of NO production in in vitro model of LPS-stimulated RAW-264.7 macrophages	Production of nitrites 15.04 µM; LPS (33.98 µM) Production of nitrites 41.04 µM; LPS (33.98 µM) Production of nitrites 26.02 µM; LPS (33.98 µM)	[6]
*Carum copticum* *(=Trachyspermum ammi)* Unknown	Seeds	Thymol 46, carvacrol 3, γ-terpinene 20, *p*-cymene 11, β-pinene 6	Inhibition of NO production in in vitro model of LPS-stimulated RAW-264.7 macrophages	Essential oil: % NO inhibition (80% at 45.0 µg/mL) *p*-cymene: % NO inhibition (48.05% at 45.0 µg/mL) γ-terpinene: % NO inhibition (60.20% at 45.0 µg/mL) β-pinene: % NO inhibition (45.20% at 45.0 µg/mL) thymol: % NO inhibition (85.12% at 45.0 µg/mL)	[25]
*Ferulago angulata* Yasouj, Iran	Aerial parts	Thymol 8, carvacrol 3, spathulenol 7, *trans*-anetole 6, *p*-menth-2-en-1-ol 5, myristicin 5	In vivo anti-inflammatory activity through the croton oil-induced male Swiss mice (20–30 g) ear edema	Both 200 and 100 μL/kg of essential oil produced 66% inhibition of ear edema. Indomethacin (positive control): 82% inhibition at 10 mg/kg)	[7]
*Ocimum basilicum*/Kairouan, Tunisia	Seeds	Solid-liquid Soxhlet extraction using *n*-hexane: Concentration (µg/g): Thymol 33, luteolin 9 2-Methyltetrahydro-furan (MeTHF) Concentration (µg/g): Thymol 128, rosmarinic acid 21, luteolin 9, gallic acid 7, chlorogenic acid 7, ellagic acid 6, circimaritin 6	Inhibition of NO production in LPS-induced murine macrophage RAW 264.7 cell line	At 150 µg/mL, NO production decreased by 60% At 150 µg/mL, NO production decreased by 64%	[8]
*Oliveria decumbens*/Dil village, Iran	Aerial parts	Extraction with ethanol:H_2_O (70:30) and then fractionation: *n*-hexane: thymol 56, carvacrol 38 dichloromethane: thymol 53, carvacrol 41	Oxidative burst assay using luminol-enhanced chemiluminescence technique with blood HBSS++ (Hanks Balanced Salt Solution), serum opsonized zymosan (SOZ) and intracellular reactive oxygen species	Crude extract: *IC_50_ = 21.7 µg/mL *n*-Hexane: IC_50_ = 22.6 µg/mL Dichloromethane: IC_50_ = 15.8 µg/mL Positive control (Ibuprofen): IC_50_ = 11.2 µg/mL Thymol: not active (>100 µg/mL) Carvacrol: not active (>100 µg/mL)	[9]
*Origanum floribundum*/ Lakhdaria, Algeria	Aerial parts at flowering stage (July 2011)	Thymol 34, carvacrol 9, γ-terpinene 20, *p*-cymene 16,	In vitro spectrophotometrically lipoxygenase inhibition	IC_50_: 125.7 µg/mL **NDGA (positive control): IC_50_ = 63.4 µg/mL	[10]
*Origanum heracleoticum* (5 samples) /Agrigento, Italy	Leaves and flowers	Thymol 47–65, carvacrol 3–5, γ-terpinene 13–22, *p*-cymene 4–5, carvacrol methyl ether 3–4	Gene expression analysis of ***NF-*κ*B pathway in Caco-2 cells treated with ****TNF-α	An increase trend of *****IL-1α, IL-6 and IL-8 gene expression was observed when compared to the treatment with TNF-α, without statistical significance It is advisable to read the article, data presented as graphics	[11]
*Origanum heracleoticum* (6 samples)/Calabria, Italy	Not identified plant part used	Thymol 9104–54,459, carvacrol 3311–68,379, *o*-cymene 3304–48,176, γ-terpinene 24–19,133, carvacrol-methyl ether 2658–19,844, β-caryophyllene 1182–16,107, γ-muurolene 1364–10,824 (unknown unities, the compounds were chosen if at least one value is >10,000	Inhibition of NO production in lipopolysaccharide (LPS)-induced murine macrophage RAW 264.7 cell line	IC_50_ = 32.77–170.9 μg/mL. Positive controls (indomethacin and L-NAME): IC_50_ = 58.00 and 45.86, respectively)	[12]
*Salacia pallescens*/Idi-Ayunre, Ibadan, Nigeria	Leaves	Extraction with methanol 50%: Thymol 30, 3-carene 16, *p*-cymene 12, caffeine 8, hexadecanoic acid 6, bicycle[3.1.1]hept-2-ene, 2,6-dimethyl-6-(4-methyl-3-pentenyl) 6, caryophyllene 5	In vitro inhibition of nitrite oxide (NO) production Inhibition of interleukin 6 (IL-6) production in in vitro model of LPS-stimulated RAW-264.7 macrophages	IC_50_ = 49.49 µg/mL IC_50_ = 48.74 µg/mL (positive control, ascorbic acid) Nine-fold reduction in LPS induced IL-6 production in RAW-264.7 macrophages pre-treated with 400 µg/mL of the extract. 1.4-fold reduction in LPS induced IL-6 production in RAW-264.7 macrophages pre-treated with 50 µg/mL of the extract	[13]
Siddhalepa Asamodagam Spirit (water distillate derived from *Trachyspermum roxburghianum* seeds/Pakistan	Seeds	The water distillate was then extracted with ethyl acetate: Thymol 93, carvacrol 1	Heat induced hemolysis principle using human red blood cell stabilization method	IC_50_ = 0.57 mg/mL Positive control (aspirin): IC_50_ = 0.24 mg/mL	[29]
*Thymus atlanticus*/Errachidia region, Morocco	Aerial parts	Thymol 24, carvacrol 23, γ-terpinene 21, *p*-cymene 19	In vivo phenol induced ear edema in Wister albino rats of both sexes weighing 100–120 g	The application of phenol alone has developed in the control group an ear inflammation of 77.59% The topical application of essential oil (1 mg/ear) has reduced the ear edema with a percentage of 19.39%. Indomethacin (positive control) (1 mg/ear) has reduced the ear edema with a percentage of 14.71%	[14]
*Thymus caespitius*/Azores, Portugal	Plant material was collected during the flowering phase	Thymol 34, carvacrol 11, *p*-cymene 12, thymol acetate 8, γ-terpinene 5	NO scavenging activity In vitro spectrophotometrically lipoxygenase inhibition	IC_50_ = 0.3 mg/mL IC_50_ = 0.1 mg/mL	[15]
*Thymus linearis*/Harinagar and Dhanachuli, India	Aerial parts	Thymol 67. carvacrol 3, *p*-cymene 10	In vivo anti-inflammatory activity through the carrageenan-induced paw edema of Swiss albino mice In vivo sub-acute anti-inflammatory activity through the formaldehyde induced arthritis in the right hind paw of Swiss albino mice (formaldehyde solution 1% injected on the first day of the experiment. Samples administered orally every day of the experiment. The results were recorded every day till the end (10 days). For knowing all results it is advisable to read the reference	At 5%: Inhibition % after 4 h and 24 h = 3.83 and 6.38, respectively At 10%: Inhibition % after 4 h and 24 h = 5.19 and 9.52, respectively At 20%: Inhibition % after 4 h and 24 h = 9.73 and 14.6, respectively Positive control (Ibuprofen): At 0.004%: Inhibition % after 4 h and 24 h = 26.07 and 37.18, respectively Volume of inflammation (mm^3^) (5%): day 0, day 5, day 10 = 2.26, 2.47, 2.51 Volume of inflammation (mm^3^) (10%): day 0, day 5, day 10 = 2.21, 2.38, 2.38 Volume of inflammation (mm^3^) (20%): day 0, day 5, day 10 = 2.19, 2.32, 2.26 Volume of inflammation (mm^3^) saline water (control): day 0, day 5, day 10 = 2.13, 2.39, 2.37 Volume of inflammation (mm^3^) positive control (Ibuprofen) (0.001%): day 0, day 5, day 10 = 2.11, 2.19, 2.15	[16]
*Thymus sipyleus* Boiss. subsp. *sipyleus* var. *sipyleus*/Sivas, Turkey	Aerial parts	Thymol 66, carvacrol 3, *p*-cymene 9, γ-terpinene 9	In vitro spectrophotometrically lipoxygenase inhibition	12% inhibition for a concentration of 100 μg/mL. NDGA (positive control) 100% inhibition for a concentration of 100 μg/mL	[17]
*Thymus vulgaris*/Szigetvár city, Hungary	Not identified the part of plant used	Beginning of flowering: Thymol 56, carvacrol 2, *p*-cymene 13, γ-terpinene 15 End of flowering: Thymol 54, carvacrol 3, *p*-cymene 21, γ-terpinene 6	Inhibition of cytokine production through *P. aeruginosa* LPS-activated THP-1 macrophage cells	Inhibition of the production of IL-6, IL-8, IL-1β, TNF-α. Thymol (positive control) had better activity These results are based on the figures’ observation. It is advisable to read the article There is not a decrease of the four examined proinflammatory cytokine IL-6, IL-8, IL-β, TNF-α expression both at the mRNA and protein levels These results are based on the figures’ observation. It is advisable to read the article	[18]
*Thymus vulgaris* (purchased)	Not reported	Thymol 49, carvacrol 3, *p*-cymene 29	Aging-related and pro-inflammatory cytokine gene expression in the liver, hippocampus, cerebellum, and cerebral cortex of mice fed with the thyme EO 250 mg/kg/day (0.2% (*w*/*w*) Aging-related and pro-inflammatory cytokine gene expression in age-accelerated NIH-3T3 cells. Thyme oil concentrations: 30, 60, 120 µg/mL	Lower levels of *p16^INK4A^* in the hippocampus than control; lower expression of *Il-1b* in the liver and cerebellum, and *Il6* in the hippocampus than the control; lower expression of *p65* and *p50* (two transcription factors of NF-*κ*B) than the control Lower levels of *Il6* and *Ccl2* mRNA expression (dose-dependent) It is advisable to read the article since the results are presented under graphics	[19]
*Thymus vulgaris*/Póvoa de Lanhoso, Portugal	Aerial parts	Thymol 41, carvacrol 5, *o*-cymene 25, *p*-cymene 14	Inhibition of NO production in in vitro model of LPS-stimulated RAW-264.7 macrophages	IC_50_ = 8 µg/mL Dexamethasone (positive control): IC_50_ = 16 µM	[20]
*Thymus vulgaris* (diplod)/Prague, Czech Republic *Thymus vulgaris* (tetraploid)/Prague, Czech Republic	Aerial parts Aerial parts	Thymol 51, *p*-cymene 20, γ-terpinene 6 Thymol 54, γ-terpinene 22, *p*-cymene 8, α-cadinol 8, caryophyllene 6	In vitro inhibitory activity against ******COX-2 In vitro inhibitory activity against COX-2	Inhibition percentage: 80.96 (500 µg/mL) 70.53 (50 µg/mL) 2.02 (5 µg/mL) Inhibition percentage: 85.57 (500 µg/mL) 83.74 (50 µg/mL) 6.74 (5 µg/mL) Ibuprofen (positive control): 74.52 (5 µg/mL)	[21]
*Thymus vulgaris*/Tlemcen, Algeria *Thymus vulgaris*/Mostaganem, Algeria	Aerial parts Aerial parts	Thymol 67, carvacrol < 0.05, γ-terpinene 10, *p*-cymene 6 Thymol 60, carvacrol < 0.05, γ-terpinene 9, α-pinene 6, *p*-cymene 6, linalool 5	In vivo anti-inflammatory activity through the carrageenan-induced paw edema of Swiss albino mice of both sexes (weighing 25–30 g) In vivo anti-inflammatory activity through the carrageenan-induced paw edema of Swiss albino mice of both sexes (weighing 25–30 g)	Paw thickness (mm): (100 mg/kg–400 mg/kg, between 1–6 h): 2.76–2.36, respectively Paw thickness (mm): (100 mg/kg–400 mg/kg, between 1–6 h): 2.83–2.54, respectively Paw thickness (mm): Diclofenac (positive control) (10 mg/kg, between 1 h–6 h): 2.84–2.07, respectively Paw thickness (mm): Vehicle Tween 80 (Control) (10 mg/kg, between 1 h–6 h): 2.86–3.09, respectively	[22]
*Thymus zygis* chemotype thymol (4 samples)/Murcia, Spain *Thymus zygis* chemotype linalool (2 samples)/Murcia, Spain	Not identified the plant part used Not identified the plant part used	Thymol 30–54, carvacrol 0.4–3, *p*-cymene 14–27, γ-terpinene 8–28, linalool 0.1–5 Thymol 0.1–1, carvacrol 0.4–3, linalool 41–43, myrcene 7–8, terpinen- 4-ol 13–13, γ-terpinene 6–8,	In vitro spectrophotometrically lipoxygenase inhibition In vitro spectrophotometrically lipoxygenase inhibition	IC_50_ (μL/L): 54–73 IC_50_ (μL/L): 299–402	[23]
*Thymus zygis* subsp. *sylvestris*/Parque Natural das Serras de Aire e Candeeiros, Portugal	Aerial parts -collected in flowering stage	Thymol 20, carvacrol 16, *p*-cymene 22, γ-terpinene 7, linalool 6	Production of NO in in vitro model of LPS-stimulated RAW-264.7 macrophages Production of NO in in vitro model of LPS-stimulated in microglia	Production of NO in the presence of four concentrations of the essential oil: 42.67 (0.64 μL/mL), 62.67 (0.32 μL/mL), 91.67 0.16 μL/mL) and 88.00 (0.08 μL/mL Thymol, carvacrol had stronger activity than *p*-cymene and the essential oil (without numeric results, only observed from te figure), particularly in the concentrations ranging from 0.08 to 0.32 μL/mL Production of NO in the presence of four concentrations of the essential oil: 22.00 (0.64 μL/mL), 24.34 (0.32 μL/mL), 26.71 (0.16 μL/mL), 38.94 (0.08 μL/mL) The inhibitory profile triggered by the main compounds was more pronounced in microglia relative to macrophages. All the concentrations of *p*-cymene, thymol and carvacrol tested decreased NO production, quite similar to those obtained in the presence of the essential oil (only based in the observation of the figure	[24]
*Trachyspermum ammi*/*Tiruvannamalai*, Tamil Nadu, India	Seeds	Unprocessed: extraction with ethanol 90% Thymol 42, 3-methoxybutyric acid 5, 9,12-octadecadienoic acid (*Z*,*Z*) 6 Processed: lime treatment followed by extraction with ethanol 90% Thymol 34, 9,12-octadecadienoic acid (*Z*,*Z*) 42,	In vitro inhibitory activity through the protein (egg albumin) denaturation method	Inhibition percentage (1000 µg/mL): 74 Positive control (dichlofenac) (1000 µg/mL): 80 Inhibition percentage (1000 µg/mL): 78 Positive control (dichlofenac) (1000 µg/mL): 80	[26]
*Trachyspermum ammi*/Jorhat, Assam, India	Seeds	Thymol 50, carvacrol 0.1, γ-terpinene 25, *p*-cymene 22	In vitro inhibitory activity through the protein (bovine serum albumin, BSA) denaturation method	IC_50_ = 93.12 µL/mL IC_50_ = 108.61 µL/mL (positive control, diclofenac)	[27]
*Trachyspermum ammi*/Salem, Tamil Nadu, India	Seeds	Not reported, discussion made by the authors is based on the chemical composition of the essential oil of this species found in other articles. According to the authors, the main components of the essential oils of *Trachyspermum ammi* are thymol and carvacrol	In vitro COX-2 inhibition assay Production of nitrite oxide (NO) in in vitro model of LPS-stimulated RAW-264.7 macrophages Production of prostaglandin E2 (PGE2) in in vitro model of LPS-stimulated RAW-264.7 macrophages	IC_50_ = 4.49 µg/mL, IC_50_ = 1 µM (thymol) IC_50_ = 0.8 µM (carvcrol) NO production: 82.54 µM (0.5 µg/mL) 73.94 µM (1.0 µg/mL) 99.64 µM (without essential oil) (PGE2) production: 699.89 pg/mL (0.5 µg/mL) 585.56 pg/mL (1 µg/mL) 776.21 pg/mL (without essential oil)	[28]

*IC_50_: Half maximal inhibitory concentration; **NDGA: Nordihydroguaiatic acid; ***NF-*κ*B: nuclear factor-kappa B; ****TNF-α: tumor necrosis factor-alpha; *****IL: interleukin; ******COX: cyclooxygenase.

## Data Availability

No new data were created or analyzed in this study.

## Supplementary Materials

The following supporting information can be downloaded at: https://www.mdpi.com/article/10.3390/molecules30112450/s1, Table S1. Anti-inflammatory activity of essential oils or volatiles with thymol regardless the concentrations. Refs. [152,153,154,155,156,157,158,159,160,161,162,163,164,165,166] are cited in Supplementary Materials file.
