# Supplementary material for: Anti-Inflammatory Activity of Thymol and Thymol-Rich Essential Oils: Mechanisms, Applications, and Recent Findings
Source: Molecules. 2025 Jun 3;30(11):2450. doi: 10.3390/molecules30112450 (PMC12155930; doi:10.3390/molecules30112450)
Supplement: Supplementary file 1 [file molecules-30-02450-s001.zip › molecules-3593031-supplementary.pdf]

Table S1. Anti-inflammatory activity of essential oils or volatiles with thymol regardless the concentrations.

Species (origin)	Plant part	% of Thymol and carvacrol and main compound (>5%)	Method	Values	References
Achillea millefolium Ilam, Iran	Aerial parts	Thymol 26, carvacrol 10, borneol 16, limonene 15, α-pinene 10	Inhibition of NO production in *in vitro* model of LPS-stimulated RAW-264.7 macrophages	Production of nitrites 15.04 μM; LPS (33.98 μM)	[6]
Anethum graveolens Ilam, Iran	Seeds	Thymol 20, carvacrol 8, limonene 16, α-pinene 9	Inhibition of NO production in *in vitro* model of LPS-stimulated RAW-264.7 macrophages	Production of nitrites 41.04 μM; LPS (33.98 μM)	
Carum copticum (=*Trachyspermum ammi)* Ilam, Iran	Seeds	Thymol 23, carvacrol 6, sabinene 18, borneol 10	Inhibition of NO production in *in vitro* model of LPS-stimulated RAW-264.7 macrophages	Production of nitrites 26.02 μM; LPS (33.98 μM)	
Atractylodes macrocephala Qimen County in eastern China	Rhizomes	Thymol 6, atractylone 39, β-eudesmol 28, hinesol 6	Inhibition of NO and PGE_2 in LPS-stimulated RAW264.7 cells.<br>Real-Time Quantitative PCR (RT-qPCR) Analyses of ******COX-2 and inducible nitric oxide synthase (iNOS) mRNA Transcription in LPS-stimulated RAW264.7 cells	1, 2, 5 and 10 μg/mL reduced the level of PGE_2 by 32, 54, 63 and 85%, respectively.<br>The mRNA levels of iNOS and COX-2 reduced by up to 78.45 % and 90.99 %, respectively, in comparison to model group.	[51]
Carum copticum (=*Trachyspermum ammi)* Unknown	Seeds	Thymol 46, carvacrol 3, γ-terpinene 20, *p*-cymene 11, β-pinene 6	Inhibition of NO production in *in vitro* model of LPS-stimulated RAW-264.7 macrophages	Essential oil: % NO inhibition (80% at 45.0 μg/mL)<br>*p*-cymene: % NO inhibition (48.05% at 45.0 μg/mL)<br>γ-terpinene: % NO inhibition (60.20% at 45.0 μg/mL)<br>β-pinene: % NO inhibition (45.20%	[25]

				at 45.0 μg/mL)<br>thymol: % NO inhibition (85.12% at 45.0 μg/mL)	
Coriandrum sativum<br>from the local farm of Al-Kharj, Riyadh Province of Saudi Arabia	Leaves	Thymol 3, carvacrol 0.4, decanal 11, 1-decanol 18, *trans*-2-dodecen-1-ol 8, menthone 7, *trans*-2-decen-1-ol 5, dodecanal 5	*In vitro* inhibitory activity through the protein (egg albumin) denaturation method<br><br>*In vitro* proteinase inhibitory activity	Inhibitory percentage: 86.17 (1 mg/mL)<br>91.09 (positive control, ibuprofen) (1 mg/mL)<br><br>Inhibitory percentage: 75.65 (0.2 mg/mL)<br>75.67 (positive control, ibuprofen) (0.2 mg/mL)	[52]
Ferulago angulata<br>Yasouj, Iran	Aerial parts	Thymol 8, carvacrol 3, spathulenol 7, *trans*-anetole 6, *p*-menth-2-en-1-ol 5, myristicin 5	*In vivo* anti-inflammatory activity through the croton oil-induced male Swiss mice (20-30 g) ear edema	Both 200 and 100 μL/kg of essential oil produced 66% inhibition of ear edema.<br>Indomethacin (positive control): 82% inhibition at 10 mg/kg)	[7]
Isodon melissoides<br>farm of CSIR-CIMAP, Research Centre Pantnagar Uttarakhan (Supplemental data)- north India	Aerial parts	Thymol 11, carvacrol 45, *p*-cymene 12, γ-terpinene 8, carvacrol acetate 5	Production of proinflammatory mediators: ****TNF-*α*, *****IL-6 and IL-β *in vitro* model of LPS-stimulated HaCaT (human keratinocyte cell line) cells	Inhibition % (1% essential oil): TNF-*α* 56.15 ; IL-6 47.74 ; IL-β 49.3).<br>% Inhibition (1 μg/mL positive control dexamethasone): TNF-*α* 60.20 ; IL-6 75.45 ; IL-β 58.23	[53]
Lantana camara/ Bregbo/Côte d'Ivoire	Flowers (June 2015-June 2016)	Thymol tr (March, April, May)- 34 (July), E-β-caryophyllene 19 (July) – 36 (March, April), *α*-humulene 9 (July) – 20 (April), γ-muurolene 2 (April) – 5 (October, December, March, May), sabinene 0.2 (July) – 5 (April)<br><br>Thymol not detected (September)- 8 (December), E-β-caryophyllene 23 (August) – 37 (April), *α*-humulene 10 (August) – 17 (April), γ-muurolene 4	*In vitro* spectrophotometrically lipoxygenase inhibition	Best activity<br>Flowers of July:<br>*IC_{50} = 17.23 μg/mL<br>IC_{50} = 13.54 μg/mL (positive control, quercetin)<br><br>For detailed information, it is advisable to read the article. Data are presented under graphics	[54]

Flowers (July 2016-June 2017)	(July, August, December) – 8 (January), sabinene 0.23 (January) – 56 (December)	*In vitro* inhibitory activity through the protein (bovine serum albumin, BSA) denaturation method	IC_{50} = 15.45 μg/mL (leaves July 2016)<br>IC_{50} = 15.82 μg/mL (leaves July 2015)<br>IC_{50} = 17.75 μg/mL (flowers 15.31 μg/mL (positive control, diclofenac)<br><br>For detailed information, it is advisable to read the article. Data are presented under graphics
	Thymol not detected (January, February, May) - 18 (August), *E*-β-caryophyllene 24 (September) – 38 (April), *α*-humulene 10 (August) – 20 (March, April), γ-muurolene 2 (February) – 6 (October, November, January), sabinene 3 (July, December) – 9 (May)		
Leaves (June 2015-June 2016)	Thymol 0.2 (January)- 2 (November, May, June), E-β-caryophyllene 32 (June) – 40 (October), *α*-humulene 13 (June) – 21 (January), γ-muurolene 4 (June, July, March, April) – 7 (October), sabinene 3 (October, January) – 11 (June)		
Leaves (June 2016-June 2017)	Thymol 0.2 (March)- 41 (August), *E*-β-caryophyllene 7 (August) – 25 (September, February), *α*-humulene 3 (August) – 15 (February), linalool 1 (March, April) – 9 (July, November), *E*-β-farnesene 1 (August) – 7 (November), isospathulenol 1 (August) – 6 (October, November, March, April), *α*-muurolene 1 (August) – 5 (November), *p*-cymene not detected (March, April) – 6 (August), γ-terpinene not detected (March, April) – 6 (August)		
	Thymol not detected (September)- 13		

Stems (June 2015-June 2016)	(November), *E*-β-caryophyllene 14 (September, October) – 25 (January, February, March), *α*-humulene 7 (October) – 15 (February, March), linalool 0.2 (October) – 6 (August), *E*-β-farnesene 3 (January) – 6 (December), isospathulenol 2 (November, January, February) – 5 (August), *α*-muurolene 3 (November, January, February) – 5 (December), γ-muurolene 2 (October) – 6 (January
	Thymol tr (March)- 28 (July), *E*-β-caryophyllene 15 (July) – 30 (January, April), *α*-humulene 7 (July) – 17 (April, May), linalool 1 (April) – 6 (July, May), *E*-β-farnesene 3 (June, July) – 5 (October, December, January, April, May)
Stems (July 2016-June 2017)	Thymol not detected (September)- 22 (December, June), *E*-β-caryophyllene 11 (August) – 37 (February), *α*-humulene 6 (August) – 18 (February), linalool 2 (February) – 6 (July), *E*-β-farnesene 2 (August) – 6 (February), neral not detected (February, May) – 15 (August), geranial (citral) not detected (July, September, December, February, April, May, June) – 23 (August)

Fruits (June 2015-June 2016)

Fruits (July 2016-June 2017)

Lantana rhodesiensis/Kapélé, Côte d'Ivoire	Leaves (June, July, August, September)	Thymol 3, 13, 8, 5; β-caryophyllene 25, 22, 27, 23; α-copaene 12, 10, 11, 11; α-humulene 9, 8, 10, 9; δ-cadinene 9, 8, 10, 9; limonene 7, 2, 1, 7; caryophyllene oxide 4, 5, 4, 4	*In vitro* inhibitory activity through the protein (bovine serum albumin, BSA) denaturation method	IC_{50} = 26.18 µg/mL (leaves Kapélé); IC_{50} = 28.16 µg/mL (leaves Nyanbélégé); IC_{50} = 24.76 µg/mL (positive control, diclofenac)	[55]
		Thymol 8, 15, 8, 10; β-caryophyllene 19, 14, 19, 21; α-copaene 12, 7, 12, 13; α-humulene 7, 5, 7, 6; δ-cadinene 11, 11, 11, 13; limonene 1, tr, 0.2, tr; caryophyllene oxide 5, 8, 7, 7		For detailed information, it is advisable to read the article. Data are presented under graphics	
	Stems (June, July, August, September)	Thymol 17, β-caryophyllene 23; α-copaene 9; α-humulene 8; δ-cadinene 7; limonene tr; caryophyllene oxide 4			
		Thymol 3, 3, 4, 17; β-caryophyllene 25, 24, 20, 21; α-copaene 12, 12, 11, 10; α-humulene 10, 9, 8, 8; δ-cadinene 10, 10, 8, 7; limonene 6, 2, 5, 4; caryophyllene oxide 4, 6, 8, 5			
		Thymol 4, 4, 3, 9; β-caryophyllene 22, 18, 18, 21; α-copaene 15, 12, 15, 12; α-humulene 8, 7, 6, 6; δ-cadinene 12, 12, 11, 11; limonene 2, tr, 1, 1; caryophyllene oxide 4, 6, 8, 6			
	Fruits (July)	Thymol 1, β-caryophyllene 25; α-copaene 12; α-humulene 10; δ-cadinene 11; limonene 7; caryophyllene oxide 4			

Lantana rhodesiensis/Nyanbélégé, Côte d'Ivoire	Leaves (June, July, August, September)				
	Stems (June, July, August, September)				
	Fruits (July)				
Lippia multiflora/ Gampéla, Burkina Faso	No identified plant part	Thymol 12, carvacrol 2, *p*-cymene 25, β-caryophyllene 13, γ-terpinene 9, thymyl	*In vitro* spectrophotometrically lipoxygenase inhibition	97% inhibition for a concentration of 8 mg/mL. Quercetin (positive	[56]

	used	acetate 8,		control) 52% inhibition for a concentration of 100 μg/mL	
Machilus konishii/ Pingtung County/ Taiwan (China)	Leaves	Thymol 12, α-pinene 34, β-pinene 14, β-caryophyllene 5	Production of NO in *in vitro* model of LPS-stimulated RAW-264.7 macrophages	Production of NO: Essential oil 12.5 μg/mL (15-20 μM) – 200 μg/mL (< 5 μM) (only based in the observation of the Figure)<br><br>α-Pinene, β-pinene and thymol were also tested and thymol had the best activity (only based in the observation of the Figure)	[57]
Monarda didyma/ Urbino, Italy	Aerial parts of the plant at flowering	Thymol 8, carvacrol 17, *p*-cymene 16, γ-terpinene 8, carvacrol methyl ether 6, 1-octen-3-ol 5, thymol methyl ether 5	Regulation of microRNA-146a, IL-6, and IRAK-1 (interleukin-1 receptor-associated kinase) in LPS-stimulated U937 cells	At 0.5 μL/mL, there was microRNA-146a up-regulation, and a decreased expression of IRAK-1 and IL-6<br><br>For more information is advisable to read the article	[58]
Mosla dianthera (Buch.-Ham. ex Roxb.) Maxim./ Uttarakhand, India	Aerial parts	Thymol 11, carvone 42, *p*-cymene 10, *Z*-β-bisabolene 7	*In vitro* inhibitory activity through the protein (hen's egg albumin) denaturation method<br><br>*In vitro* Inhibition of NO production	IC_{50} = 28.85 μg/mL<br>IC_{50} = 27.32 μg/mL (carvone)<br>IC_{50} = 23.67 μg/mL (positive control, diclofenac)<br><br>IC_{50} = 17.10 μg/mL<br>IC_{50} = 18.07 μg/mL (carvone)<br>IC_{50} = 10.95 μg/mL (positive control, ascorbic acid)	[59]
Nigella sativa Ilam, Iran	Seeds	Thymol 10, carvacrol 1, *p*-cymene 32, thymoquinone 20, carvone 12, camphene 11, β-pinene 7, α-thujene 6, γ-terpinene 5	Inhibition of NO production in *in vitro* model of LPS-stimulated RAW-264.7 macrophages	% of NO inhibition: essential oil: 82 (25.0 μg/mL), the constituents: *p*-cymene 55 (25.0 μM), γ-terpinene 55 (25.0 μM), α-pinene 53 (25.0 μM), thymol 80 (25.0 μM), α-thujene 51 (25.0 μM), thymoquinone 90 (25.0 μM)	[60]
Ocimum basilicum/ Kairouan,	Seeds	Solid-liquid Soxhlet extraction using *n*-	Inhibition of NO production in LPS-	At 150 μg/mL, NO production	[8]

Tunisia		hexane:<br>Concentration (µg/g): Thymol 33, luteolin 9<br><br>2-Methyltetrahydro-furan (MeTHF)<br>Concentration (µg/g): Thymol 128, rosmarinic acid 21, luteolin 9, gallic acid 7, chlorogenic acid 7, ellagic acid 6, circimaritin 6	induced murine macrophage RAW 264.7 cell line	decreased by 60%<br><br>At 150 µg/mL, NO production decreased by 64%	
Oliveria decumbens/ Dil village, Iran	Aerial parts	Extraction with ethanol:H_2O (70:30) and then fractionation:<br>*n*-hexane:<br>thymol 56, carvacrol 38<br>dichloromethane:<br>thymol 53, carvacrol 41	Oxidative burst assay using luminol-enhanced chemiluminescence technique with blood HBSS++ (Hanks Balanced Salt Solution), serum opsonized zymosan (SOZ) and intracellular reactive oxygen species	Crude extract: IC_{50} = 21.7 µg/mL<br>*n*-Hexane: IC_{50} = 22.6 µg/mL<br>Dichloromethane:<br>IC_{50} = 15.8 µg/mL<br>Positive control (Ibuprofen):<br>IC_{50} = 11.2 µg/mL<br>Thymol: not active (>100 µg/mL)<br>Carvacrol: not active (>100 µg/mL)	[9]
Oregano (the species is unknown)	No identified plant part used	Thymol, carvacrol, β-caryophyllene<br>The concentrations were not provided by the authors	Expression level of CD80 in RAW.246.7 macrophages treated with the low-serum conditioned medium and analyzed by flow cytometry<br>The assays were done with electrospun poly (L-lactide-coglycolide) /gelatin (PG)-based fibrous dressings with diverse additives	Mean fluorescence intensity (%):<br>Control: 14.0<br>PG: 23.7<br>PG@BP(amino acid sequence, DRVQRQTTTVVA: 21.3<br>PG@O(essential oil of oregano): 14.0<br>PG@BPO: 13.8	[61]
Origanum compactum/ Boulemane,, Marocco	Aerial parts (leaves, flowers, and stems)<br>July 2021	Thymol 19, carvacrol 46, β-pinene 13, *p*-cymene 8	*In vitro* spectrophotometrically lipoxygenase inhibition<br><br>*In vivo* anti-inflammatory activity through the carrageenan-induced Wistar rats (160 to 190 g)paw edema<br><br>*In vitro* spectrophotometrically lipoxygenase inhibition	**IC_{50} µg/mL: 0.68; quercetin (positive control): 0.29 µg/mL<br><br>Inhibition % of edema (1 h 30 min: 56.53; 3 h: 64.95; 60.67).<br>Indomethacin (positive control:<br>1 h 30 min: 70.34; 3 h: 70.40; 62.17<br><br>IC_{50} µg/mL: 1.33; quercetin	[62]

				(positive control): 0.29 μg/mL	
Origanum compactum/ Taounate, Morocco	dried flowering tops (a mixture of leaves, flowers, and stems)	Thymol 13, 3-carene 20, *o*-cymene 11	*In vivo* anti-inflammatory activity through the carrageenan-induced Wistar rats (160 to 190 g) paw edema	Inhibition % of edema (1 h 30 min: 38.86; 3 h: 39.00; 32.02). Indomethacin (positive control: 1 h 30 min: 70.34; 3 h: 70.40; 62.17)	
Origanum floribundum/ Lakhdaria, Algeria	Aerial parts at flowering stage (July 2011)	Thymol 34, carvacrol 9, γ-terpinene 20, *p*-cymene 16,	*In vitro* spectrophotometrically lipoxygenase inhibition	IC_{50}: 125.7 μg/mL **NDGA (positive control): IC_{50} = 63.4 μg/mL	[10]
Origanum heracleoticum (5 samples) / Agrigento, Italy	Leaves and flowers	Thymol 47-65, carvacrol 3-5, γ-terpinene 13-22, *p*-cymene 4-5, carvacrol methyl ether 3-4	Gene expression analysis of NF-kB pathway in Caco-2 cells treated with TNF-α	An increase trend of IL-1α, IL-6 and IL-8 gene expression was observed when compared to the treatment with TNF-α, without statistical significance It is advisable to read the article, data presented as graphics	[11]
Origanum heracleoticum (6 samples)/ Calabria ,Italy	No identified plant part used	Thymol 9104-54459, carvacrol 3311-68379, *o*-cymene 3304-48176, γ-terpinene 24-19133, carvacrol-methyl ether 2658-19844, β-caryophyllene 1182-16107, γ-muurolene 1364-10824 (unknown unities, the compounds were chosen if at least one value is >10,000	Inhibition of NO production in lipopolysaccharide (LPS)-induced murine macrophage RAW 264.7 cell line	IC_{50} = 32.77-170.9 μg/mL. Positive controls (indomethacin and L-NAME): IC_{50} = 58.00 and 45.86, respectively)	[12]
Origanum minutiflorum (supplemental information) *Origanum vulgare* (article) Unknown collection place	No identified plant part used	Thymol 3, carvacrol 47, linalool 33	Inflammation-related parameters, such as NO, MPO, and TNF-α, were determined in the kidney tissue of adult (four months old) male Wistar rats weighing 300–350 g rats, 24 h after L-arginine application	The administration of essential oil, thymol and carvacrol prevented an increase in NO concentration and MPO activity, and did not affect an increase in TNF-α concentration.	[63]

				Treatment with allopurinol (positive control) prevented an increase in all three of the tested inflammation-related parameters.<br><br>It is advisable to read the article. Data are under graphics	
Origanum vulgare/ Pisa, Italy	No identified plant used (The EO have been purchased)	Thymol 1, carvacrol 56, γ-terpinene 14, *p*-cymene 11	*In vitro* inhibitory activity through the protein (bovine serum albumin, BSA) denaturation method	IC_{50} (*O. vulgare*/*C. ladanifer* or *O. vulgare*/*J. communis*) < IC_{50} (*C. ladanifer* or *C. aurantium*, *C. ladanifer* or *J. communis*) < IC_{50} (*O. vulagre* or *O. vulgare*/*C. aurantium*) < IC_{50} (diclofenac, positive control)<br><br>It is advisable to read the article. Data are under graphics.	[64]
Citrus aurantium var. *amara* /Pisa, Italy		Limonene 88	*In vitro* inhibitory activity through the protein BSA denaturation method		
			In vitro inhibitory activity through the protein BSA denaturation method		
Cistus ladanifer/ Torino, Italy		Camphene 37, bornyl acetate 22, α-pinene 14, tricyclene 5	*In vitro* inhibitory activity through the protein BSA denaturation method		
Juniperus communis/ Pisa, Italy		α-Pinene 42, sabinene 12, myrcene 11, limonene 6	*In vitro* inhibitory activity through the protein BSA denaturation method		
O. vulgare / *Citrus aurantium (1:1)*		Not determined	*In vitro* inhibitory activity through the protein BSA denaturation method	Sum of the fractional inhibitory concentration index (ΣFIC) = 1.774 interaction indifference	
		Not determined	*In vitro* inhibitory activity through the protein BSA denaturation method	ΣFIC = 0.964 interaction additive	

O. vulgare / Cistus ladanifer (1:1)		Not determined			
				ΣFIC = 0.862 interaction additive	
Origanum vulgare / Juniperus comunis					
Origanum vulgare (3 samples)/ South-East of Spain	Steams	Thymol 2-6, carvacrol 59-74, γ-terpinene 2-11, *p*-cymene 4-8	*In vitro* spectrophotometrically lipoxygenase inhibition	IC_{50} = 148.0 – 251.5 µL/L. IC_{50} of positive controls (thymol, limonene, *p*-cymene, carvacrol, linalool, NDGA) = 150, 356, 486, 2271, 3346, 339 µM, respectively	[65]
Origanum vulgare ssp. *viridulum* x *Origanum vulgare* ssp. *hirtum* (3 samples)/ Agrigento, Italy	Leaves and flowers	Thymol 0.1-3, carvacrol 81-85, γ-terpinene 5-7	Gene expression analysis of NF-kB pathway in Caco-2 cells treated with TNF-*α*	Slight decrease in IkB*α*, IL-6 and IL-8 gene expression was observed when compared to the treatment with TNF-*α*, but without statistical significance<br>It is advisable to read the article, data presented as graphics	[12]
Salacia pallescens/ Idi-Ayunre, Ibadan, Nigeria	Leaves	Extraction with methanol 50%: Thymol 30, 3-carene 16, p-cymene 12, caffeine 8, hexadecanoic acid 6, bicycle[3.1.1]hept-2-ene, 2,6-dimethyl-6-(4-methyl-3-pentenyl) 6, caryophyllene 5	*In vitro* inhibition of nitrite oxide (NO) production	IC_{50} = 49.49 µg/mL<br>IC_{50} = 48.74 µg/mL (positive control, ascorbic acid)	[13]
			Inhibition of interleukin 6 (IL-6) production in *in vitro* model of LPS-stimulated RAW-264.7 macrophages	Nine-fold reduction in LPS induced IL-6 production in RAW-264.7 macrophages pre-treated with 400 µg/mL of the extract.<br>1.4-fold reduction in LPS induced IL-6 production in RAW-264.7 macrophages pre-treated with 50 µg/mL of the extract	
Siddhalepa Asamodagam Spirit (water distillate	Seeds	The water distillate was then extracted with ethyl acetate:	Heat induced hemolysis principle using human red blood cell stabilization	IC_{50} = 0.57 mg/mL<br>Positive control (aspirin):	[29]

derived from *Trachyspermum roxburghianum* seeds/ Pakistan	(	Thymol 93, carvacrol 1	method	IC_{50} = 0.24 mg/mL	
Thymbra capitata (3 samples)/ South-East of Spain	Steams	Thymol 0.2-2, carvacrol 69-76, γ-terpinene 6-8, *p*-cymene 7-7	*In vitro* spectrophotometrically lipoxygenase inhibition	IC_{50} = 167.1 – 184.1 µL/L. IC_{50} of positive controls (thymol, limonene, *p*-cymene, carvacrol, linalool, NDGA) = 150, 356, 486, 2271, 3346, 339 µM, respectively	[65]
Thymus atlanticus/ Errachidia region, Morocco	Aerial parts	Thymol 24, carvacrol 23, γ-terpinene 21, *p*-cymene 19	*In vivo* phenol induced ear edema in Wister albino rats of both sexes weighing 100-120 g	The application of phenol alone has developed in the control group an ear inflammation of 77.59%<br>The topical application of essential oil (1 mg/ear) has reduced the ear edema with a percentage of 19.39%.<br>Indomethacin (positive control) (1 mg/ear) has reduced the ear edema with a percentage of 14.71%	[14]
Thymus caespitius/Azores, Portugal	Plant material was collected during the flowering phase –	Thymol 34, carvacrol 11, *p*-cymene 12, thymol acetate 8, γ-terpinene 5	NO scavenging activity<br><br>*In vitro* spectrophotometrically lipoxygenase inhibition	IC_{50} = 0.3 mg/mL<br><br>IC_{50} = 0.1 mg/mL	[15]
Thymus linearis/ Harinagar and Dhanachuli,India	Aerial parts	Thymol 67. carvacrol 3, *p*-cymene 10	*In vivo* anti-inflammatory activity through the carrageenan-induced paw edema of Swiss albino mice	Inhibition % (5%) after 4 h and 24 h = 3.83 and 6.38, respectively<br>Inhibition % (10%) after 4 h and 24 h = 5.19 and 9.52, respectively<br>Inhibition % (20%) after 4 h and 24 h = 9.73 and 14.6, respectively<br>Positive control (Ibuprofen): Inhibition % (0.004) after 4 h and 24 h = 26.07 and 37.18, respectively	[16]

			In vivo sub-acute anti-inflammatory activity through the formaldehyde induced arthritis in the right hind paw of Swiss albino mice (formaldehyde solution 1% injected on the first day of the experiment. Samples administered orally every day of the experiment. The results were recorded every day till the end (10 days). For knowing all results it is advisable to read the reference	Volume of inflammation (mm^3) (5%): day 0, day 5, day 10 = 2.26, 2.47, 2.51<br>Volume of inflammation (mm^3) (10%): day 0, day 5, day 10 = 2.21, 2.38, 2.38<br>Volume of inflammation (mm^3) (20%): day 0, day 5, day 10 = 2.19, 2.32, 2.26<br>Volume of inflammation (mm^3) saline water (control): day 0, day 5, day 10 = 2.13, 2.39, 2.37<br>Volume of inflammation (mm^3) positive control (Ibuprofen) (0.001%): day 0, day 5, day 10 = 2.11, 2.19, 2.15	
Thymus sipyleus Boiss. subsp. *sipyleus* var. *sipyleus*/ Sivas, Turkey	Aerial parts	Thymol 66, carvacrol 3, *p*-cymene 9, γ-terpinene 9	*In vitro* spectrophotometrically lipoxygenase inhibition	12% inhibition for a concentration of 100 µg/mL. NDGA (positive control) 100% inhibition for a concentration of 100 µg/mL	[17]
Thymus vulgaris/ Szigetvár city, Hungary	Plant material - No identified the part of plant used	Beginning of flowering: Thymol 56, carvacrol 2, *p*-cymene 13, γ-terpinene 15<br><br>End of flowering: Thymol 54, carvacrol 3, *p*-cymene 21, γ-terpinene 6	Inhibition of cytokine production through *P. aeruginosa* LPS-activated THP-1 macrophage cells	Inhibition of the production of IL-6, IL-8, IL-1β, TNF-α. Thymol (positive control) had better activity<br><br>These results are based on the figures' observation. It is advisable to read the article<br><br>There is not a decrease of the four examined proinflammatory cytokine IL-6, IL-8, IL-β, TNF-α expression both at the mRNA and protein levels	[18]

				These results are based on the figures' observation. It is advisable to read the article	
Thymus vulgaris (purchased)	No identified plant part used	Thymol 49, carvacrol 3, *p*-cymene 29	Male C57BL/6J mice with 8 (young)- and 53 (old)-week<br>Male C57BL/6J mice with 53 week + *T. vulgaris* essential oil<br>Control: without *T. vulgaris* essential oil.<br>Gene expression of TNF-α, IL-6, IL-1β in cerebellum, hippocampus, liver, brain's cerebral cortex<br><br>Gene expression of TNF-α, IL-6, IL-1β in the age-accelerated mouse embryonic fibroblast NIH/3T3 cell line	The essential oil group showed lower<br>expression of IL-1β in the liver and cerebellum and<br>IL-6 in the hippocampus compared to the control group<br><br>24-h pretreatment<br>with essential oil, followed by co-treatment with essential oil and mitomycin C suppressed the gene expression of pro-inflammatory cytokines<br><br>It is advisable to read the article since the results are presented under graphics	[19]
Thymus vulgaris (purchased)	Not reported	Thymol 49, carvacrol 3, *p*-cymene 29	Aging-related and pro-inflammatory cytokine gene expression in the liver, hippocampus, cerebellum, and cerebral cortex of mice fed with the thyme EO 250 mg/kg/day (0.2% (w/w)<br><br>Aging-related and pro-inflammatory	Lower levels of $p16^{INK4A}$ in the hippocampus than control; lower expression of *Il-1b* in the liver and cerebellum, and *Il6* in the hippocampus than the control; lower expression of *p65* and *p50* (two transcription factors of ***NF-κB) than the control<br><br>Lower levels of *Il6* and *Ccl2*	[19]

			cytokine gene expression in age-accelerated NIH-3T3 cells. Thyme oil concentrations: 30, 60, 120 μg/mL	mRNA expression (dose-dependent)<br><br>It is advisable to read the article since the results are presented under graphics	
Thymus vulgaris / Póvoa de Lanhoso, Portugal	Aerial parts	Thymol 41, carvacrol 5, *o*-cymene 25, *p*-cymene 14	Inhibition of NO production in *in vitro* model of LPS-stimulated RAW-264.7 macrophages	IC_{50} = 8 μg/mL<br>Dexamethasone (positive control): IC_{50} = 16 μM	[20]
Thymus vulgaris (diplod)/ Prague, Czech Republic	Aerial parts	Thymol 51, *p*-cymene 20, γ-terpinene 6	*In vitro* inhibitory activity against COX-2	Inhibition percentage:<br>80.96 (500 μg/mL)<br>70.53 (50 μg/mL)<br>2.02 (5 μg/mL)	[21]
Thymus vulgaris (tetraploid)/ Prague, Czech Republic	Aerial parts	Thymol 54, γ-terpinene 22, *p*-cymene 8, *α*-cadinol 8, caryophyllene 6	*In vitro* inhibitory activity against COX-2	Inhibition percentage:<br>85.57 (500 μg/mL)<br>83.74 (50 μg/mL)<br>6.74 (5 μg/mL)<br><br>Ibuprofen (positive control): 74.52 (5 μg/mL)	
Thymus vulgaris/ Tlemcen,Algeria	Aerial parts	Thymol 67, carvacrol <0.05, γ-terpinene 10, *p*-cymene 6	*In vivo* anti-inflammatory activity through the carrageenan-induced paw edema of Swiss albino mice of both sexes (weighing 25 - 30 g)	Paw thickness (mm): (100 mg/kg – 400 mg/kg, between 1-6 h): 2.76-2.36, respectively	[22]
Thymus vulgaris/ Mostaganem, Algeria	Aerial parts	Thymol 60, carvacrol <0.05, γ-terpinene 9, *α*-pinene 6, *p*-cymene 6, linalool 5	*In vivo* anti-inflammatory activity through the carrageenan-induced paw edema of Swiss albino mice of both sexes (weighing 25 - 30 g)	Paw thickness (mm): (100 mg/kg – 400 mg/kg, between 1-6 h): 2.83-2.54, respectively<br><br>Paw thickness (mm): Diclofenac	

				(positive control) (10 mg/kg, between 1 h – 6 h): 2.84-2.07, respectively<br><br>Paw thickness (mm): Vehicle Tween 80 (Control) (10 mg/kg, between 1 h – 6 h): 2.86-3.09, respectively	
Thymus zygis chemotype thymol (4 samples)/ Murcia, Spain	No identified plant part used	Thymol 30-54, carvacrol 0.4-3, *p*-cymene 14-27, γ-terpinene 8-28, linalool 0.1-5	*In vitro* spectrophotometrically lipoxygenase inhibition	IC_{50} (µL/L): 54-73	[23]
Thymus zygis chemotype linalool (2 samples)/ Murcia, Spain	No identified plant part used	Thymol 0.1-1, carvacrol 0.4-3, linalool 41-43, myrcene 7-8, terpinen- 4-ol 13-13, γ-terpinene 6-8,	*In vitro* spectrophotometrically lipoxygenase inhibition	IC_{50} (µL/L): 299-402	
Thymus zygis subsp. *sylvestris/ Parque Natural das Serras de Aire e Candeeiros, Portugal*	Aerial parts - collected in flowering stage	Thymol 20, carvacrol 16, *p*-cymene 22, γ-terpinene 7, linalool 6,	Production of NO in *in vitro* model of LPS-stimulated RAW-264.7 macrophages	Production of NO in the presence of four concentrations of the essential oil: 42.67 (0.64 µL/mL), 62.67 (0.32 µL/mL), 91.67 0.16 µL/mL) and 88.00 (0.08 µL/mL Thymol, carvacrol had stronger activity than *p*-cymene and the essential oil (without numeric results, only observed from te Figure), particularly in the concentrations ranging from 0.08 to 0.32 µL/mL<br><br>Production of NO in the presence of four concentrations of the essential oil: 22.00	[24]

			Production of NO in *in vitro* model of LPS-stimulated in microglia	(0.64 µL/mL), 24.34 (0.32 µL/mL), 26.71<br>(0.16 µL/mL), 38.94 (0.08 µL/mL)<br>The inhibitory profile triggered by the main compounds<br>was more pronounced in microglia relative to macrophages.<br>All the concentrations of *p*-cymene, thymol and carvacrol tested decreased NO production, quite similar to those obtained in the<br>presence of the essential oil (only based in the observation of the Figure	
Trachyspermum ammi/ *Tiruvannamalai,* Tamil Nadu, India	Seeds	Unprocessed: extraction with ethanol 90%<br>Thymol 42, 3-methoxybutyric acid 5, 9,12-octadecadienoic acid (*Z,Z*) 6<br><br>Processed: lime treatment followed by extraction with ethanol 90%<br>Thymol 34, 9,12-octadecadienoic acid (*Z,Z*) 42,	*In vitro* inhibitory activity through the protein (egg albumin) denaturation method	Inhibition percentage (1000 µg/mL): 74<br>Positive control (dichlofenac) (1000 µg/mL): 80<br><br>Inhibition percentage (1000 µg/mL): 78<br>Positive control (dichlofenac) (1000 µg/mL): 80	[26]
Trachyspermum ammi/ Jorhat, Assam, India	Seeds	Thymol 50, carvacrol 0.1, γ-terpinene 25, *p*-cymene 22	*In vitro* inhibitory activity through the protein (bovine serum albumin, BSA) denaturation method	IC_{50} = 93.12 µL/mL<br>IC_{50} = 108.61 µL/mL (positive control, diclofenac)	[29]
Trachyspermum ammi/ Salem, Tamil Nadu, India	Seeds	Not reported, discussion made by the authors is based on the chemical composition of the essential oil of this species found in other articles.<br>According to the authors, the main components of the essential oils of	*In vitro* COX-2 inhibition assay<br><br>Production of nitrite oxide (NO) in *in vitro* model of LPS-stimulated RAW-264.7	IC_{50} = 4.49 µg/mL, 4.15, 9.23<br>IC_{50} = 1 µM (thymol)<br>IC_{50} = 0.8 µM (carvcrol)<br><br>NO production:<br>82.54 µM (0.5 µg/mL)	[28]

	Trachyspermum ammi are thymol and carvacrol	macrophages<br><br>Production of prostaglandin E2 (PGE2) in *in vitro* model of LPS-stimulated RAW-264.7 macrophages	73.94 μM (1.0 μg/mL)<br>99.64 μM (without essential oil)<br><br>(PGE2) production:<br>699.89 pg/mL (0.5 μg/mL)<br>585.56 pg/mL (1 μg/mL)<br>776.21 pg/mL (without essential oil)

*IC_{50}: Half maximal inhibitory concentration; **NDGA: Nordihydroguaiatic acid; ***NF-κB: nuclear factor-kappa B; ****TNF-α: tumor necrosis factor-alpha; *****: IL: interleukin; ******COX: cyclooxygenase.

References

6. Kazemi, M. Chemical composition and antimicrobial, antioxidant activities and anti-inflammatory potential of *Achillea millefolium* L., *Anethum graveolens* L., and *Carum copticum* L. essential oils. *J. Herb. Med*. **2015**, *5*, 217–222.
7. Hajhashemi, V.; Kopaei, S.R.; Sajjadi, S.E. Anti-nociceptive and anti-inflammatory effects of *Ferulago angulata*. *Immunopathol. Persa* **2020**, *6*, e28. https://doi.org/10.34172/ipp.2020.28.
8. Bourgou, S.; Rebey, I.B.; Kaab, S.B.; Hammami, M.; Dakhlaoui, S.; Sawsen, S.; Msaada, K.; Isoda, H.; Ksouri, R.; Fauconnier, M.-L. Green solvent to substitute hexane for bioactive lipids extraction from black cumin and basil seeds. *Foods* **2021**, *10*, 1493. https://doi.org/10.3390/foods10071493.
9. Mirahmad, A.; Ghoran, S.H.; Alipour, P.; Taktaz, F.; Hassan, S.; Naderian, M.; Moradalipour, A.; Faizi, M.; Kobarfard, F.; Ayatollahi, S.A. *Oliveria decumbens* Vent. (Apiaceae): Biological screening and chemical compositions. *J. Ethnopharmacol*. **2024**, *318*, 117053. https://doi.org/10.1016/j.jep.2023.117053.
10. Kerbouche, L.; Hazzit, M.; Ferhat, M.-A.; Baaliouamer, A.; Miguel, M.G. Biological activities of essential oils and ethanol extracts of *Teucrium polium* subsp. *capitatum* (L.) Briq. And *Origanum floribundum* Munby. *J. Essent. Oil Bear. Plants* **2015**, *18*, 1197–1208.
11. Marrelli, M.M.; Araniti, F.; Abenavoli, M.R.; Statti, G.; Conforti, F. Potential health benefits of *Origanum heracleoticum* essential oil: Phytochemical and biological variability among different Calabrian populations. *Nat. Prod. Commun*. **2018**, *13*, 1183–1187.
12. Zinno, P.; Guantario, B.; Lombardi, G.; Ranaldi, G.; Finamore, A.; Allegra, S.; Mammano, M.M.; Fascella, G.; Raffo, A.; Roselli, M. Chemical composition and biological activities of essential oils from *Origanum vulgare* genotypes belonging to the carvacrol and thymol chemotypes. *Plants* **2023**, *12*, 1344. https://doi.org/10.3390/plants12061344.
13. Abiodun, O.O.; Oke, T.A.; Adeyemi, F.O.; Oshinloye, A.O.; Akande, A.O. *Salacia pallescens* Oliv. (Celastraceae) scavenges free radicals and inhibits pro-inflammatory mediators in lipopolysaccharide-activated RAW cells 264.7 macrophages. *Turk. J. Pharm. Sci*. **2021**, *18*, 702–709.
14. Elbouny, H.; Ouahzizi, B.; El-Guourrami, O.; Drioua, S.; Mbarek, A.N.; Sellam, K.; Alem, C. Chemical profile and biological properties of the essential oil of *Thymus atlanticus* (Ball) Roussine. *South Afr. J. Bot*. **2022**, *151*, 475–480.
15. Aazza, S.; El-Guendouz, S.; Miguel, M.G.; Antunes, M.D.; Faleiro, M.L.; Correia, A.I.; Figueiredo, A.C. Antioxidant, anti-inflammatory and anti-hyperglycaemic activities of essential oils from *Thymbra capitata*, *Thymus albicans*, *Thymus caespititius*, *Thymus carnosus*, *Thymus lotocephalus* and *Thymus mastichina* from Portugal. *Nat. Prod. Commun*. **2016**, *11*, 1029–1038.
16. Chandra, M.; Prakash, O.; Bachheti, R.K.; Kumar, M.; Pant, A.K. Essential oil composition, phenolic constituents, antioxidant and pharmacological activities of *Thymus linearis* Benth. Collected from Uttarakhand region of India. *J. Essent. Oil Bear. Plants* **2016**, *19*, 277–289.
17. Demirci, F.; Karaca, N.; Tekin, M.; Demirci, B. Anti-inflammatory and antibacterial evaluation of *Thymus sipyleus* Boiss. subsp. *sipyleus* var. *sipyleus* essential oil against rhinosinusitis pathogens. *Microb. Pathog*. **2018**, *122*, 117–121.
18. Pandur, E.; Micalizzi, G.; Mondello, L.; Horváth, A.; Sipos, K.; Horváth, G. Antioxidant and anti-inflammatory effects of thyme (*Thymus vulgaris* L.) essential oils prepared at different plant phenophases on *Pseudomonas aeruginosa* LPS-activated THP-1 macrophages. *Antioxidants* **2022**, *11*, 1330. doi.org/10.3390/antiox11071330.
19. Warman, D.J.; Jia, H.; Kato, H. Effects of thyme (*Thymus vulgaris* L.) essential oil on aging-induced brain inflammation and blood telomere attrition in chronologically aged C57BL/6J mice. *Antioxidants* **2023**, *12*, 1178. https://doi.org/10.3390/antiox12061178.

20. Spréa, R.M.; Caleja, C.; Finimundy, T.C.; Calhelha, R.C.; Pires, T.C.S.P.; Amaral, J.S.; Prieto, M.A.; Ferreira, I.C.F.R.; Pereira, E.; Marros, L. Chemical and bioactive evaluation of essential oils from edible and aromatic Mediterranean Lamiaceae plants. *Molecules* **2024**, *29*, 2827. https://doi.org/10.3390/molecules29122827.
21. Gupta, N.; Bhattacharya, S.; Dutta, A.; Tauchen, J.; Landa, P.; Urbanová, K.; Houdková, M.; Fernández-Cusimamani, E.; Leuner, O. Synthetic polyploidization induces enhanced phytochemical profile and biological activities in *Thymus vulgaris* L. essential oil. *Sci. Rep*. **2024**, *14*, 5608. https://doi.org/10.1038/s41598-024-56378-7.
22. Abdelli, W.; Bahri, F.; Romane, A.; Höferl, M.; Wanner, J.; Schmidt, E.; Jirovetz, L. Chemical composition and anti-inflammatory activity of Algerian *Thymus vulgaris* essential oil. *Nat. Prod. Commun*. **2017**, *12*, 611–614.
23. Cutillas, A.-B.; Carrasco, A.; Martinez-Gutierrez, R.; Tomas, V.; Tudela, J. Thyme essential oils from Spain: Aromatic profile ascertained by GC-MS, and their antioxidant, anti-lipoxygenase and antimicrobial activities. *J. Food Drug. Anal*. **2018**, *26*, 529–544.
24. Rodrigues, V.; Cabral, C.; Évora, L.; Ferreira, I.; Cavaleiro, C.; Cruz, M.T.; Salgueiro, L. Chemical composition, anti-inflammatory activity and cytotoxicity of *Thymus zygis* L. subsp. *sylvestris* (Hoffmanns. & Link) Cout. Essential oil and its main compounds. *Arab. J. Chem*. **2019**, *12*, 3236–3243.
25. Kazemi, M. Chemical composition, antimicrobial, antioxidant and anti-inflammatory activity of *Carum copticum* L. essential oil. *J. Essent. Oil Bear. Plants*. **2014**, *17*, 1040–1045.
26. Vanitha, K.G.; Natarajan, A.; Sudhkar, N.; Hirad, A.H.; Alarfaj, A.A.; Arulselvan, P.; Raja, R. Enhancing therapeutic potential: Investigating traditional detoxification methods and assessing their influence on anti-microbial efficacy, phytochemical composition, heavy metal content and anti-inflammatory properties in *Trachyspermum ammi*. *Ind. J. Pharm. Edu. Res*. **2025**, *59*, 230–241.
27. Dutta, P.; Sarma, N.; Saikia, S.; Gogoi, R.; Begum, T.; Lal, M. Pharmacological activity of *Trachyspermum ammi* L. seeds essential oil grown from Northeast India. *J. Essent. Oil Bear. Plants* **2021**, *24*, 1373–1388.
28. Bahuguna, A.; Ramalingam, S.; Arumugam, A.; Natarajan, D.; Kim, M. Molecular and *in silico* evidences explain the anti-inflammatory effect of *Trachyspermum ammi* essential oil in lipopolysaccharide induced macrophages. *Process Biochem*. **2020**, *96*, 138–145.
29. Jayawantha, D.; Hettigoda, L.; Mudalige, T.D.; Paranagama, P.A. Exploring the bioactivity of siddhalepa asamodagam spirit from seeds of *Trachyspermum roxburghianum* (DC.) H. Wolff. *Nat. Prod. Commun*. **2024**, *19*, 1934578X241271629. https://doi.org/10.1177/1934578X241271629.
51. Wu, Y.-X.; Lu, W.-W.; Geng, Y.-C.; Yu, C.-H.; Sun, H.-J.; Kim, Y.-J.; Zhang, G.; Kim, T. Antioxidant, antimicrobial and anti-inflammatory activities of essential oil derived from the wild rhizome of *Atractylodes macrocephala*. *Chem. Biodivers*. **2020**, *17*, e2000268. https://doi.org/10.1002/cbdv.202000268.
52. Foudah, A.I.; Alqarni, M.H.; Alam, A.; Salkini, M.A.; Ahmed, E.O.I.; Yusufoglu, H.S. Evaluation of the composition and in vitro antimicrobial, antioxidant, and anti-inflammatory activities of Cilantro (*Coriandrum sativum* L. leaves) cultivated in Saudi Arabia (Al-Kharj). *Saudi J. Biol. Sci.* **2021**, *28*, 3461-3468.
53. Kumar, A.; Singh, S.; Kumar, A.; Bawankule, D.U.; Tandon, S.; Singh, A.K.; Verma, R.S.; Saikia, D. Chemical composition, bactericidal kinetics, mechanism of action, and anti-inflammatory activity of *Isodon melissoides* (Benth.) H. Hara essential oil. *Nat. Prod. Res*. **2021**, *35*, 690-695.
54. Nea, F.; Kambiré, D.A.; Genva, M.; Tanoh, E.A.; Wognin, E.L.; Martin, H.; Brostaux, Y.; Tomi, F.; Lognay, G.C.; Tonzibo, Z.F.; Fauconnier, M.-L. Composition, seasonal variation, and biological activities of *Lantana camara* essential oils from Côte d'Ivoire. *Molecules* **2020**, *25*, 2400. https://doi.org/10.3390/molecules25102400.
55. Nea, F.; Tanoh, E.A.; Wognin, E.L.; Kemene, T.K.; Genva, M.; Saive, M.; Tonzibo, Z.F.; Fauconnier, M.-L. A new chemotype of *Lantana rhodesiensis* Moldenke essential oil from Côte d'Ivoire: chemical composition and biological activities. *Ind. Crops Prod*. **2019**, *141*, 111766. https://doi.org/10.1016/j.indcrop.2019.111766.

56. Bayala, B.; Bassole, I.H.N.; Gnoula, C.; Nebie, R.; Yonli, A.; Morel, L.; Figueredo, G.; Nikiema, J.-B.; Lobaccaro, J.-M. A.; Simpore, J. Chemical composition, antioxidant, anti-inflammatory and anti-proliferative activities of essential oils of plants from Burkina Faso. *PLoS ONE* **2014**, *9*, e92122. https://doi.org/10.1371/journal.pone.0092122.
57. Su, Y.-C.; Hsu, K.P.; Ho, C.-L. Composition, in vitro anti-inflammatory, antioxidant and antimicrobial activities of rhe leaf essential oil of *Machilus konishii* from Taiwan. *Nat. Prod. Commun.* **2016**, *11*, 1363-1366.
58. Fraternale, D.; Dufat, H.; Albertini, M.C.; Bouzidi, C.; d'Adderio, Coppari, S.; Giacomo, B. ; Melandri, D.; Ramakrishbna, S.; Colomba, M. Chemical composition, antioxidant and anti-inflammatory properties of *Monarda didyma* L. essential oil. *PeerJ* **2022**, *10*, e14433. https://doi.org/10.7717/peerj.14433.
59. Kanyal, J.; Prakash, O.; Kumar, R.; Rawat, D.S. Essential oil composition and biological activities determination of *Mosla dianthera* (Buch.-Ham. ex Roxb.) Maxim. and its major isolated component, carvone. *Braz. J. Pharm. Sci.* **2022**, *58*, e201031. https://doi.org/10.1590/s2175-97902022e201031.
60. Kazemi, M. Phytochemical composition, antioxidant, anti-inflammatory and antimicrobial activity of *Nigella sativa* L. essential oil. . *J. Essent. Oil Bear. Plants*. **2014**, *17*, 1002-1011.
61. Yuan, Z.; Shafiq, M.; Zheng, H.; Zhang, L.; Wang, Z.; Yu, X.; Song, J.; Sun, B.; El-Newehy, M.; El-Hamshary, H.; Morsi, Y.; Wang, C.; Mo, X.; Xu, Y. Multi-functional fibrous dressings for infectious injury treatment with anti-adhesion wound healing. *Mater. Des.* **2023**, *235*, 112459. https://doi.org/10.1016/j.matdes.2023.112459.
62. Al-Mijalli, S.H.; Mrabti, N.N.; Ouassou, H.; Sheikh, R.A.; Assaggaf, H.; Bakrim, S.; Abdallah, E.M.; Alshahrani, M.M.; Al Awadh, A.A.; Lee, L.-H.; AlDhaheri, Y.; Sahegkar, A.; Zengin, G.; Attar, A.A.; Bouyahya, A.; Mrabti, H.N. Chemical composition and antioxidant, antimicrobial, and anti-inflammatory properties of *Origanum compactum* Benth essential oils from two regions: *in vitro* and *in vivo* evidence and *in silico* molecular investigations. *Molecules*. **2022**, *27*, 7329. doi.org/10.3390/molecules27217329.
63. Stojanović, N.M.; Mitić, K.V.; Nešić, M. ; Stanković, M. ; Petrović, V. ; Baralić, M. ; Randjelović, P.J. ; Soković, D. ; Radulović, N. Oregano (*Origanum vulgare*) essential oil and its constituents prevent rat kidney tissue injury and inflammation induced by a high dose of L-arginine. *Int. J. Mol. Sci.* **2024**, *25*, 941. https://doi.org/10.3390/ijms25020941.
64. Naccari, C.; Ginestra, G.; Micale, N.; Palma, E.; Galletta, B.; Costa, R.; Vadalà, R.; Nostro, A.; Cristani, M. Binary combinations of essential oils: antibacterial activity against *Staphylococcus aureus*, and antioxidant and anti-inflammatory properties. *Molecules* **2025**, *30*, 438. https://doi.org/10.3390/molecules30030438.
65. Carrasco, A.; Perez, E.; Cutillas, A.-B.; Martinez-Gutierrez, R.; Tomas, V.; Tudela, J. *Origanum vulgare* and *Thymbra capitata* essential oils from Spain: determination of aromatic profile and bioactivities. *Nat. Prod. Commun.* **2016**, *11*, 113-120.

6. Kazemi, M. Chemical composition and antimicrobial, antioxidant activities and anti-inflammatory potential of *Achillea millefolium* L., *Anethum graveolens* L., and *Carum copticum* L. essential oils. *J. Herb. Med.* **2015**, *5*, 217-222.

1. Wu, Y.-X.; Lu, W.-W.; Geng, Y.-C.; Yu, C.-H.; Sun, H.-J.; Kim, Y.-J.; Zhang, G.; Kim, T. Antioxidant, antimicrobial and anti-inflammatory activities of essential oil derived from the wild rhizome of *Atractylodes macrocephala*. *Chem. Biodivers*. **2020**, *17*, e2000268. DOI: 10.1002/cbdv.202000268.
2. Kazemi, M. Chemical composition, antimicrobial, antioxidant and anti-inflammatory activity of *Carum copticum* L. essential oil. *J. Essent. Oil Bear. Plants*. **2014**, *17(5)*, 1040-1045.
3. Foudah, A.I.; Alqarni, M.H.; Alam, A.; Salkini, M.A.; Ahmed, E.O.I.; Yusufoglu, H.S. Evaluation of the composition and in vitro antimicrobial, antioxidant, and anti-inflammatory activities of Cilantro (*Coriandrum sativum* L. leaves) cultivated in Saudi Arabia (Al-Kharj). *Saudi J. Biol. Sci.* **2021**, *28*, 3461-3468.
4. Hajhashemi, V.; Kopaei, S.R.; Sajjadi, S.E. Anti-nociceptive and anti-inflammatory effects of *Ferulago angulata*. *Immunopathol. Persa*. **2020**, *6*(2), e28. DOI:10.34172/ipp.2020.28.
5. Kumar, A.; Singh, S.; Kumar, A.; Bawankule, D.U.; Tandon, S.; Singh, A.K.; Verma, R.S.; Saikia, D. Chemical composition, bactericidal kinetics, mechanism of action, and anti-inflammatory activity of *Isodon melissoides* (Benth.) H. Hara essential oil. *Nat. Prod. Res*. **2021**, *35(4)*, 690-695.
6. Nea, F.; Kambiré, D.A.; Genva, M.; Tanoh, E.A.; Wognin, E.L.; Martin, H.; Brostaux, Y.; Tomi, F.; Lognay, G.C.; Tonzibo, Z.F.; Fauconnier, M.-L. Composition, seasonal variation, and biological activities of *Lantana camara* essential oils from Côte d'Ivoire. *Molecules* **2020**, *25*, 2400. doi:10.3390/molecules25102400.
7. Nea, F.; Tanoh, E.A.; Wognin, E.L.; Kemene, T.K.; Genva, M.; Saive, M.; Tonzibo, Z.F.; Fauconnier, M.-L. A new chemotype of *Lantana rhodesiensis* Moldenke essential oil from Côte d'Ivoire: chemical composition and biological activities. *Ind. Crops Prod*. **2019**, *141*, 111766. https://doi.org/10.1016/j.indcrop.2019.111766.
8. Bayala, B.; Bassole, I.H.N.; Gnoula, C.; Nebie, R.; Yonli, A.; Morel, L.; Figueredo, G.; Nikiema, J.-B.; Lobaccaro, J.-M. A.; Simpore, J. Chemical composition, antioxidant, anti-inflammatory and anti-proliferative activities of essential oils of plants from Burkina Faso. *PLoS ONE* **2014**, *9(3)*, e92122. doi:10.1371/journal.pone.0092122.
9. Su, Y.-C.; Hsu, K.P.; Ho, C.-L. Composition, in vitro anti-inflammatory, antioxidant and antimicrobial activities of rhe leaf essential oil of *Machilus konishii* from Taiwan. *Nat. Prod. Commun*. **2016**, *11(9)*, 1363-1366.
10. Fraternale, D.; Dufat, H.; Albertini, M.C.; Bouzidi, C.; d'Adderio, Coppari, S.; Giacomo, B. ; Melandri, D.; Ramakrishbna, S.; Colomba, M. Chemical composition, antioxidant and anti-inflammatory properties of *Monarda didyma* L. essential oil. *PeerJ* **2022**, *10*, e14433. DOI 10.7717/peerj.14433.
11. Kanyal, J.; Prakash, O.; Kumar, R.; Rawat, D.S. Essential oil composition and biological activities determination of *Mosla dianthera* (Buch.-Ham. ex Roxb.) Maxim. and its major isolated component, carvone. *Braz. J. Pharm. Sci*. **2022**, *58*, e201031. http://dx.doi.org/10.1590/s2175-97902022e201031.

12. Kazemi, M. Phytochemical composition, antioxidant, anti-inflammatory and antimicrobial activity of *Nigella sativa* L. essential oil. . *J. Essent. Oil Bear. Plants*. **2014**, *17(5)*, 1002-1011.
13. Bourgou, S.; Rebey, I.B. ; Kaab, S.B.; Hammami, M.; Dakhlaoui, S.; Sawsen, S.; Msaada, K.; Isoda, H.; Ksouri, R.; Fauconnier, M.-L. Green solvent to substitute hexane for bioactive lipids extraction from black cumin and basil seeds. *Foods* **2021**, *10*, 1493. https://doi.org/10.3390/foods10071493.
14. Mirahmad, A.; Ghoran, S.H.; Alipour, P.; Taktaz, F.; Hassan, S.; Naderian, M.; Moradalipour, A.; Faizi, M.; Kobarfard, F.; Ayatollahi, S.A. *Oliveria decumbens* Vent. (Apiaceae): biological screening and chemical compositions. *J. Ethnopharmacol*. **2024**, *318,* 117053. https://doi.org/10.1016/j.jep.2023.117053.
15. Yuan, Z.; Shafiq, M.; Zheng, H.; Zhang, L.; Wang, Z.; Yu, X.; Song, J.; Sun, B.; El-Newehy, M.; El-Hamshary, H.; Morsi, Y.; Wang, C.; Mo, X.; Xu, Y. Multi-functional fibrous dressings for infectious injury treatment with anti-adhesion wound healing. *Mater. Des*. **2023**, *235*, 112459. https://doi.org/10.1016/j.matdes.2023.112459.
16. Al-Mijalli, S.H.; Mrabti, N.N.; Ouassou, H.; Sheikh, R.A.; Assaggaf, H.; Bakrim, S.; Abdallah, E.M.; Alshahrani, M.M.; Al Awadh, A.A.; Lee, L.-H.; AlDhaheri, Y.; Sahegkar, A.; Zengin, G.; Attar, A.A.; Bouyahya, A.; Mrabti, H.N. Chemical composition and antioxidant, antimicrobial, and anti-inflammatory properties of *Origanum compactum* Benth essential oils from two regions: *in vitro* and *in vivo* evidence and *in silico* molecular investigations. *Molecules*. **2022**, *27*, 7329. doi.org/10.3390/molecules27217329.
17. Kerbouche, L.; Hazzit, M.; Ferhat, M.-A.; Baaliouamer, A.; Miguel, M.G. Biological activities of essential oils and ethanol extracts of *Teucrium polium* subsp. *capitatum* (L.) Briq. And *Origanum floribundum* Munby. *J. Essent. Oil Bear. Plants* **2015**, *18(5)*, 1197-1208.
18. Zinno, P.; Guantario, B.; Lombardi, G.; Ranaldi, G.; Finamore, A.; Allegra, S.; Mammano, M.M.; Fascella, G.; Raffo, A.; Roselli, M. Chemical composition and biological activities of essential oils from *Origanum vulgare* genotypes belonging to the carvacrol and thymol chemotypes. *Plants* **2023**, **12**, 1344. https://doi.org/10.3390/plants12061344.
19. Marrelli, M.M Araniti, F.; Abenavoli, M.R.; Statti, G.; Conforti, F. Potential health benefits of *Origanum heracleoticum* essential oil : phytochemical and biological variability among different Calabrian populations. *Nat. Prod. Commun*. **2018**, *13(9)*, 1183-1187.
20. Stojanović, N.M.; Mitić, K.V.; Nešić, M. ; Stanković, M. ; Petrović, V. ; Baralić, M. ; Randjelović, P.J. ; Sokolović, D. ; Radulović, N. Oregano (*Origanum vulgare*) essential oil and its constituents prevent rat kidney tissue injury and inflammation induced by a high dose of L-arginine. *Int. J. Mol. Sci*. **2024**, *25*, 941. https://doi.org/10.3390/ijms25020941.
21. Naccari, C.; Ginestra, G.; Micale, N.; Palma, E.; Galletta, B.; Costa, R.; Vadalà, R.; Nostro, A.; Cristani, M. Binary combinations of essential oils: antibacterial activity against *Staphylococcus aureus*, and antioxidant and anti-inflammatory properties. *Molecules* **2025**, *30*, 438. https://doi.org/10.3390/molecules30030438.

22. Carrasco, A.; Perez, E.; Cutillas, A.-B.; Martinez-Gutierrez, R.; Tomas, V.; Tudela, J. *Origanum vulgare* and *Thymbra capitata* essential oils from Spain: determination of aromatic profile and bioactivities. *Nat. Prod. Commun*. **2016**, *11(1)*, 113-120.
23. Abiodun, O.O.; Oke, T.A.; Adeyemi, F.O.; Oshinloye, A.O.; Akande, A.O. *Salacia pallescens* Oliv. (Celastraceae) scavenges free radicals and inhibits pro-inflammatory mediators in lipopolysaccharide-activated RAW cells 264.7 macrophages. *Turk. J. Pharm. Sci*. **2021**, *18(6)*, 702-709.
24. Jayawantha, D.; Hettigoda, L.; Mudalige, T.D.; Paranagama, P.A. Exploring the bioactivity of siddhalepa asamodagam spirit from seeds of *Trachyspermum roxburghianum* (DC.) H. Wolff. *Nat. Prod. Commun*. **2024**, *19(8)*, 1-15. DOI: 10.1177/1934578X241271629.
25. Elbouny, H.; Ouahzizi, B.; El-Guourrami, O.; Drioua, S.; Mbarek, A.N.; Sellam, K.; Alem, C. Chemical profile and biological properties of the essential oil of *Thymus atlanticus* (Ball) Roussine. *South Afr. J. Bot*. **2022**, *151*, 475-480.
26. Aazza, S.; El-Guendouz, S.; Miguel, M.G.; Antunes, M.D.; Faleiro, M.L.; Correia, A.I.; Figueiredo, A.C. Antioxidant, anti-inflammatory and anti-hyperglycaemic activities of essential oils from *Thymbra capitata*, *Thymus albicans*, *Thymus caespititius*, *Thymus carnosus*, *Thymus lotocephalus* and *Thymus mastichina* from Portugal. *Nat. Prod. Commun*. **2016**, *11(7)*, 1029-1038.
27. Chandra, M.; Prakash, O.; Bachheti, R.K.; Kumar, M.; Pant, A.K. Essential oil composition, phenolic constituents, antioxidant and pharmacological activities of *Thymus linearis* Benth. Collected from Uttarakhand region of India. *J. Essent. Oil Bear. Plants* **2016**, *19(2)*, 277-289.
28. Demirci, F.; Karaca, N.; Tekin, M.; Demirci, B. Anti-inflammatory and antibacterial evaluation of *Thymus sipyleus* Boiss. subsp. *sipyleus* var. *sipyleus* essential oil against rhinosinusitis pathogens. *Microb. Pathog*. **2018**, *122*, 117-121.
29. Pandur, E.; Micalizzi, G.; Mondello, L.; Horváth, A.; Sipos, K.; Horváth, G. Antioxidant and anti-inflammatory effects of thyme (*Thymus vulgaris* L.) essential oils prepared at different plant phenophases on *Pseudomonas aeruginosa* LPS-activated THP-1 macrophages. *Antioxidants* **2022**, *11*, 1330. doi.org/10.3390/antiox11071330.
30. Alshehri, K.M.; Abdella, E.M. Development of ternary nanoformulation comprising bee pollen-thymol oil extracts and chitosan nanoparticles for anti-inflammatory and anticancer applications. *Int. J. Biol. Macromol*. **2023**, *242*, 124584. https://doi.org/10.1016/j.ijbiomac.2023.124584.
31. Warman, D.J.; Jia, H.; Kato, H. Effects of thyme (*Thymus vulgaris* L.) essential oil on aging-induced brain inflammation and blood telomere attrition in chronologically aged C57BL/6J mice. *Antioxidants* **2023**, *12*, 1178. ttps://doi.org/10.3390/antiox12061178.
32. Spréa, R.M.; Caleja, C. ; Finimundy, T.C.; Calhelha, R.C.; Pires, T.C.S.P.; Amaral, J.S. ; Prieto, M.A.; Ferreira, I.C.F.R.; Pereira, E.; Marros, L. Chemical and bioactive evaluation of essential oils from edible and aromatic Mediterranean Lamiaceae plants. *Molecules* **2024**, *29*, 2827. https://doi.org/10.3390/molecules29122827.

33. Gupta, N.; Bhattacharya, S. ; Dutta, A. ; Tauchen, J. ; Landa, P. ; Urbanová, K. ; Houdková, M. ; Fernández-Cusimamani, E. ; Leuner, O. Synthetic polyploidization induces enhanced phytochemical profile and biological activities in *Thymus vulgaris* L. essential oil. *Sci. Rep.* **2024**, *14*, 5608. https://doi.org/10.1038/s41598-024-56378-7.
34. Abdelli, W.; Bahri, F.; Romane, A.; Höferl, M.; Wanner, J.; Schmidt, E.; Jirovetz, L. Chemical composition and anti-inflammatory activity of Algerian *Thymus vulgaris* essential oil. *Nat. Prod. Commun*. **2017**, *12(4)*, 611-614.
35. Cutillas, A.-B.; Carrasco, A.; Martinez-Gutierrez, R.; Tomas, V.; Tudela, J. Thyme essential oils from Spain: aromatic profile ascertained by GC-MS, and their antioxidant, anti-lipoxygenase and antimicrobial activities. *J. Food Drug. Anal*. **2018**, *26*, 529-544.
36. Rodrigues, V.; Cabral, C.; Évora, L.; Ferreira, I.; Cavaleiro, C.; Cruz, M.T.; Salgueiro, L. Chemical composition, anti-inflammatory activity and cytotoxicity of *Thymus zygis* L. subsp. *sylvestris* (Hoffmanns. & Link) Cout. Essential oil and its main compounds. *Arab. J. Chem*. **2019**, *12*, 3236-3243.
37. Vanitha, K.G.; Natarajan, A.; Sudhkar, N.; Hirad, A.H; Alarfaj, A.A.; Arulselvan, P.; Raja, R. Enhancing therapeutic potential: investigating traditional detoxification methods and assessing their influence on anti-microbial efficacy, phytochemical composition, heavy metal content and anti-inflammatory properties in *Trachyspermum ammi*. *Ind. J. Pharm. Edu. Res.* **2025**, *59(1)*, 230-241.
38. Dutta, P.; Sarma, N.; Saikia, S.; Gogoi, R.; Begum, T.; Lal, M. Pharmacological activity of *Trachyspermum ammi* L. seeds essential oil grown from Northeast India. *J. Essent. Oil Bear. Plants* **2021**, *24(6)*, 1373-1388.
39. Bahuguna, A.; Ramalingam, S.; Arumugam, A.; Natarajan, D.; Kim, M. Molecular and in silico evidences explain the anti-inflammatory effect of *Trachyspermum ammi* essential oil in lipopolysaccharide induced macrophages. *Process Biochem*. **2020**, *96*, 138-145.
